# The role of diet in renal cell carcinoma incidence: an umbrella review of meta-analyses of observational studies

**DOI:** 10.1186/s12916-021-02229-5

**Published:** 2022-02-03

**Authors:** Zhanchen Liao, Zhitao Fang, Siqi Gou, Yong Luo, Yiqi Liu, Zhun He, Xin Li, Yansong Peng, Zheng Fu, Dongjin Li, Haiyun Chen, Zhigang Luo

**Affiliations:** 1 The Second Affiliated Hospital, Institute of Urology and Organ Transplantation, Hengyang Medical School, University of South China, Hengyang, 421001 Hunan China; 2 The Second Affiliated Hospital, Trauma Center & Critical Care Medicine, Hengyang Medical School, University of South China, Hengyang, 421001 Hunan China

**Keywords:** Renal cell carcinoma, Diet, Nutrition, Dietary pattern, Foods, Food group, Beverage, Macronutrient, Micronutrient, Umbrella review

## Abstract

**Background:**

Evidence associating diet with the incidence of renal cell carcinoma (RCC) is inconclusive. We aimed to summarize evidence associating dietary factors with RCC incidence and assess the strength and validity of this evidence.

**Methods:**

We conducted an umbrella review of systematic reviews or meta-analyses (SRoMAs) that assessed the association between diet and RCC incidence. Through April 2021, PubMed, Web of Science, Embase, The Cochrane Library, Scopus, and WCRF were searched. Two independent reviewers selected studies, extracted data, and appraised the quality of SRoMAs. According to credibility assessment criteria, evidence can be divided into five categories: convincing (class I), highly suggestive (class II), suggestive (class III), weak (class IV), and nonsignificant (class V).

**Results:**

Twenty-nine meta-analyses were obtained after screening. After excluding 7 overlapping meta-analyses, 22 meta-analyses including 502 individual studies and 64 summary hazard ratios for RCC incidence were included: dietary patterns or dietary quality indices (*n* = 6), foods (*n* = 13), beverages (*n* = 4), alcohol (*n* = 7), macronutrients (*n* =15), and micronutrients (*n* =19). No meta-analyses had high methodological quality. Five meta-analyses exhibited small study effects; one meta-analysis showed evidence of excess significance bias. No dietary factors showed convincing or highly suggestive evidence of association with RCC in the overall analysis. Two protective factors had suggestive evidence (vegetables (0.74, 95% confidence interval 0.63 to 0.86) and vitamin C (0.77, 0.66 to 0.90)) in overall analysis. One protective factor had convincing evidence (moderate drinking (0.77, 0.70 to 0.84)) in Europe and North America and one protective factor had highly suggestive evidence (cruciferous vegetables (0.78, 0.70 to 0.86)) in North America.

**Conclusions:**

Although many meta-analyses have assessed associations between dietary factors and RCC, no high-quality evidence exists (classes I and II) in the overall analysis. Increased intake of vegetables and vitamin C is negatively associated with RCC risk. Moderate drinking might be beneficial for Europeans and North Americans, and cruciferous vegetables might be beneficial to North Americans, but the results should be interpreted with caution. More researches are needed in the future.

**Trial registration:**

PROSPERO CRD42021246619

**Supplementary Information:**

The online version contains supplementary material available at 10.1186/s12916-021-02229-5.

## Background

Renal cell carcinoma (RCC) is the most fatal cancer of the urinary system and accounts for approximately 3% of all adult malignancies [1, 2]. The incidence of RCC is higher in developed countries [3, 4]. From 2013 to 2017, the incidence of RCC showed an increasing trend, with an average annual increase of 1.4% in men (average annual percent change=1.4%, 95% confidence interval, CI =1.0 to 1.7%) and 1.6% in women (1.6%, 0.6 to 2.7%) [5]. In 2020, there were approximately 430,000 new RCC cases and 180,000 deaths worldwide [6]. In 2021, it is estimated that approximately 76,000 new cases of RCC and 13,000 deaths will occur in the USA [7].

Treatment of RCC results in a heavy financial burden. According to the 2017 French health insurance database, the cost of targeted therapy for patients with advanced RCC is €5546/person/month [8]. Therefore, effective prevention and management of RCC are necessary to significantly reduce the financial burden for the country and patients’ family and is of great public health importance. Although many factors (including diet) have been suggested to be associated with the incidence of RCC, the conclusions are inconsistent [9].

Numerous studies have shown that diet is inextricably linked to the tumorigenesis of RCC [10–14]. Evidence regarding the association of various dietary factors, such as red meat, vegetables, and vitamin B6, with the risk of RCC has been summarized in published SRoMAs. These discoveries could have important implications for RCC prevention. However, the strength and validity of this evidence and whether there is a potential risk of bias still need to be further evaluated and analyzed. In addition, previously published meta-analyses associating a specific dietary factor with RCC incidence have had conflicting findings. For example, previously published meta-analyses investigated the correlation between red meat and processed meat intake and the incidence of RCC [15–18]. According to Alexander and Lee, red meat and processed meat consumption were not related to the risk of RCC [16, 18], whereas Faramawi and Zhang concluded that eating more red meat and processed meat increased the risk of RCC [15, 17]. Furthermore, the associations reported in these studies may be causal, but the effect of these diets on RCC risk could be overstated because of the inherent bias associated with inevitable confounding factors and selective reporting of positive results [19–22].

To summarize and assess the strength and validity of the available evidence and to evaluate its quality, an umbrella review of SRoMAs of observational studies was conducted to investigate the associations between various dietary factors (including dietary patterns or dietary quality indices, different foods, beverages, alcohol, and various nutrients) and the incidence of RCC. A systematic evaluation of the available data from published SRoMAs on a particular research issue is referred to as an umbrella review [23–32]. Umbrella reviews can assess the strength and validity of the evidence in the published SRoMAs and evaluate the potential for risk of bias [33].

## Methods

The protocol was registered in PROSPERO (registration number: CRD42021246619), and our search strategy followed the PRISMA 2020 statement: an updated guideline for reporting systematic reviews [34].

### Inclusion and exclusion criteria

The inclusion criteria were as follows: (1) dose-response or high vs low SRoMAs of observational studies (including case-control studies, prospective cohort studies, or pooled studies); (2) SRoMAs reporting odds ratios, relative rates, risk ratios, or hazard ratios as effect sizes, and SRoMAs including summary hazard ratios (SHRs) and 95% CIs. PICO definitions: (1) population: people of any sex, age, source of cases, country, and ethnicity; (2) intervention: dietary factors including various foods, dietary patterns, dietary quality index, beverages, alcohol, macronutrients, and micronutrients (see Additional file 1: Table S1); (3) comparator: the abovementioned dietary factors versus no dietary factors or varying levels of dietary factors (dose-response or high vs low); and (4) outcome: patients with RCC.

Studies that met any of the following criteria were excluded: (1) individual study, (2) animal or experimental study, (3) conference literature, (4) editorial, (5) systematic review without meta-analysis (i.e., no combined effect size), (6) meta-analysis of individual patient data, (7) exposures were plasma levels or biomarkers, and (8) diseases were other urologic tumors.

### Search strategy

PubMed, Web of Science, Embase, Scopus, and The Cochrane Library were systematically searched using a predefined search strategy from inception until April 2021. We also manually searched World Cancer Research Fund (WCRF) for their SRoMAs, which was rigorously conducted by their groups of experts. The language was limited to English. The literature search was conducted independently by two reviewers (ZCL, ZTF). Disagreements during the search process were resolved by consensus through discussion. A detailed search strategy for the Embase database has been provided in Additional file 1: Table S1. The Cohen kappa analysis was performed to measure the level of agreement between the reviewers on the selection of eligible studies.

### Study selection and data extraction

Study selection and data extraction were conducted by one author (ZCL) and double-checked by another author (ZTF). When a disagreement emerged, a consensus was reached through discussion. For each published SRoMA, data extracted included the title, first author, year of publication, exposures (including doses), number and types of individual studies included, type of effect model, sample size (participants and cases), type of meta-analysis metric, summary estimated effect (odds ratios, relative rates, risk ratios, or hazard ratios) and 95% CI, type of comparison, quality appraisal tool for the individual study, publication bias, heterogeneity, funding information, and conflict of interest. For SRoMAs examining the associations between a specific dietary factor and multiple diseases or health outcomes, only data related to RCC were extracted.

The following data were extracted from individual studies in a SRoMA: first author, publication year, exposures (including doses), sample size (participants and cases), summary estimated effect that adjusted for the most confounders and 95% CI, and adjusted covariates. According to the literature, the most important potential confounders in the investigation between dietary factors and the risk of RCC include age, sex, smoking, BMI, total energy intake, alcohol intake, and physical activity. We utilized the form of the Joanna Briggs Institute as our data extraction guide (see Additional file 1: Table S2) [35]. The effect sizes and 95% CI were used to estimate small study effects and excess significant bias. If the data were not available in the articles and appendices, the author were contacted to request the missing information.

### Assessment of methodological quality

SRoMAs that satisfied the inclusion criteria were appraised by one author (ZCL) utilizing the AMSTAR 2 online quality appraisal instrument and providing an overall rating for the SRoMAs included [36, 37]. Verification of the quality assessment process was performed by another author (ZTF). Disagreements were addressed by discussion and consensus.

AMSTAR 2 is a reliable and validated methodological quality appraisal instrument with 16 items [38]. Seven of these items are critical. These 7 critical items are whether the protocol was registered before starting the systematic review, whether a comprehensive and adequate literature search was conducted, whether the exclusion of individual studies was justified, whether the risk of bias of the individual studies included in the systematic review was adequately assessed, what statistical procedures were employed in the meta-analysis and whether or not they were appropriate, whether the risk of bias was adequately considered when analyzing the results of the systematic review, and whether publication bias and its possible impact were adequately assessed [36].

### Outdated and overlapping publications

SRoMAs are continually updated by incorporating relevant evidence from new studies to maintain validity and applicability. Otherwise, there would be a risk of inaccurate evidence or even a situation where erroneous evidence misleads decision-makers or researchers [39–42]. Moreover, different investigators or research institutions perform SRoMAs of the same exposure and population. Therefore, there can be two or more overlapping SRoMAs on the same research topics [43, 44]. Some studies have reported that up to 2/3 of published SRoMAs overlap at least partially or completely [45]. Inclusion of data from overlapping SRoMAs would lead to a risk of bias, resulting in inaccurate results and estimates [46, 47]. In addition, considering that up to 50% of published systematic reviews become outdated after 5.5 years [42], we used 2015 as the cutoff year. We referred to overlapping SRoMAs published before 2015 as outdated overlapping SRoMAs and overlapping SRoMAs published after 2015 as contemporary overlapping SRoMAs. If a contemporary SRoMA overlapped with an outdated SRoMA, the outdated SRoMA was excluded.

As in the previous umbrella review [26], in the case of contemporary overlapping SRoMAs, we used the citation matrix (graphical cross tabulation) recommended by Bougioukas et al. to show the degree of overlap [44]. The corrected covered area (CCA) was used to quantify the degree of overlap of the citation matrix [43]. The degree of overlap was labeled as slight, moderate, high, or very high, corresponding to CCAs of 0 to 5%, 6 to 10%, 11 to 15%, and greater than 15%, respectively. Details of the overlapping SRoMAs have been shown in Additional file 1: Table S3. The selection of overlapping SRoMAs will be based on the following principles: (1) if the overlapping publications were derived from both Cochrane systematic reviews and non-Cochrane systematic reviews, the former was preferred [48] because these tended to be more up-to-date and of higher quality than the latter [49]; (2) if the overlapping publications were derived from non-Cochrane systematic reviews and the degree of overlap was high or very high, the following SRoMAs were preferred: the reviews with the best methodological quality after assessment with AMSTAR 2, the most recently published reviews, the reviews with the largest number of individual studies included, or the reviews with the largest number of participants [48]; and (3) if the degree of overlap was moderate or slight, both reviews were retained.

If the overlapping SRoMAs were all outdated, discarding them directly would result in inadequate data utilization, which would lead to a risk of bias and inaccurate results, so the outdated overlapping SRoMAs were selected according to the 3 principles mentioned above for managing contemporary overlapping SRoMAs.

### Credibility assessment

As in previous umbrella reviews [23, 24, 27, 29, 30], we classified evidence associating diet and RCC incidence into 5 categories. Evidence was judged to be convincing (class I) if the following criteria were met: statistically significant results were observed for both the association and the largest study (*p*<10^-6^ and *p*<0.05, respectively), the number of cases exceeded 1000 or the number of participants for consecutive outcomes exceeded 20,000, there was low heterogeneity (*I*^2^<50%), the 95% prediction interval (PI) did not include a null value, there was no excess significance bias, and there were no small study effects (“*p*<0.10 of Egger’s regression” plus “the random-effects summary estimate being larger than the point estimate of the largest study in the meta-analysis” can be considered as small study effects). Evidence was judged to be highly suggestive (class II) if the following criteria were met: statistically significant results were observed for both the association and the largest study (*p*<10^-6^ and *p*<0.05, respectively); and the number of cases exceeded 1000 or the number of participants for consecutive outcomes exceeded 20,000. Evidence was judged to be suggestive (class III) if the following criteria were met: associations with statistical significance were observed for the association (*p*<10^-3^); the number of cases exceeded 1000 or the number of participants for consecutive outcomes exceeded 20,000. Evidence was judged to be weak (class IV) if it met the criterion of a statistically significant association (*p*<0.05). The evidence was judged to be nonsignificant (class V) if no statistically significant association was observed (*p*≥0.05)

### Statistical analysis

#### Assessment of SHR

We recalculated the SHR and corresponding 95% CI for the included SRoMAs for each exposure using the Sidik and Jonkman random-effects model [50, 51], which is recommended by the Cochrane handbook. It takes into account heterogeneity both within and between studies and adjusts the confidence interval [50, 51]. There may be high heterogeneity in the definition of a specific dietary factor and the sample characteristics of the individual studies included in the SRoMAs, and most of the included SRoMAs used random-effects model for combining effect sizes. To establish comparability with these previously published SRoMAs, we also adopted this approach. If the included SRoMAs were divided into two subgroups by sex or race to obtain hazard ratios, we combined the hazard ratios of the two subgroups using a fixed effects model before calculating the SHR and corresponding 95% CI [52].

If both dose-response and high vs low SRoMAs were available, we recalculated the SHR and corresponding 95% CI only for the SRoMA with the highest number of included individual studies. If data related to the dose-response SRoMAs were missing, we calculated the SHR and corresponding 95% CI for the high vs low SRoMAs. If data could not be extracted from the SRoMAs, we could not recalculate the SHR and corresponding the 95% CI, and therefore, we directly used the SHR and 95% CI from the included SRoMAs.

We considered that dietary factors were not associated with the incidence of RCC if the 95% CI included a null value or an SHR of 1. We used the meta package in R to calculate the SHR, 95% CI, and *p* value for each exposure [53].

#### Assessment of heterogeneity

We used *I*^2^ to assess the heterogeneity of each published SRoMA. *I*^2^ values range from 0 to 100% and are used as a measure of the magnitude of heterogeneity among individual studies and can be used to present the percentage of the total variance caused by heterogeneity [54]. *I*^2^ <50% can be considered nonsignificant heterogeneity. *τ*^2^ was also calculated, which can explain the between-study variance associated with risk estimates because *I*^2^ is affected by study size [55]. In addition, we calculated a 95% PI for the SHR, which further explains the heterogeneity among studies. This interval with 95% certainty provides a prediction range for the potential true effect size of future studies [55]. The *I*^2^, *τ*^2^, and 95% PI for each published SRoMA were calculated with the meta package in R [53].

#### Assessment of publication bias and small study effects

For published SRoMAs, contour-enhanced funnel plots were used to assess whether indications of publication bias existed [56]. The presence of missing studies in areas of the plot without significant differences suggests that the funnel plot asymmetry may be due to publication bias; the presence of missing studies in areas with significant differences suggests that the asymmetry may be due to other causes, such as heterogeneity [57], rather than publication bias [56].

Egger’s regression asymmetry test was employed to see if indications of small study effects existed (i.e., small studies provide greater effect size estimates than large studies) [57]. As in prior umbrella reviews [23–25], a *p* value<0.10 with a more conservative effect in the largest study (the study with the smallest standard error) than in the random-effects meta-analysis was deemed to be indicative of small study effects. To estimate true effects, the “trim-and-fill” approach was applied when Egger’s test was statistically significant [58]. The contour-enhanced funnel plots, Egger’s test, and the trim-and-fill method were conducted with the meta and metafor packages in R [53, 59].

#### Assessment of excess significance bias

Based on a chi-square test, the excess significance test was used to assess whether the observed number of statistically significant studies (O) exceeded the expected number of statistically significant studies (E) [60, 61]. For the excess significance test, assuming that the true effect size is consistent with that of the largest study in the SRoMA, we calculated the statistical power of each individual study based on an algorithm that applies a noncentral *t* distribution [62]. The expected number (E) was calculated by adding the estimated statistical power of each individual study together. A *p* value<0.10 (two-tailed) for the observed number (O) > expected number (E) implies an excess significance bias, as described in previously published umbrella reviews [23, 24, 27]. The excess significance bias test was conducted with the metaviz package in R [63].

#### Subgroup analysis and sensitivity analysis

Where sample sizes permitted, subgroup analyses were performed by region and study design. For associations with evidence levels of classes I to III, we performed sensitivity analyses by removing small-sized studies and low-quality studies which were defined in the included SRoMA. We conducted all statistical analyses in R (version 4.0.5).

##### Patient involvement

There were no patients involved in any part of this umbrella review, including establishing the research questions, outcome measures, study design, or implementation; interpretation of results; and suggestions for manuscript writing. We will disseminate the results through social media and relevant conferences.

## Results

**Fig. 1:**
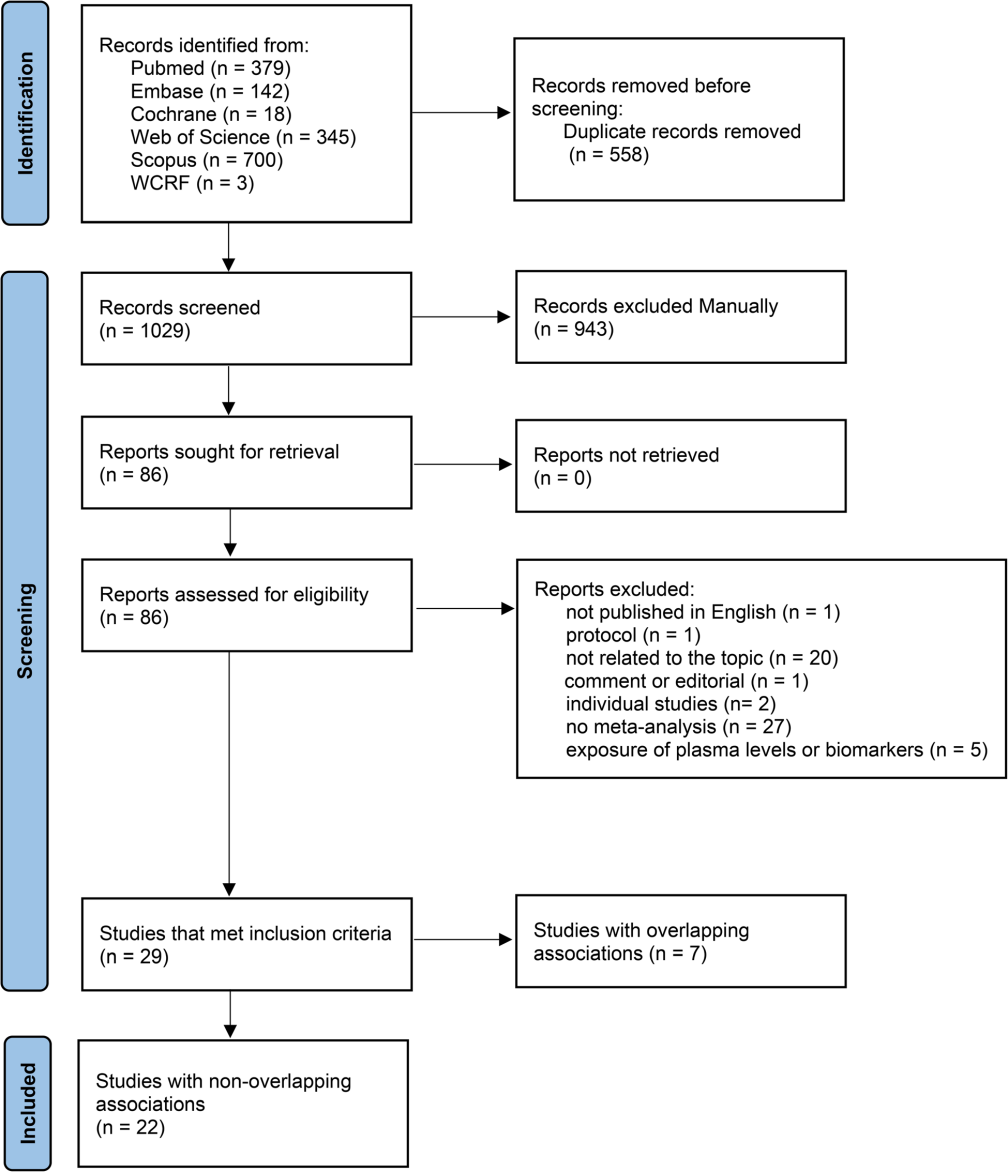
Flow diagram of the selection process

A total of 1587 publications were identified in six databases, and 1029 publications were obtained after excluding duplicates. After title and abstract screening, 86 publications required full-text screening. Twenty-nine published meta-analyses were obtained after full-text screening [15–18, 64–88]. After excluding 7 overlapping publications, 22 meta-analyses were ultimately included (kappa=0.87) [15–17, 64–81, 87]. The literature selection procedure is shown in Fig. 1, and details of the studies excluded after full-text screening and the reasons for exclusion are provided (see Additional file 1: Table S4) [18, 82–86, 88–145]. Twenty-two published meta-analyses included 502 individual studies and 64 SHRs for the incidence of RCC: dietary patterns or dietary quality indices (*n*=6), foods (*n*=13), beverages (*n*=4), alcohol (*n*=7), macronutrients (*n*=15), and micronutrients (*n*=19).

We identified the following exposures associated with the incidence of RCC: healthy dietary pattern [64], unhealthy/Western pattern [64], drinking pattern [64], fish [65], total fat [87], sweetened carbonated beverage [66], beer [67], wine [67], spirits [67], riboflavin [68], vitamin B6 [68], folate [68], vitamin B12 [68], saturated fat [87], methionine [68], choline [68], betaine [68], all meats [15], tea [69], fruit fiber [70], vegetable fiber [70], cereal fiber [70], legume fiber [70], dietary inflammatory index [71], vitamin C [72], seafood [16], animal fat [16], cholesterol [16], total protein [87], animal protein [87], plant fat [16], plant protein [87], sweetened beverages (including artificially sweetened beverages and sugar-sweetened beverages) [73], polyunsaturated fat [87], vitamin E [74], coffee [75], vitamin D [76], dietary nitrate [77], dietary nitrite [77], glycemic index [78], glycemic load [78], monounsaturated fat [87], dietary fiber [78], alcohol (light) [79], alcohol (moderate) [79], alcohol (heavy) [79], alcohol (any) [79], vegetables [80], fruit [80], red meat [17], processed meat [17], cruciferous vegetables [81], poultry [15, 16], fruit and non-starchy vegetables [87], non-starchy vegetables [87], tomatoes [87], citrus fruit [87], alpha-carotene [87], beta-cryptoxanthin [87], lutein and zeaxanthin [87], dietary lycopene [87], total calcium (food and supplements) [87], dietary calcium [87], and calcium (supplements) [87].

### Characteristics of the included meta-analyses

The overall characteristics of the 22 meta-analyses that were included in the umbrella review are summarized in Table 1. The first authors of the meta-analyses were from China, France, Iran, Spain, the UK, and the USA. The populations in the individual studies included in the meta-analyses were from Asia, North America, South America, Europe, and Oceania. The umbrella review included a total of 502 individual studies, of which 59% were cohort studies (*n*=298), 39% were case-control studies (*n*=196), and 2% were pooled studies (*n*=8).

**Table 1:** Overall characteristics of meta-analyses included in the umbrella review

Author/Year	Country	Exposure (including dose of exposure)	No. of included studies	Cohort	Case control	Pooled study	Effects model	No. of participants/no. of cases	MA metric	Comparison	Range/amount	Quality appraisal tool	Information on funding	Conflict of interests
Alizadeh 2017 [64]	Iran	Healthy dietary pattern	4	1	3	0	fixed	51,709/1327	OR	High vs low	Higher adherence vs lower adherence	NOS	NA	No
		Unhealthy/western pattern	4	1	3	0		51,709/1327	OR					
		Drinking pattern	2	1	1	0		47,705/554	OR					
Bai 2013 [65]	China	Fish	15	3	12	0	fixed	608,753/9324	RR	High vs low	Higher adherence vs lower adherence	NOS	No	No
Boyle 2014 [66]	France	Sweetened carbonated beverage	5	2	2	1	random	NA/NA	RR	High vs low	Higher adherence vs lower adherence	NA	Yes	No
Cheng 2011 [67]	China	Beer	8	0	8	0	fixed	NA/5538	OR	High vs low	Higher adherence vs lower adherence	NA	NA	NA
		Wine	8	0	8	0	random	NA/5538	OR					
		Spirits	9	0	9	0	random	NA/6393	OR					
Clasen 2020 [68]	UK	Riboflavin	3	1	2	0	Bayesian	39,789/1971	RR	High vs low	Higher adherence vs lower adherence	NOS	Yes	No
		Vitamin B6	5	3	2	0		164,883/2407	RR					
		Folate	6	2	4	0		136,319/3166	RR					
		Vitamin B12	3	2	1	0		127,390/1578	RR					
		Methionine	2	2	0	0		125,094/436	RR					
		Choline	2	2	0	0		125,094/436	RR					
		Betaine	2	2	0	0		125,094/436	RR					
Faramawi 2007 [15]	USA	All meat	11	0	11	0	fixed	15,237/4835	OR	High vs low	Higher adherence vs lower adherence	NA	NA	NA
Hu 2013 [69]	China	Tea	12	2	10	0	random	1,571,852/6636	RR	High vs low	Higher adherence vs lower adherence	NA	Yes	NA
Huang 2014 [70]	China	Fruit fiber	3	1	2	0	random	496,292/2906	RR	High vs low	Higher adherence vs lower adherence	NOS	NA	No
		Vegetable fiber	3	1	2	0		496292/2906	RR					
		Cereal fiber	3	1	2	0		496,292/2906	RR					
		Legume fiber	1	1	0	0		491,841/1816	RR					
Jayedi 2018 [71]	Iran	Dietary Inflammatory Index	2	1	1	0	random	36,118/1030	RR	Dose-response	1-unit increment	NOS	No	No
Jia 2015 [72]	China	Vitamin C	10	3	7	0	fixed	274,864/5182	RR	High vs low	Higher adherence vs lower adherence	NA	NA	No
Lee 2008 [16]	USA	Total fat	13	13	0	0	random	774,952/1478	RR	High vs low	Higher adherence vs lower adherence	NA	YES	NA
		Saturated fat	13	13	0	0		774,952/1478	RR					
		Monounsaturated fat	13	13	0	0		774952/1478	RR					
		Polyunsaturated fat	13	13	0	0		774952/1478	RR					
		Animal fat	8	8	0	0		417,093/900	RR					
		Plant fat	8	8	0	0		417,093/900	RR					
		Cholesterol	13	13	0	0		774,952/1478	RR					
		Total protein	13	13	0	0		774,952/1478	RR					
		Animal protein	8	8	0	0		530,410/1019	RR					
		Plant protein	8	8	0	0		530,410/1019	RR					
		Poultry	12	12	0	0		NA/1407	RR					
		Seafood	12	12	0	0		NA/1375	RR					
Llaha 2021 [73]	Spain	Sweet beverages (include ASB and SSB)	3	1	2	0	random	143,194/1589	RR	High vs low	Higher adherence vs lower adherence	ROBINS-E NOS	Yes	No
Shen 2015 [74]	China	Vitamin E	13	6	7	0	NA	471,006/6944	RR	High vs low	Higher adherence vs lower adherence	NA	Yes	No
Wijarnpreecha 2017 [75]	USA	Coffee	22	6	16	0	random	1,991,863/NA	RR	High vs low	Higher adherence vs lower adherence	NOS	No	No
Wu 2020 [76]	China	Vitamin D	6	3	3	0	fixed	187,560/NA	RR	High vs low	Higher adherence vs lower adherence	NOS	NA	No
Xie 2016 [77]	China	Dietary nitrate	3	2	1	0	random	NA/NA	RR	high vs low	Higher adherence vs lower adherence	NO	NA	No
		Dietary nitrite	2	1	1	0	fixed	NA/NA	RR					
Xu 2019 [78]	China	Dietary fiber	7	2	5	0	random	942,582/6590	RR	high vs low	Higher adherence vs lower adherence	NOS	Yes	No
		Glycemic index	5	2	3	0		502,119/4281	RR					
		Glycemic load	5	2	3	0		502119/4281	RR					
Xu 2015 [79]	China	Alcohol (light)	6	5	0	1	random	3,720,850/5214	RR	Light	<12.5 g/day (<1 drink/day)	NOS	Yes	No
		Alcohol (moderate)	8	7	0	1		4,139,301/5364	RR	Moderate	12.5–37.5 g/day (2–3 drinks/day)			
		Alcohol (heavy)	3	3	0	0		2,065,995/363	RR	Heavy	>37.5 g/day (>3 drinks/day)			
		Alcohol (any)	8	7	0	1		4,867,646/5503	RR	High vs low	Higher adherence vs lower adherence			
Zhang 2017 [80]	China	Vegetables	16	2	13	1	random	1,365,634/9353	RR	High vs low	Higher adherence vs lower adherence	NOS	NA	No
		Fruits	17	3	13	1		1,398,778/9150	RR					
Zhang 2017 [17]	China	Red meat	19	4	14	1	random	1,826,171/12631	RR	High vs low	Higher adherence vs lower adherence	NOS	NA	No
		Processed meat	19	3	15	1		1,793,675/14240	RR					
Zhao 2013 [81]	China	Cruciferous Vegetables	12	6	6	0	random	1,228,518/5773	RR	High vs low	Higher adherence v lower adherence	NOS	No	No
WCRF 2013 [87]	UK	Fruit and non-starchy vegetables	7	7	0	0	random	756,544/1215	RR	Dose-response	Per 100 g/d	NA	Yes	NA
		Non-starchy vegetables	8	8	0	0		1,248,385/3031		Dose-response	Per 100 g/d			
		Tomatoes	3	3	0	0		NA/427		Dose-response	Per 50 g/d			
		Citrus fruit	7	7	0	0		NA/2735		Dose-response	Per 50 g/d			
		Total fat	14	14	0	0		1,210,245/1985		Dose-response	5% energy			
		Saturated fat	14	14	0	0		1,210,245/1985		Dose-response	5% energy			
		Monounsaturated fat	14	14	0	0		1,210,245/1985		Dose-response	5% energy			
		Polyunsaturated fat	14	14	0	0		1,210,245/1985		Dose-response	5% energy			
		Total protein	14	14	0	0		1,210,245/1985		Dose-response	5% energy			
		Animal protein	14	14	0	0		1,210,245/1985		Dose-response	5% energy			
		Plant protein	14	14	0	0		1,210,245/1985		Dose-response	5% energy			
		Alpha-carotene	4	4	0	0		284,501/787		Dose-response	Per 600 μg/d			
		Beta-cryptoxanthin	4	4	0	0		284,501/787		Dose-response	Per 100 μg/d			
		Lutein and zeaxanthin	4	4	0	0		284,501/787		Dose-response	Per 1000 μg/d			
		Lycopene	4	4	0	0		284,501/787		Dose-response	Per 4000 μg/d			
		Total calcium (food and supplements)	3	3	0	0		NA/1711		Dose-response	Per 200 mg/d			
		Dietary calcium	3	3	0	0		NA/1574		Dose-response	Per 200 mg/d			
		Calcium from supplements	3	3	0	0		NA/1482		High vs low	Higher adherence v lower adherence			

*MA* meta-analysis, *NA* not available, *NOS* Newcastle-Ottawa Scale, *ROBINS-E* risk of bias in non-randomized studies—of exposures, *OR* odds ratio, *RR* risk ratio, *UK* The United Kingdom, *USA* The United States of America, *ASB* artificially sweetened beverages, *SSB* sugar-sweetened beverages

Except for legume fiber, the meta-analyses of each exposure included 7 (range 2–22) individual studies. Of the meta-analyses corresponding to the 64 exposures, 33% (*n*=21) were dose-response meta-analyses (dietary inflammatory index, alcohol (light), alcohol (moderate), alcohol (heavy), total fat, saturated fat, monounsaturated fat, polyunsaturated fat, total protein, animal protein, plant protein, fruit and non-starchy vegetables, non-starchy vegetables, tomatoes, citrus fruit, alpha-carotene, beta-cryptoxanthin, lutein and zeaxanthin, dietary lycopene, total calcium (food and supplements), dietary calcium), and 67% (*n*=43) were high vs low meta-analyses. Of the 64 exposures, 73% (*n*=47) of the meta-analyses combined hazard ratios by using a random effects model, 14% (*n*=9) used a fixed effects model to combine hazard ratios, 11% (*n*=7) used a Bayesian model to combine hazard ratios, and 2% (*n*=1) did not provide this information. Of the 22 meta-analyses, 59% (*n*=13) used the Newcastle-Ottawa Scale (NOS) to assess the methodological quality of the individual studies, one of which used “the Risk Of Bias In Non-randomized Studies—of Exposures” tool in addition to the NOS to assess the methodological quality of the individual studies, and 41% (*n*=9) did not report a relevant methodological quality assessment instrument (*n*=8) or did not use a relevant tool to assess methodological quality (*n*=1).

### Methodological quality

Detailed information on the methodological quality of the 29 meta-analyses assessed using ASMTAR 2 has been provided (see Additional file 1: Table S5) [15–18, 64–88]. Of the 29 meta-analyses, none were rated as having high methodological quality, 34% (*n*=10) were rated as moderate, 38% (*n*=11) were rated as low, and 28% (*n*=8) were rated as critically low. Most of the 11 low-quality meta-analyses did not meet the 3 critical items in AMSTAR 2, which were failure to state that the protocol was registered before conducting the meta-analysis, failure to conduct a comprehensive and adequate search, and failure to provide a summary of excluded publications and give reasons for exclusion. In addition to the 3 critical items mentioned above, the 8 critically low-quality meta-analyses largely failed to meet the following 2 critical items: the risk of bias of individual studies was not assessed using appropriate instruments, and the appropriate statistical methods were not used to combine hazard ratios when performing a meta-analysis [36].

### Overlapping and nonoverlapping associations

Seven meta-analyses reported overlapping associations between dietary factors and the incidence of RCC [18, 82–86, 88]. There were 8 associations with varying degrees of overlap. Fifty percent (*n*=4) of the associations identified in the overlapping meta-analyses were consistent in their orientation and magnitude. These associations were with the following factors: dietary fiber [70, 78], cruciferous vegetables [81, 84], vitamin E [74, 85], and coffee [75, 83]. However, 50% (*n*=4) of the directions and magnitudes of the associations were inconsistent, and these associations were with the following factors: red meat [15–18], processed meat [15–18], alcohol [67, 79, 82, 86, 88], and poultry [15, 16]. The details of the meta-analyses of overlapping exposures, which contain the AMSTAR 2 ratings and the decision to retain or exclude each meta-analysis, are presented in Additional file 1: Table S6 [15–18, 70, 74, 75, 78, 79, 81–86, 88, 95]. Detailed information on the overlap of each association assessed with the citation matrix and CCA is shown in Additional file 1: Table S3. A list of the 22 meta-analyses included in this umbrella review and the 7 meta-analyses excluded due to overlap is provided (see Additional file 1: Table S7) [15, 16, 18, 64–88].

### Associations and level of evidence

**Fig. 2:**
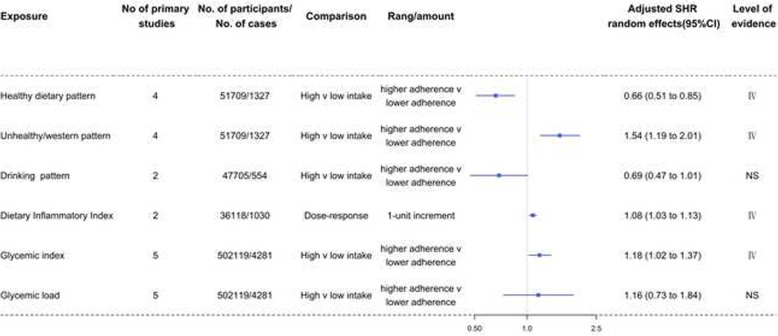
Forest plots and the level of evidence of the association of dietary patterns or dietary quality indices with RCC incidence. SHR summary hazard ratio, NS nonsignificant

**Fig. 3:**
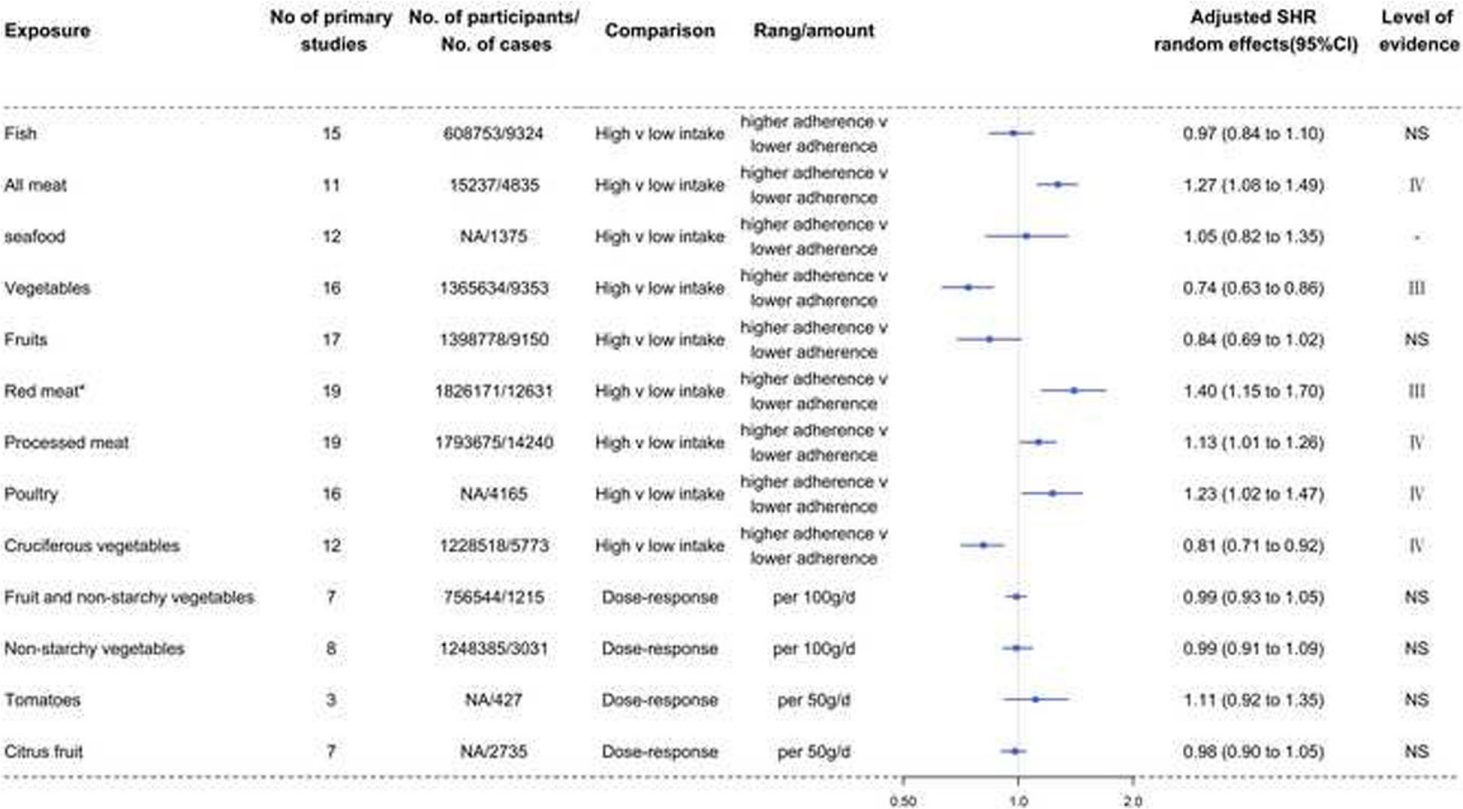
Forest plots and the level of evidence of the association of foods with RCC incidence. SHR summary hazard ratio, NS nonsignificant. *Association was not statistically significant after using the trim-and-fill method. -The level of evidence could not be assessed because data related to 95% PI, *I*^2^, small study effects, and excess significance bias could not be calculated

**Fig. 4:**
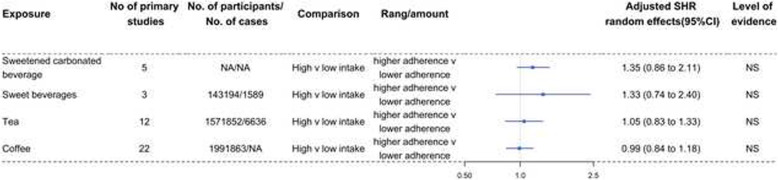
Forest plots and the level of evidence of the association of beverages with RCC incidence. SHR summary hazard ratio, NS nonsignificant

**Fig. 5:**
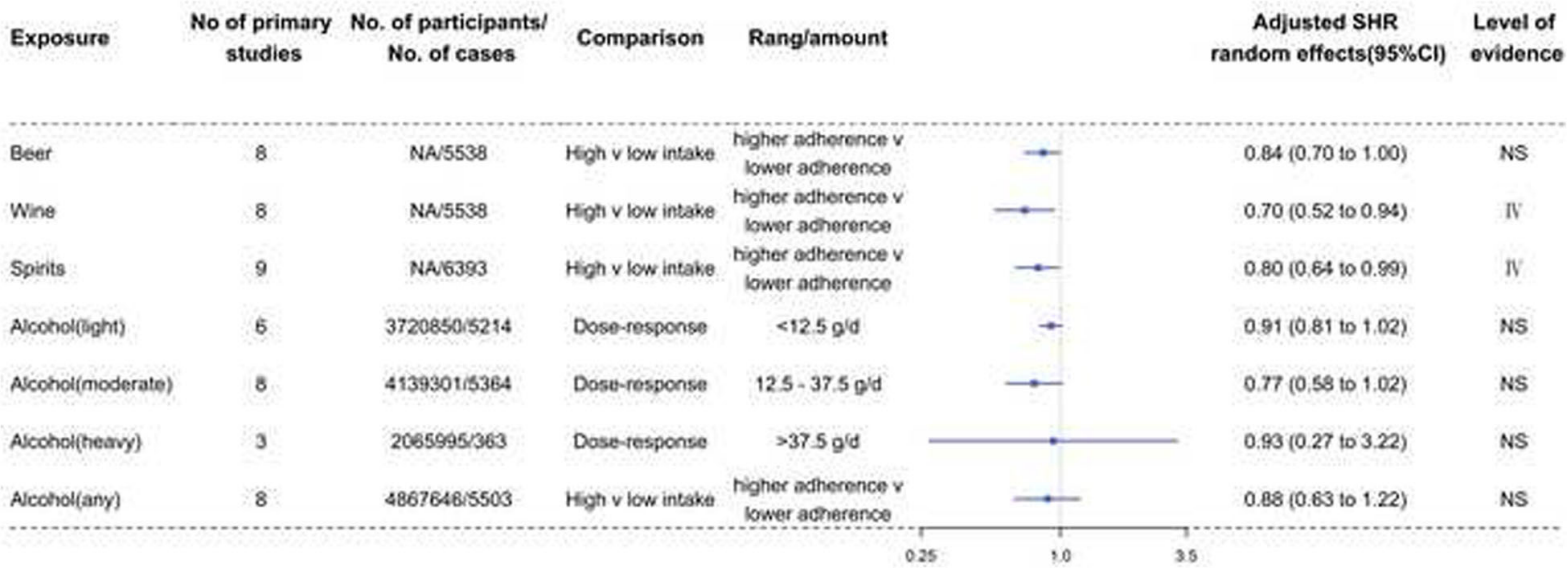
Forest plots and the level of evidence of the association of alcoholic beverages with RCC incidence. SHR summary hazard ratio, NS nonsignificant

**Fig. 6:**
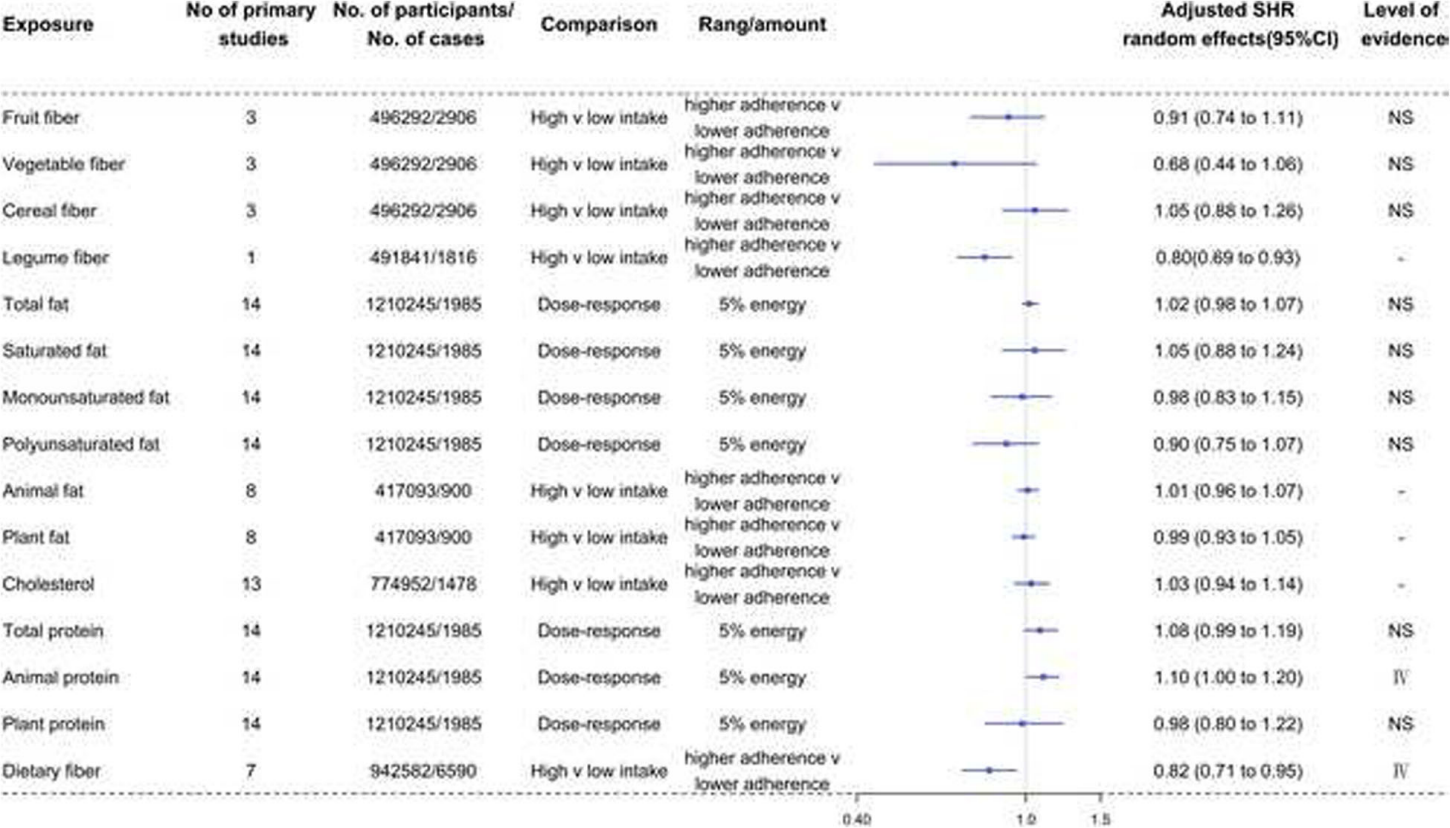
Forest plots and the level of evidence of the association of macronutrients with RCC incidence. SHR summary hazard ratio, NS nonsignificant. -The level of evidence could not be assessed because data related to 95% PI, *I*^2^, small study effects, and excess significance bias could not be calculated

**Fig. 7:**
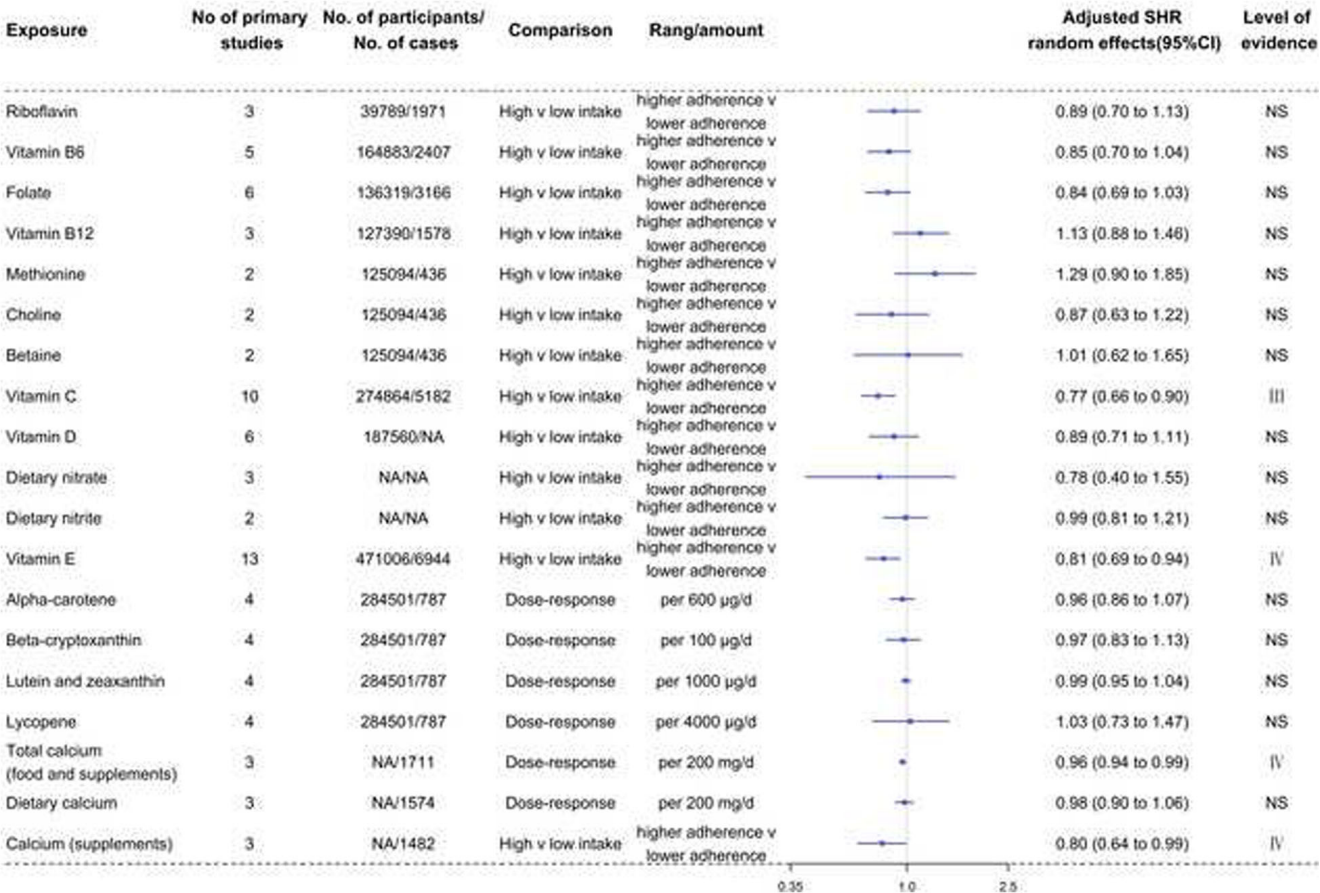
Forest plots and the level of evidence of the association of micronutrients with RCC incidence. SHR summary hazard ratio, NS nonsignificant

The SHR and level of evidence for each dietary factor are summarized in Table 2 and are shown in Figs. 2, 3, 4, 5, 6, and 7. The SHR and 95% CI excluded the null value for 20 of the 64 exposures, including 4 (67%) for dietary patterns or dietary quality indices; 6 (46%) for foods; 0 for beverages; 3 (42%) for alcoholic beverages; 3 (20%) for macronutrients; and 4 (21%) for micronutrients. The SHR was statistically significant (*P*<0.05) for 18 (28%) of the 64 exposures, with 4 (67%) exposures for dietary patterns or dietary quality indices, 6 (46%) for foods with 1 (8%) that were unavailable due to missing data, 0 for beverages, 2 (29%) for alcoholic beverages, 2 (13%) for macronutrients with 4 (27%) that were unavailable due to missing data, and 4 (21%) for micronutrients. Sixty (94%) exposures in the included meta-analyses had more than 1000 cases or 20,000 participants.

**Table 2:** Evidence of associations between diet and renal cell carcinoma

Exposure	Reference	No of primary studies	No. of participants/no. of cases	Adjusted SHR (95%-CI)	***P*** value	***I***^**2**^	95%-PI	Small study effects	Excess significance bias	Largest study significant	Level of evidence
**Dietary patterns or dietary quality indices**											
Healthy dietary pattern	Alizadeh 2017	4	51,709/1327	0.66 (0.51 to 0.85)	0.001449	19.4%	0.27 to 1.63	No	No	No	IV
Unhealthy/Western pattern	Alizadeh 2017	4	51,709/1327	1.54 (1.19 to 2.01)	0.001232	24.70%	0.62 to 3.84	No	No	No	IV
Drinking pattern	Alizadeh 2017	2	47,705/554	0.69 (0.47 to 1.01)	0.057101	0.00%	NA	NA	No	No	NS
Dietary Inflammatory Index	Jayedi 2018	2	36,118/1030	1.08 (1.03 to 1.13)	0.00276	0.00%	NA	NA	No	No	IV
Glycemic index	Xu 2019	5	502,119/4281	1.18 (1.02 to 1.37)	0.026826	26.00%	0.79 to 1.75	No	No	No	IV
Glycemic load	Xu 2019	5	502,119/4281	1.16 (0.73 to 1.84)	0.522525	82.40%	0.21 to 6.45	No	No	No	NS
**Foods**											
Fish	Bai 2013	15	608,753/9324	0.97 (0.84 to 1.10)	0.612245	24.10%	0.63 to 1.47	No	No	No	NS
All meat	Faramawi 2007	11	15,237/4835	1.27 (1.08 to1.49)	0.003529	0.00%	0.83 to 1.94	No	No	No	IV
seafood*	Lee 2008	12	NA/1375	1.05 (0.82 to 1.35)	NA	NA	NA	NA	NA	NA	-
Vegetables	Zhang 2017	16	1,365,634/9353	0.74 (0.63 to 0.86)	0.000073	51.30%	0.44 to 1.23	Yes	No	Yes	III
Fruits	Zhang 2017	17	1,398,778/9150	0.84 (0.69 to 1.02)	0.075352	50.90%	0.39 to 1.78	No	No	Yes	NS
Red meat†	Zhang 2017	19	1,826,171/12631	1.40 (1.15 to 1.70)	0.000807	71.40%	0.63 to 3.13	Yes	No	No	III
Processed meat	Zhang 2017	19	1,793,675/14240	1.13 (1.01 to 1.26)	0.030876	46.80%	0.78 to 1.64	No	No	No	IV
Poultry^#^	Faramawi 2007; Lee 2008	16	NA/4165	1.23 (1.02 to 1.47)	0.030824	0.00%	0.88 to 1.70	No	No	Yes	IV
Cruciferous vegetables	Zhao 2013	12	1,228,518/5773	0.81 (0.71 to 0.92)	0.001107	55.70%	0.54 to 1.20	No	No	Yes	IV
Fruit and non-starchy vegetables	WCRF 2013	7	756,544/1215	0.99 (0.93 to 1.05)	0.748236	0.00%	0.85 to 1.15	Yes	No	No	NS
Non-starchy vegetables	WCRF 2013	8	1,248,385/3031	0.99 (0.91 to 1.09)	0.942029	0.00%	0.80 to 1.24	No	No	No	NS
Tomatoes	WCRF 2013	3	NA/427	1.11 (0.92 to 1.35)	0.28285	0.00%	0.27 to 4.51	No	No	No	NS
Citrus fruit	WCRF 2013	7	NA/2735	0.98 (0.90 to 1.05)	0.53534	0.00%	0.80 to 1.19	No	No	Yes	NS
**Beverages**											
Sweetened carbonated beverage	Boyle 2014	5	NA/NA	1.35 (0.86 to 2.11)	0.187219	60.10%	0.28 to 6.51	No	No	NA	NS
Sweet beverages (include ASB and SSB)	Llaha 2021	3	143,194/1589	1.33 (0.74 to 2.40)	0.336717	85.60%	0.0008 to 2156.90	No	No	No	NS
Tea	Hu 2013	12	1,571,852/6636	1.05 (0.83 to 1.33)	0.680841	71.50%	0.44 to 2.49	No	No	No	NS
Coffee	Wijarnpreecha 2017	22	1,991,863/NA	0.99 (0.84 to 1.18)	0.949021	32.60%	0.50 to 1.98	No	No	No	NS
**Alcoholic beverages**											
Beer	Cheng 2011	8	NA/5538	0.84 (0.70 to 1.00)	0.052184	14.80%	0.52 to 1.34	No	No	No	NS
Wine	Cheng 2011	8	NA/5538	0.70 (0.52 to 0.94)	0.019297	56.00%	0.30 to 1.70	No	No	Yes	IV
Spirits	Cheng 2011	9	NA/6393	0.80 (0.64 to 0.99)	0.048382	74.30%	0.39 to 1.62	No	No	Yes	IV
Alcohol (light)	Xu 2015	6	3,720,850/5214	0.91 (0.81 to 1.02)	0.102662	39.60%	0.64 to 1.28	No	No	No	NS
Alcohol (moderate)	Xu 2015	8	4,139,301/5364	0.77 (0.58 to 1.02)	0.069063	29.20%	0.30 to 1.98	No	No	Yes	NS
Alcohol (heavy)	Xu 2015	3	2,065,995/363	0.93 (0.27 to 3.22)	0.9134	81.20%	0.0000 to 3904180.81	No	Yes	Yes	NS
Alcohol (any)	Xu 2015	8	4,867,646/5503	0.88 (0.63 to 1.22)	0.439292	62.90%	0.27 to 2.81	No	Yes	Yes	NS
**Macronutrients**											
Fruit fiber	Huang 2014	3	496,292/2906	0.91 (0.74 to 1.11)	0.346662	0.00%	0.14 to 6.06	No	No	No	NS
Vegetable fiber	Huang 2014	3	496,292/2906	0.68 (0.44 to 1.06)	0.089923	76.90%	0.003 to 139.60	Yes	No	No	NS
Cereal fiber	Huang 2014	3	496,292/2906	1.05 (0.88 to 1.26)	0.569512	0.00%	0.22 to 4.94	No	No	No	NS
Legume fiber*	Huang 2014	1	491,841/1816	0.80 (0.69 to 0.93)	NA	NA	NA	NA	NA	NA	-
Total fat	WCRF 2013	14	1,210,245/1985	1.02 (0.98 to 1.07)	0.360753	0.00%	NA	NA	No	No	NS
Saturated fat	WCRF 2013	14	1,210,245/1985	1.05 (0.88 to 1.24)	0.60759	41.30%	NA	NA	No	No	NS
Monounsaturated fat	WCRF 2013	14	1,210,245/1985	0.98 (0.83 to 1.15)	0.762472	0.00%	NA	NA	No	No	NS
Polyunsaturated fat	WCRF 2013	14	1,210,245/1985	0.90 (0.75 to 1.07)	0.219824	18.90%	NA	NA	No	No	NS
Animal fat*	Lee 2008	8	417,093/900	1.01 (0.96 to 1.07)	NA	NA	NA	NA	NA	NA	-
Plant fat*	Lee 2008	8	417,093/900	0.99 (0.93 to 1.05)	NA	NA	NA	NA	NA	NA	-
Cholesterol*	Lee 2008	13	774,952/1478	1.03 (0.94 to 1.14)	NA	NA	NA	NA	NA	NA	-
Total protein	WCRF 2013	14	1,210,245/1985	1.08 (0.99 to 1.19)	0.09981	0.00%	NA	NA	No	No	NS
Animal protein	WCRF 2013	14	1,210,245/1985	1.10 (1.00 to 1.20)	0.04936	0.00%	NA	NA	No	No	IV
Plant protein	WCRF 2013	14	1,210,245/1985	0.98 (0.80 to 1.22)	0.88739	0.00%	NA	NA	No	No	NS
Dietary fiber	Xu 2019	7	942,582/6590	0.82 (0.71 to 0.95)	0.007181	27.60%	0.55 to 1.22	No	No	Yes	IV
**Micronutrients**											
Riboflavin	Clasen 2020	3	39,789/1971	0.89 (0.70 to 1.13)	0.343247	0.00%	0.14 to 5.84	No	No	No	NS
Vitamin B6	Clasen 2020	5	164,883/2407	0.85 (0.70 to 1.04)	0.113403	0.00%	0.58 to 1.27	No	No	No	NS
Folate	Clasen 2020	6	136,319/3166	0.84 (0.69 to 1.03)	0.088191	6.00%	0.52 to 1.36	No	No	No	NS
Vitamin B12	Clasen 2020	3	127,390/1578	1.13 (0.88 to 1.46)	0.331795	0.00%	0.19 to 6.65	No	No	No	NS
Methionine	Clasen 2020	2	125,094/436	1.29 (0.90 to 1.85)	0.170519	0.00%	NA	NA	No	No	NS
Choline	Clasen 2020	2	125,094/436	0.87 (0.63 to 1.22)	0.433326	0.00%	NA	NA	No	No	NS
Betaine	Clasen 2020	2	125,094/436	1.01 (0.62 to 1.65)	0.962844	58.80%	NA	NA	No	No	NS
Vitamin C	Jia 2015	10	274,864/5182	0.77 (0.66 to 0.90)	0.000636	0.00%	0.54 to 1.10	No	No	No	III
Vitamin D	Wu 2020	6	187,560/NA	0.89 (0.71 to 1.11)	0.286865	36.50%	0.48 to 1.64	No	No	No	NS
Dietary nitrate	Xie 2016	3	NA/NA	0.78 (0.40 to 1.55)	0.482996	89.20%	0.0002 to 3013.70	No	No	NA	NS
Dietary nitrite	Xie 2016	2	NA/NA	0.99 (0.81 to 1.21)	0.892113	0.00%	NA	NA	No	No	NS
Vitamin E	Shen 2015	13	471,006/6944	0.81 (0.69 to 0.94)	0.006361	49.20%	0.52 to 1.26	No	No	No	IV
Alpha-carotene	WCRF 2013	4	284,501/787	0.96 (0.86 to 1.07)	0.448428	33.10%	0.32 to 2.86	No	No	No	NS
Beta-cryptoxanthin	WCRF 2013	4	284,501/787	0.97 (0.83 to 1.13)	0.700635	59.10%	0.19 to 4.91	No	No	Yes	NS
Lutein and zeaxanthin	WCRF 2013	4	284,501/787	0.99 (0.95 to 1.04)	0.827912	0.00%	0.71 to 1.40	Yes	No	No	NS
Lycopene	WCRF 2013	4	284,501/787	1.03 (0.73 to 1.47)	0.849859	5.90%	0.04 to 30.00	No	No	No	NS
Total calcium (food and supplements)	WCRF 2013	3	NA/1711	0.96 (0.94 to 0.99)	0.017337	0.00%	0.78 to 1.19	No	No	Yes	IV
Dietary calcium	WCRF 2013	3	NA/1574	0.98 (0.90 to 1.06)	0.614121	0.00%	0.45 to 2.12	No	No	No	NS
Calcium (supplements)	WCRF 2013	3	NA/1482	0.80 (0.64 to 0.99)	0.039569	0.00%	NA	NA	No	No	IV

^*^SHR derived from published meta-analysis, no recalculation; ^†^The association was not statistically significant after using the trim-and-fill method; ^#^SHR was generated by combining the hazard ratios from the 2 meta-analyses; *SHR* summary hazard ratio; *NA* not available; *NS* nonsignificant; *CI* confidence intervals; *PI* prediction intervals; *ASB* artificially sweetened beverages; *SSB* sugar-sweetened beverages

### Dietary patterns or dietary quality indices

Table 2 and Fig. 2 show the SHR, 95% CI, and level of evidence associating dietary patterns or dietary quality indices with the incidence of RCC. No dietary factors showed convincing or highly suggestive evidence of an association with RCC. There was 1 instance of weak evidence (class IV) that long-term adherence to a healthy dietary pattern 0.66 (0.51 to 0.85) was associated with a lower risk of developing RCC compared with nonadherence to a healthy dietary pattern. Similarly, there were 3 weak pieces of evidence (class IV) that chronic exposure to an unhealthy/Western pattern (1.54, 1.19 to 2.01), a high dietary inflammatory index (1.08, 1.03 to 1.13), or a hyperglycemic index (1.18, 1.02 to 1.37) was associated with a higher risk of developing RCC. Drinking pattern and glycemic load were not significantly associated with the incidence of RCC.

### Foods, beverages, and alcoholic beverages

Table 2 and Fig. 3 show the SHR, 95% CI, and level of evidence for the association of foods with the incidence of RCC. No exposures for RCC showed convincing or highly suggestive evidence of an association. There was 1 case of suggestive evidence (class III) of an inverse association between increased vegetable (0.74, 0.63 to 0.86) intake and the incidence of RCC. There were 3 pieces of weak evidence (class IV) that increased intake of all meats (1.27, 1.08 to 1.49), processed meat (1.13, 1.01 to 1.26), and poultry (1.23, 1.02 to 1.47) were positively associated with the incidence of RCC. There was 1 instance of weak evidence (class IV) that increased intake of cruciferous vegetables (0.81, 0.71 to 0.92) were negatively associated with the incidence of RCC. Fish, fruits, fruit and non-starchy vegetables, non-starchy vegetables, tomatoes, and citrus fruit intake were not associated with the incidence of RCC. Seafood intake was not associated with RCC incidence. However, the level of evidence could not be assessed because data related to 95% PI, *I*^2^, small study effects, and excess significance bias could not be calculated.

Table 2 and Fig. 4 show the SHR, 95% CI, and level of evidence for the association of beverages with the incidence of RCC. No exposures for RCC showed convincing or highly suggestive evidence of an association. The intake of sweetened carbonated beverage, sweetened beverages, tea, and coffee was not associated with the incidence of RCC.

Table 2 and Fig. 5 show the SHR, 95% CI, and level of evidence for the association of alcoholic beverages with the incidence of RCC. No exposures for RCC showed convincing or highly suggestive evidence of an association. There were 2 pieces of weak evidence (class IV) that increased intake of wine (0.70, 0.52 to 0.94) and spirits (0.80, 0.64 to 0.99) was inversely associated with the incidence of RCC. Beer intake was not significantly associated with the incidence of RCC. Dose-response meta-analyses showed that light drinking (<12.5 g/d), moderate drinking (12.5–37.5 g/d), and heavy drinking (>37.5 g/d) were not significantly associated with the incidence of RCC. A high vs low meta-analysis also showed that any drinking was not significantly associated with the risk of developing RCC.

### Macronutrients and micronutrients

Table 2 and Fig. 6 show the SHR, 95% CI, and level of evidence of the association of macronutrients with RCC incidence. No exposures for RCC showed convincing or highly suggestive evidence of an association. There was only 2 instance of weak evidence (class IV) showing that an inverse association between increased dietary fiber (0.82, 0.71 to 0.95) intake and the incidence of RCC and that increased animal protein (1.10, 1.00 to 1.20) intake was associated with the risk of RCC. Fruit fiber, vegetable fiber, cereal fiber, total fat, saturated fat, monounsaturated fat, polyunsaturated fat, total protein, and plant protein were not significantly associated with the incidence of RCC. The remaining dietary factors (legume fiber, animal fat, plant fat, and cholesterol) could not be assessed for their level of evidence because the data related to 95% PI, *I*^2^, small study effects, and excess significance bias could not be calculated.

Table 2 and Fig. 7 show the SHR, 95% CI, and level of evidence of the association of micronutrients with the incidence of RCC. No exposures showed convincing or highly suggestive evidence of an association with RCC. There was 1 instance of suggestive evidence (class III) that increased intake of vitamin C (0.77, 0.66 to 0.90) was associated with a lower risk of RCC. There were 3 pieces of weak evidence (class IV) that increased intake of vitamin E (0.81, 0.69 to 0.94), total calcium from food and supplements (0.96, 0.94 to 0.99), and calcium from supplements (0.80, 0.64 to 0.99) was negatively related to the risk of RCC. The remaining dietary factors (riboflavin, folate, vitamin B12, methionine, betaine, vitamin D, dietary nitrate, choline, dietary nitrite, vitamin B6, alpha-carotene, beta-cryptoxanthin, lutein and zeaxanthin, lycopene, and dietary calcium) were not linked to the development of RCC.

### Heterogeneity

The *I*^2^, *τ*^2^, and 95% PI are described in Additional file 1: Table S8 [15–17, 64–81, 87]. Of the 64 exposures, 8% (*n*=5) of *I*^2^, 8% (*n*=5) of *τ*^2^ and 30% (*n*=19) of 95% PI values could not be recalculated. In total, 67% (*n*=43) did not have high heterogeneity (*I*^2^<50%) for the following factors: healthy dietary pattern, unhealthy/Western pattern, drinking pattern, dietary inflammatory index, glycemic index, fish, all meat, processed meat, poultry, fruit and non-starchy vegetables, non-starchy vegetables, tomatoes, citrus fruit, coffee, beer, alcohol consumption (light), alcohol consumption (moderate), fruit fiber, cereal fiber, total fat, saturated fat, monounsaturated fat, polyunsaturated fat, total protein, animal protein, plant protein, dietary fiber, riboflavin, vitamin B6, folate, vitamin B12, methionine, choline, vitamin C, vitamin D, dietary nitrite, vitamin E, alpha-carotene, lutein and zeaxanthin, dietary lycopene, total calcium (food and supplements), dietary calcium, and calcium (supplements). For 95% PI, all exposures included null values.

### Publication bias and small study effects

Of the 59 contour-enhanced funnel plots, 29% (*n*=17) included 10 or more individual studies, 31% (*n*=18) included between 5 and 10 individual studies, and 31% (*n*=18) included fewer than 3 individual studies. The contour-enhanced funnel plots corresponding to each exposure are shown (see Additional file 2: Figs. S1–S10). Small study effects for five dietary factors, namely, vegetables, red meat, fruit and non-starchy vegetables, vegetable fiber, and lutein and zeaxanthin (Egger’s test; *p*=0.049, *p*=0.003, *p*=0.079, *p*=0.096, and *p*=0.093, respectively) were shown in Additional file 1: Table S8 and Additional file 2: Figs. S2B, S2D, S3C, S6B, and S10C. Using the trim-and-fill technique, we found that for vegetables, 2 missing studies were added, and the association (0.77, 0.67 to 0.88) was weaker than the previous association (0.74, 0.63 to 0.86) (see Additional file 2: Fig. S11A); for red meat, 8 missing studies were added, and the association (1.10, 0.93 to 1.30) was not statistically significant as was the previous association (1.40, 1.15 to 1.70) (see Additional file 2: Fig. S11B); for vegetable fiber and fruit and non-starchy vegetables, no missing studies were added to the plot (see Additional file 2: Fig. S11C and S11D); for lutein and zeaxanthin, 2 missing studies were added, and they are still not significantly associated with the risk of RCC (see Additional file 2: Fig. S11E).

### Subgroup analysis and sensitivity analysis

The results of the subgroup analyses of the associations between various dietary factors and the risk of RCC by study design are shown in Additional file 1: Table S9. In full analysis, increased intake of vegetables (class III) and dietary fiber (class IV) was significantly associated with a lower risk of RCC and was also observed to be significantly associated in both cohort and case-control studies. In the case-control study, the evidence level for vegetables increased from class III to class II due to a decrease in *p* value from 7.3×10^-5^ to 9.18×10^-10^ and the evidence level for dietary fiber also increased from class IV to class III due to a decrease in *p* value from 7.18×10^-3^ to 4×10^-4^. Nine instances of evidence with a statistically significant difference were observed for nonsignificant results in the cohort study, but significant results in the case-control study (healthy dietary pattern, unhealthy/Western pattern, dietary inflammatory index, glycemic index, red meat, poultry, cruciferous vegetables, vitamin C, vitamin E). For processed meat, a weak level of evidence in full analysis was found, whereas the results were nonsignificant in both cohort and case-control, respectively.

The results of the subgroup analyses of the associations between various dietary factors and the risk of RCC by region are shown in Additional file 1: Table S10. Long-term adherence to a healthy dietary pattern was significantly associated with a low risk of RCC in America, but not in Europe (class III, 0.62, 0.51 to 0.76). Similarly, long-term adherence to an unhealthy dietary pattern (class III, 1.61, 1.33 to 1.95) and increased intake of red meat (class III, 1.61, 1.24 to 2.10) and processed meat (class III, 1.19, 1.11 to 1.28) were significantly associated with a high risk of RCC in America, but not in Europe and Asia. Increased intake of all meat (class III, 1.27, 1.13 to 1.43) was significantly associated with a high risk of RCC in North America, however, increased intake of cruciferous vegetables (class II, 0.78, 0.70 to 0.86) and dietary fiber (class III, 0.75, 0.67 to 0.85) was significantly associated with a low risk of RCC. Increased intake of any alcohol (class III, 0.89, 0.83 to 0.94) and moderate drinking (class I, 0.77, 0.70 to 0.84) were significantly associated with a low risk of developing RCC in Europe and North America. Increased vegetable (class III, 0.70, 0.60 to 0.81) intake is significantly associated with a lower risk of RCC in Europe.

Only vegetables maintained the same level of evidence after removing small-sized studies and low-quality studies (see Additional file 1: Table S11). After removing the small-sized studies, the level of evidence for vitamin C decreased from suggestive evidence (class III) to weak evidence (class IV) due to an increase in *p* value from 6.63×10^-4^ to 9.71×10^-3^.

## Discussion

### Main findings

We conducted an umbrella review of the effect of diet, including dietary patterns, dietary quality indices, foods, beverages (including alcohol), and nutrients, rather than a specific dietary factor, on the incidence of RCC. In our study, the evidence associating dietary factors with the incidence of RCC from available meta-analyses was summarized, and the strength and validity of this evidence were evaluated.

There were 22 published meta-analyses included, with a total of 64 SHRs for various dietary factors and RCC risk. No meta-analyses were rated as high in terms of methodological quality, and approximately 1/3 were rated as moderate. Two pieces of suggestive evidence (class III) suggested that increasing vegetable and vitamin C intake might have a negative correlation with the incidence of RCC. Eight pieces of weak evidence (class IV) showed that adhering to a healthy dietary pattern was associated with a lower risk of RCC and that increased intake of cruciferous vegetables, wine, spirits, dietary fiber, vitamin E, total calcium (foods and supplements), and calcium (supplements) were negatively associated with the incidence of RCC. Seven pieces of weak evidence (class IV) showed that long-termed unhealthy pattern is positively linked with RCC incidence and that a 1-unit increase in the dietary inflammatory index, hyperglycemic index, and increased intake of all meat, processed meat, poultry, and animal protein was associated with a higher risk of RCC. The remaining dietary factors were not associated with RCC incidence.

In subgroup analysis on regions, a piece of convincing evidence (class I) suggested that moderate drinking had a positive association with a low risk of developing RCC in Europe and North America. A piece of suggestive evidence (class III) suggested that increased consumption of any alcohol was positively related to a low risk of developing RCC in Europe and North America. A piece of highly suggestive evidence (class II) suggested that increased intake of cruciferous vegetables was positively linked with a low risk of RCC in North America. Two pieces of suggestive evidence (class III) suggested that increased consumption of all meat was positively related to a high risk of RCC and that increased consumption of dietary fiber was positively linked with a low risk of RCC in North America. Four pieces of suggestive evidence (class III) suggested that a healthy dietary pattern might have a negative correlation with the incidence of RCC and that an unhealthy dietary pattern and increased intake of red meat and processed meat were positively associated with a high risk of RCC in America. Two pieces of suggestive evidence (class III) suggested that increased intake of vegetables and Vitamin C is significantly associated with a lower risk of RCC in Europe.

### Possible explanations and comparison with other studies

Increasing vegetable consumption was found to be inversely related to RCC risk in our study. A meta-analysis conducted by Zhang et al. found that the protective effect of vegetables was diminished after adjustment for body mass index [80], implying that obesity might be an important intermediate mechanism between vegetable consumption and the incidence of RCC. Obesity has been acknowledged by the guidelines of the European Association of Urology as a risk factor for RCC [146], which might be related to the inverse association between vegetables and obesity [147–151]. A randomized double-blind controlled trial in Japan showed that increasing the consumption of vegetables rich in carotenoids reduced visceral obesity in Japanese men [151]. Dreher et al. conducted a randomized controlled trial and prospective trial on the effect of vegetable consumption on weight loss in females and concluded that increased vegetable intake was a major factor in weight loss in women [150]. Moreover, a diet low in vegetables contributes to obesity and a pro-inflammatory adipokine profile [149]. A healthy pattern consisting of vegetables, fruits, juices, and exercise was found to be inversely associated with abdominal obesity [148].

Phytochemical extracts and compounds secreted from vegetables, such as isothiocyanates (ITC), sulforaphane (SFN), and indole-3-carbinol (I3C) [152], have strong antioxidant and antiproliferative activity [153, 154] and may be the main anticancer components [155]. Cytochrome P450 (CYP) enzymes are biotransformation enzymes whose reduced activity inhibits cancer progression [156]. SFN can inhibit the activity of CYP3A4 in human hepatocytes [157]. In cultured human cells, ITC was observed to have potent antiproliferative activity [158–161]. In addition, ITC, indole, and vegetable-secreted compounds play an important role in blocking carcinogen-activating enzymes, triggering carcinogen-detoxifying enzymes, increasing apoptosis, and halting cell cycle progression [152, 154, 157, 162, 163]. Notably, there is potential for publication bias in the meta-analysis associating vegetable consumption with RCC incidence in this study. The association was found to be attenuated after using the trim-and-fill technique, implying that the results of this meta-analysis were exaggerated. However, the association remained statistically significant after imputing 2 additional studies in the plot by using the trim-and-fill method. To verify the robustness of the results, we performed sensitivity analyses by excluding small-sized studies and low-quality studies. Sensitivity analyses indicated that no significant variation in adjusted SHR and the level of evidence remains at class III, confirming the stability of present results.

Our study found that increased vitamin C intake was negatively associated with the incidence of RCC. Extensive cytosine methylation abnormalities are associated with RCC tumorigenesis, and 5-hydroxymethylcytosine (5hmC) deficiency is associated with the recurrence of RCC [164]. 5hmC deficiency can decrease the survival of RCC patients [165]. 5-methylcytosine (5mC) can be transformed to 5hmC with the intervention of ten-eleven translocation (TET) enzymes [166]. However, TET enzymes are frequently mutated [167]. A decrease in TET enzyme activity would lead to a decrease in 5hmC levels and thus potentially carcinogenesis [168–171]. A decrease in TET enzyme activity might be associated with a deficiency of the cofactor (vitamin C) of the TET gene. In addition, vitamin C, an important antioxidant, is commonly used to reduce carcinoma risk [72]. Experimental studies have revealed that 8-hydroxy-2'-deoxyguanosine and 4-hydroxy-2-nonenal-modified proteins play important oxidative roles in human RCC [172, 173]. However, vitamin C may contribute significantly to the antioxidant effect and increase 5hmC levels. Notably, the sensitivity analysis suggested vitamin C downgraded to a weak (class IV) level of evidence by excluding small-sized studies. Therefore, we assume that there might be an exaggerated relationship between vitamin C and a low risk of developing RCC.

Our study confirmed a false association between red meat intake and the risk of RCC [17]. The SHR (1.40,1.15 to 1.70) for red meat intake and the incidence of RCC was obtained by combining hazard ratios from 19 individual studies, implying that an increased ingestion of red meat was significantly correlated with RCC risk. Based on the p value for Egger’s test (*p*=0.003) and asymmetric contour-enhanced funnel plots (see Additional file 1: Table S8 and Additional file 2: Fig. S2D), we found that 8 missing studies were added to the plot for red meat by using the trim-and-fill technique, and the association (1.10, 0.93 to 1.30) was no longer statistically significant. Moreover, the association initially rated as class III was no longer considered suggestive evidence and was downgraded to class V (nonsignificant). However, heterogeneity between regions or populations is reasonable to suspect because of different dietary patterns across populations. So we performed subgroup analysis by regions. We found that healthy dietary pattern was inversely associated with the risk of RCC and unhealthy/Western dietary pattern had a positive correlation with RCC risk in America, but not in Europe or Asia. This unhealthy/Western dietary pattern is often characterized by a high intake of red meat and processed meat. A prospective trial based on a large USA cohort identifies cooking compounds, particularly benzo(a)pyrene (a polycyclic aromatic hydrocarbon) and 2-amino-1-methyl-6-phenyl-imidazo[4,5-b]pyridine (a heterocyclic amines), as a possible mechanism for the association of increased red meat intake with high risk of RCC [174]. The healthy dietary pattern is characterized by a higher intake of vegetables and dietary fiber, especially cruciferous vegetables. Cruciferous vegetables are rich in ITC, SFN, and I3C [152], which may have strong antioxidant [153, 154] and anticancer effects [155].

Our study found 2 pieces of convincing and suggestive evidence that moderate drinking (class I) and any drinking (class III) is more likely to be associated with a lower risk of developing RCC in Europeans and Americans compared to Asians, and a nonsignificant result that heavy drinking is not associated with RCC risk, which is consistent with what Bellocco and colleagues found [82]. However, they reported a negative association between light alcohol consumption and the risk of RCC, which is inconsistent with our results. The inclusion of different individual studies might be one of the reasons for the inconsistency. The Bellocco meta-analysis included case-control studies and cohort studies on the association between alcohol drinking and RCC risk, whereas we included only prospective cohort studies. Furthermore, their meta-analysis did not include these three new individual studies associating alcohol drinking with RCC [175–177]. In addition, differences in the extent to which people in different regions and races are affected by alcohol may also contribute to the inconsistency.

We found a total of 18 dietary factors associated with the risk of RCC. Some results must be interpreted with caution. In case of processed meat, evidence was significant in the full analysis, whereas the results were nonsignificant in both cohort and case-control, respectively. Different study designs and the heterogeneity between studies may be one of the reasons to explain this result. To conclude, because the outcomes were nonsignificant in both study designs, the evidence of processed meat could overestimate the true effect and could thus be reconsidered. Moreover, we found that cohort studies tended to show nonsignificant results, while case-control studies tended to show significant results and higher levels of evidence, such as the 9 associations between dietary factors with RCC risk (healthy dietary pattern, unhealthy/Western pattern, dietary inflammatory index, glycemic index, red meat, poultry, cruciferous vegetables, vitamin C, vitamin E). So meta-analysis of case-control studies may have spurious associations or even seriously drive the magnitudes of the associations.

There has been much interest in the topic of preventing noncommunicable diseases (e.g., cancer) by modifying dietary habits or patterns. To date, a large number of publications have reported correlations between diet and RCC incidence, including individual studies and SRoMAs, providing a large body of evidence. However, most current guidelines, including those of the European Society for Medical Oncology, the American Urological Association, and the National Comprehensive Cancer Network, do not provide recommendations on how to prevent the incidence of RCC by modifying dietary habits or patterns. Moreover, the European Association of Urology guidelines only briefly mention that specific dietary habits may influence the incidence of RCC, but the findings were inconclusive [146]. Although the highest level of evidence derived from the present umbrella review was suggestive evidence (class III), it is the best evidence available and fills a gap in the guidelines. In our review, all presently available evidence from SRoMAs on dietary factors and the incidence of RCC was synthesized, and the level of evidence was appraised. From the results of the study, it is clear that increased ingestion of vegetables and vitamin C has a negative association with the incidence of RCC. Although the 2 pieces of evidence were suggestive (class III), both exposures (vegetables and vitamin C) were associated with RCC incidence (*p*<10 ^−3^), there was no high heterogeneity between studies, and there was no excess significance bias. Notably, both 95% PIs for exposures included null values, implying that in some cases, the effect of exposure on outcome may be nonexistent. As a result, the findings should be interpreted with caution. In addition, our study supports the statement that moderate alcohol consumption is protective, as mentioned in the European Association of Urology guidelines [146], but only for Europeans and North Americans.

In a previous study, Papadimitriou and colleagues performed an umbrella review of evidence on the association of diet with the risk of 11 cancers (including RCC) [178]. The present study differs from their study in the following ways: (1) their literature was searched through March 2013, whereas ours was searched through April 2021; (2) the databases searched were not identical; (3) the present study included more recent meta-analyses and included more exposures (64 v 24), such as dietary inflammatory index, glycemic index, vitamin D, and riboflavin; (4) their study did not exclude overlapping SRoMAs and therefore, was likely to include duplicate individual studies, resulting in biased results and associations [46, 47]. In contrast, our study used a validated CCA tool to evaluate the degree of overlap of overlapping meta-analyses and selected the highest quality meta-analyses available; (5) their study included 274 individual studies without excluding overlapping meta-analyses, while our study included 502 individual studies after excluding overlapping meta-analyses; (6) their study did not use relevant quality appraisal instruments to evaluate the methodological quality of the included SRoMAs, whereas our study used AMSTAR 2 to address this issue; and (7) our study included pooled studies in addition to cohort studies and case-control studies. Thus, our study differs from the previous study in many ways but is similar in terms of the section on dietary fiber.

### Strengths and limitations

The present study had many strengths. Our study synthesized evidence from the existing available published meta-analyses. The AMSTAR 2 instrument was employed to assess the methodological quality of the included meta-analyses. We employed credibility assessment criteria by conducting a large number of statistical tests to classify the level of evidence, as in previous umbrella reviews [23, 24, 27, 29, 30]. CCA combined with AMSTAR 2 was used to quantify the degree of overlap of overlapping meta-analyses and to select the highest quality and most recent meta-analysis, thus avoiding double counting and selection bias. We used not only contour-enhanced funnel plots but also a combination of Egger’s regression asymmetry tests and the trim-and-fill technique to determine whether the asymmetry of the plots was precipitated by publication bias and to assess whether the included meta-analyses reported exaggerated results.

Some limitations exist in the present study. Missing data in the included meta-analyses prevented us from calculating some metrics, such as small study effects, *I*^2^, *τ*^2^, 95% PI, and excess significance bias. Therefore, the level of this part of the evidence could not be evaluated. In addition, we were unable to assess whether there was publication bias in this meta-analysis because some of the meta-analyses included too few individual studies, which prevented us from performing Egger’s test [179]. During our literature screening process, we found some reviews associating some dietary factors with RCC. However, our umbrella review did not include these publications because they were not SRoMAs (i.e., narrative reviews or synthesis of reviews). Therefore, the present umbrella review did not include a small proportion of the evidence associating a particular dietary exposure with RCC incidence. For example, Jeyaraman et al. found no significant association between dairy intake and the incidence of RCC [103], whereas a narrative review by Key et al. reported that increased dairy ingestion increased the RCC risk [106]. Limited data availability made it impossible to assess the exact association between dairy products and the incidence of RCC. We did not include systematic reviews without meta-analyses, which may lead to bias. This may limit the generalizability of our results if the systematic reviews without meta-analysis or non-SRoMAs contain issues that were not included in the umbrella review [90, 93, 104–106, 110, 125, 134, 180]. In addition, from the search date to the publication of the results of this study, new SRoMAs showing an association of some dietary factors with RCC may have been published online.

Fundamentally, since the data in our study come from observational studies, confounding factors are inevitable [181]. Recall bias and selection bias are more prominent in case-control studies than in cohort studies because lifestyle and dietary factors are identified following a cancer diagnosis in case-control studies. In addition, some of the individual studies might have unknown confounding factors. The meta-analyses included in this umbrella review, however, were corrected as much as possible for confounding factors such as sex, age, body mass index, and smoking. More prospective research, such as cohort studies or randomized controlled trials, is needed in the future because residual confounding factors could not be totally ruled out.

Due to the rigorous items of the AMSTAR 2 instrument, the methodological quality of many of the meta-analyses was graded as low or critically low. However, inadequate reporting of meta-analyses rather than methodological flaws can also lead to a downgrading of methodological quality. Since our study included SRoMAs, the quality of the individual studies was not assessed. This should be the work of the investigators of the SRoMAs. Gray literature was not considered in this study, which may lead to some bias. Umbrella review evidence is also subject to residual confounding factors due to the type of original studies included, which were mostly observational in nature. The evidence associating legume fiber with RCC came from only one individual study [70], and the number of reported events was low, which might lead to inaccurate results.

Heterogeneity in this study may arise from the study design, region, control selection, sex, and measurement methods. For example, the meta-analyses included in this study included case-control studies, cohort studies, and pooled studies. In addition, heterogeneity in this study may stem from the definitions of different comparator groups, as different studies set specific comparator groups, such as tertiles and quartiles of vegetable intake levels.

Regarding publication bias, according to Egger’s test and contour-enhanced funnel plots, only 5 exposures in this study were potentially subject to publication bias, but 31% of the meta-analyses contained 5 to 10 individual studies, and 31% included no more than 3 individual studies (see Additional file 2: Figs. S1–S10), which implies that the results may be unreliable. Therefore, more studies of the associations of dietary factors with RCC risk are needed in the future. For example, using the trim-and-fill method, our study found that 2 additional studies on vegetable intake are needed on the right-hand side, and 8 additional studies on red meat intake are needed on the left-hand side. This is especially true for other meta-analyses that included even smaller numbers of individual studies.

### Future research outlook

To obtain more high-quality evidence on dietary factors and the risk of RCC, future studies should consider the following aspects. To reduce bias and confounding factors, studies with high-quality study designs, such as prospective cohort studies or randomized controlled trials, are recommended. Efforts are being made to conduct more research on the biological mechanisms associating specific dietary factors with RCC risk. Guidelines should be followed for conducting SRoMAs to improve methodological quality and to obtain high-quality evidence, such as identifying the protocol before performing the systematic review, registering the protocol on a website (e.g., PROSPERO), conducting a comprehensive search (including unpublished data and gray literature), providing a summary of excluded publications, giving reasons for exclusion, and selecting an appropriate quality assessment instrument to assess the risk of bias for each included individual study. Considering the potential for food interactions and the effect of confounding factors, it is recommended that studies on the effect of different dietary patterns on the incidence of RCC be conducted in the context of specific scenarios whenever possible.

## Conclusions

Overall, although a large number of SRoMAs have been published on the associations of diet with RCC incidence, none of the evidence has been classified as classes I–II in overall analysis. Increased intake of vegetables and vitamin C is inversely associated with the incidence of RCC, but the level of the evidence is class III. Moderate drinking may be beneficial for Europeans and North Americans, and cruciferous vegetables may be beneficial to North Americans. More research is needed in the future.

## Supplementary Information

**Additional file 1: Table S1.** Embase Search Strategy. **Table S2.** Data extraction form for included systematic reviews and individual studies. **Table S3.** Citation matrices for meta-analyses with overlapping associations. **Table S4.** List of excluded studies and reasons for their exclusion. **Table S5.** AMSTAR 2 quality appraisal scores. **Table S6.** General characteristics of meta-analyses with overlapping associations. **Table S7.** A. List of studies included in analysis B. List of meta-analyses with overlapping associations excluded from analysis. **Table S8.** Characteristics of the conducted meta-analyses and results of the recalculation, the methodological assessment (AMSTAR 2), and the level of evidence by exposure. **Table S9.** Summary of results of the associations on various dietary factors and risk of RCC by study design. **Table S10.** Summary of results of the associations on various dietary factors and risk of RCC by region. **Table S11.** Sensitivity analysis and the level of evidence of the association of vegetables and vitamin C with RCC.

**Additional file 2: Figure S1.** Contour-enhanced funnel plots associating dietary patterns or diet quality indices with RCC incidence: A) healthy dietary pattern, B) unhealthy/Western pattern, C) drinking pattern, D) dietary inflammatory index, E) glycemic index, and F) glycemic load. **Figure S2.** Contour-enhanced funnel plots associating foods with RCC incidence: A) fish, B) vegetables, C) fruits, D) red meat, E) processed meat, and F) cruciferous vegetables. **Figure S3.** Contour-enhanced funnel plots associating foods with RCC incidence: A) poultry, B) all meat, C) fruit and non-starchy vegetables, D) non-starchy vegetables, E) tomatoes, and F) citrus fruit. **Figure S4.** Contour-enhanced funnel plots associating beverages with RCC incidence: A) sweet beverages (including artificially sweetened beverages and sugar-sweetened beverages), B) tea, C) coffee, and D) sweetened carbonated beverage. **Figure S5.** Contour-enhanced funnel plots associating alcoholic beverages with RCC incidence: A) beer, B) wine, C) spirits, D) alcohol (light), E) alcohol (moderate), F) alcohol (heavy), and G) alcohol (any). **Figure S6.** Contour-enhanced funnel plots associating macronutrients with RCC incidence: A) fruit fiber, B) vegetable fiber, C) cereal fiber, D) dietary fiber, E) Total fat, and F) saturated fat. **Figure S7.** Contour-enhanced funnel plots associating macronutrients with RCC incidence: A) monounsaturated fat, B) polyunsaturated fat, C) total protein, D) animal protein, and E) plant protein. **Figure S8.** Contour-enhanced funnel plots associating micronutrients with RCC incidence: A) riboflavin, B) vitamin B6, C) folate, D) vitamin B12, E) methionine, and F) choline. **Figure S9.** Contour-enhanced funnel plots associating micronutrients with RCC incidence: A) betaine, B) vitamin C, C) vitamin D, D) dietary nitrate, E) dietary nitrite, and F) vitamin E. **Figure S10.** Contour-enhanced funnel plots associating micronutrients with RCC incidence: A) alpha-carotene, B) beta-cryptoxanthin, C) lutein and zeaxanthin, D) dietary lycopene, E) total calcium (food and supplements), F) dietary calcium, and G) Calcium (supplements). **Figure S11.** After using the trim-and-fill method, contour-enhanced funnel plots for the association between A) vegetables, B) red meat, C) vegetable fiber, D) fruit and non-starchy vegetables, and E) lutein and zeaxanthin and the incidence of renal cell carcinoma were generated.

## Data Availability

Data were extracted from published meta-analyses.

## Abbreviations

AMSTAR: A MeaSurement Tool to Assess systematic Reviews
SRoMAs: Systematic reviews or meta-analyses
RCC: Renal cell carcinoma
CI: Confidence intervals
CCA: Corrected covered area
PI: Prediction intervals
SHR: Summary hazard ratios
O: The observed number
E: The expected number
NOS: The Newcastle-Ottawa Scale
ITC: Isothiocyanates
SFN: Sulforaphane
CYP: Cytochrome P450
TET: Ten eleven translocation
5mC: 5-Methylcytosine
5hmC: 5-Hydroxymethylcytosine
