# Supplementary material for: The role of diet in renal cell carcinoma incidence: an umbrella review of meta-analyses of observational studies
Source: BMC Med. 2022 Feb 3;20:39. doi: 10.1186/s12916-021-02229-5 (PMC8812002; doi:10.1186/s12916-021-02229-5)
Supplement: Supplementary file 1 — Additional file 1: Table S1. Embase Search Strategy. Table S2. Data extraction form for included systematic reviews and individual studies. Table S3. Citation matrices for meta-analyses with overlapping associations. Table S4. List of excluded studies and reasons for their exclusion. Table S5. AMSTAR 2 quality appraisal scores. Table S6. General characteristics of meta-analyses with overlapping associations. Table S7. A. List of studies included in analysis B. List of meta-analyses with overlapping associations excluded from analysis. Table S8. Characteristics of the conducted meta-analyses and results of the recalculation, the methodological assessment (AMSTAR 2), and the level of evidence by exposure. Table S9. Summary of results of the associations on various dietary factors and risk of RCC by study design. Table S10. Summary of results of the associations on various dietary factors and risk of RCC by region. Table S11. Sensitivity analysis and the level of evidence of the association of vegetables and vitamin C with RCC. [file 12916_2021_2229_MOESM1_ESM.pdf]

Additional File 1

Table S1: Embase Search Strategy

Table S2: Data extraction form for included systematic reviews and individual studies

Table S3: Citation matrices for meta-analyses with overlapping associations

Table S4: List of excluded studies and reasons for their exclusion

Table S5: AMSTAR 2 quality appraisal scores

Table S6: General characteristics of meta-analyses with overlapping associations

Table S7: A. List of studies included in analysis B. List of meta-analyses with overlapping associations excluded from analysis

Table S8: Characteristics of the conducted meta-analyses and results of the recalculation, the methodological assessment (AMSTAR 2), and the level of evidence by exposure

Table S9: Summary of results of the associations on various dietary factors and risk of RCC by study design

Table S10: Summary of results of the associations on various dietary factors and risk of RCC by region

Table S11: Sensitivity analysis and the level of evidence of the association of vegetables and vitamin C with RCC.

Table S1: Embase Search Strategy

Concept	Search Strategy	Results
#01	'diet'/exp	367889
#02	'dietetics'/exp	6931
#03	'nutrient'/exp	54589
#04	'food'/exp	1134670
#05	'beverage'/exp	246641
#06	'bread'/exp	8713
#07	'food grain'/exp	157593
#08	'maize'/exp	41250
#09	'soybean'/exp	31855
#10	'potato'/exp	14643
#11	'plant tuber'/exp	2054
#12	'fabaceae'/exp	108047
#13	'lentil'/exp	1663
#14	'vigna'/exp	3088
#15	'cicer'/exp	2214
#16	'oryza'/exp	43895
#17	'chenopodium quinoa'/exp	775
#18	'vegetable'/exp	217186
#19	'fruit'/exp	148745
#20	'milk'/exp	80588
#21	'dairy product'/exp	116725
#22	'yoghurt'/exp	5950
#23	'cheese'/exp	11285
#24	'egg'/exp	40806
#25	'meat'/exp	57722
#26	'pork'/exp	3473
#27	'chicken'/exp	140770
#28	'duck'/exp	10779
#29	'fish'/exp	250135
#30	'sea food'/exp	18248
#31	'shellfish'/exp	6212
#32	'butter'/exp	3427
#33	'margarine'/exp	1713
#34	'nut'/exp	21189
#35	'candy'/exp	1511
#36	'carbohydrate'/exp	1015385
#37	'alcohol'/exp	275102
#38	'caffeine'/exp	50270
#39	'coffee'/exp	15903
#40	'tea'/exp	20576
#41	'beer'/exp	7628

#42	'drinking'/exp	26173
#43	'wine'/exp	17994
#44	'sugar-sweetened beverage'/exp	2080
#45	'fatty acid'/exp	617035
#46	'dietary fiber'/exp	31377
#47	'starch'/exp	32064
#48	'fructose'/exp	26803
#49	'protein'/exp	494698
#50	'vitamin'/exp	708763
#51	'mineral'/exp	46090
#52	#1 OR #2 OR #3 OR #4 OR #5 OR #6 OR #7 OR #8 OR #9 OR #10 OR #11 OR #12 OR #13 OR #14 OR #15 OR #16 OR #17 OR #18 OR #19 OR #20 OR #21 OR #22 OR #23 OR #24 OR #25 OR #26 OR #27 OR #28 OR #29 OR #30 OR #31 OR #32 OR #33 OR #34 OR #35 OR #36 OR #37 OR #38 OR #39 OR #40 OR #41 OR #42 OR #43 OR #44 OR #45 OR #46 OR #47 OR #48 OR #49 OR #50 OR #51	4292156
#53	'diets':ab,ti OR 'nutrients':ab,ti OR 'macronutrients':ab,ti OR 'macronutrient':ab,ti OR 'foods':ab,ti OR 'beverages':ab,ti OR 'breads':ab,ti OR 'edible grain*':ab,ti OR 'grain, edible':ab,ti OR 'grains, edible':ab,ti OR 'cereal grain':ab,ti OR 'cereal grains':ab,ti OR 'grain, cereal':ab,ti OR 'grains, cereal':ab,ti OR 'cereals':ab,ti OR 'cereal':ab,ti OR 'zea':ab,ti OR 'corn':ab,ti OR 'indian corn':ab,ti OR 'corn, indian':ab,ti OR 'maize':ab,ti OR 'teosinte':ab,ti OR 'soybeans':ab,ti OR 'soy beans':ab,ti OR 'bean, soy':ab,ti OR 'beans, soy':ab,ti OR 'soy bean':ab,ti OR 'glycine max':ab,ti OR 'soy':ab,ti OR 'soya':ab,ti OR 'solanum tuberosum*':ab,ti OR 'tuberosum, solanum':ab,ti OR 'tuberosums, solanum':ab,ti OR 'potatoes':ab,ti OR 'potato':ab,ti OR 'plant tubers':ab,ti OR 'tuber, plant':ab,ti OR 'tubers, plant':ab,ti OR 'pulses':ab,ti OR 'legumes':ab,ti OR 'legume':ab,ti OR 'leguminosae':ab,ti OR 'pea family':ab,ti OR 'families, pea':ab,ti OR 'family, pea':ab,ti OR 'pea families':ab,ti OR 'amorpha':ab,ti OR 'andira':ab,ti OR 'baptisia':ab,ti OR 'callerya':ab,ti OR 'ceratonia':ab,ti OR 'clathrotropis':ab,ti OR 'tachigalia':ab,ti OR 'copaifera':ab,ti OR 'delonix':ab,ti OR 'euchresta':ab,ti OR 'guibourtia':ab,ti OR 'machaerium':ab,ti OR 'pithecellobium':ab,ti OR 'pithecolobium':ab,ti OR 'stryphnodendron':ab,ti OR 'afzelia':ab,ti OR 'colophospermum':ab,ti OR 'lens plant*':ab,ti OR 'plant, lens':ab,ti OR 'plants, lens':ab,ti OR 'lentils':ab,ti OR 'lentil':ab,ti OR 'lens culinaris':ab,ti OR 'lens culinari':ab,ti OR 'culinari, lens':ab,ti OR 'cowpeas':ab,ti OR 'cowpea':ab,ti OR 'vigna angularis':ab,ti OR 'adzuki bean':ab,ti OR 'adzuki beans':ab,ti OR 'bean, adzuki':ab,ti OR 'beans, adzuki':ab,ti OR 'vigna aconitifolia':ab,ti OR 'moth bean':ab,ti OR 'bean, moth':ab,ti OR 'beans, moth':ab,ti OR 'moth beans':ab,ti OR 'vigna radiata':ab,ti OR 'mung beans':ab,ti OR 'bean, mung':ab,ti OR	3582831

'beans, mung':ab,ti OR 'mung bean':ab,ti OR 'vigna unguiculata':ab,ti OR 'blackeyed pea':ab,ti OR 'blackeyed peas':ab,ti OR 'pea, blackeyed':ab,ti OR 'peas, blackeyed':ab,ti OR 'vigna mungo':ab,ti OR 'phaseolus mungo':ab,ti OR 'black gram':ab,ti OR 'gram, black':ab,ti OR 'vigna umbellata':ab,ti OR 'rice bean':ab,ti OR 'bean, rice':ab,ti OR 'beans, rice':ab,ti OR 'rice beans':ab,ti OR 'cicers':ab,ti OR 'cicer arietinum':ab,ti OR 'cicer arietinums':ab,ti OR 'arietinum, cicer':ab,ti OR 'chickpea':ab,ti OR 'chickpeas':ab,ti OR 'garbanzo':ab,ti OR 'garbanzos':ab,ti OR 'rice':ab,ti OR 'rices':ab,ti OR 'oryza sativa':ab,ti OR 'chenopodium quinoas':ab,ti OR 'quinoa, chenopodium':ab,ti OR 'quinoa':ab,ti OR 'quinoas':ab,ti OR 'vegetables':ab,ti OR 'fruits':ab,ti OR 'plant capsule':ab,ti OR 'capsule, plant':ab,ti OR 'capsules, plant':ab,ti OR 'plant capsules':ab,ti OR 'plant aril':ab,ti OR 'aril, plant':ab,ti OR 'arils, plant':ab,ti OR 'plant arils':ab,ti OR 'berries':ab,ti OR 'berry':ab,ti OR 'legume pod':ab,ti OR 'legume pods':ab,ti OR 'pod, legume':ab,ti OR 'pods, legume':ab,ti OR 'breast milk':ab,ti OR 'milk, breast':ab,ti OR 'human milk':ab,ti OR 'cow milk':ab,ti OR 'milk, cow':ab,ti OR 'dairy products':ab,ti OR 'product, dairy':ab,ti OR 'products, dairy':ab,ti OR 'yogurt':ab,ti OR 'cheeses':ab,ti OR 'eggs':ab,ti OR 'meats':ab,ti OR 'meat, pork':ab,ti OR 'meats, pork':ab,ti OR 'pork meats':ab,ti OR 'pig meat':ab,ti OR 'meat, pig':ab,ti OR 'meats, pig':ab,ti OR 'pig meats':ab,ti OR 'pork':ab,ti OR 'bacon':ab,ti OR 'cured ham':ab,ti OR 'ham, cured':ab,ti OR 'chickens':ab,ti OR 'gallus gallus domesticus':ab,ti OR 'gallus domesticus':ab,ti OR 'gallus gallus':ab,ti OR 'ducks':ab,ti OR 'fishes':ab,ti OR 'seafoods':ab,ti OR 'sea-food':ab,ti OR 'sea food':ab,ti OR 'sea-foods':ab,ti OR 'brachyura':ab,ti OR 'astacoidea':ab,ti OR 'nephropidae':ab,ti OR 'cephalopoda':ab,ti OR 'gastropoda':ab,ti OR 'bivalvia':ab,ti OR 'oil':ab,ti OR 'butters':ab,ti OR 'nuts':ab,ti OR 'dessert':ab,ti OR 'sweets':ab,ti OR 'sweetened':ab,ti OR 'candies':ab,ti OR 'confection':ab,ti OR 'confections':ab,ti OR 'caramel candy':ab,ti OR 'candies, caramel':ab,ti OR 'candy, caramel':ab,ti OR 'caramel candies':ab,ti OR 'sugar*':ab,ti OR 'alcohols':ab,ti OR '1,3,7-trimethylxanthine':ab,ti OR 'vivarin':ab,ti OR 'caffedrine':ab,ti OR 'coffeinum n':ab,ti OR 'percutaféine':ab,ti OR 'dexitac':ab,ti OR 'durvitan':ab,ti OR 'no doz':ab,ti OR 'percoffedrinol n':ab,ti OR 'quick-pep':ab,ti OR 'quick pep':ab,ti OR 'quickpep':ab,ti OR 'coffeinum purrum':ab,ti OR 'caffeine':ab,ti OR 'chicory':ab,ti OR 'coffea':ab,ti OR 'black tea':ab,ti OR 'black teas':ab,ti OR 'tea, black':ab,ti OR 'teas, black':ab,ti OR 'green tea':ab,ti OR 'green teas':ab,ti OR 'tea, green':ab,ti OR 'teas, green':ab,ti OR 'beers':ab,ti OR 'drinkings':ab,ti OR 'water consumption':ab,ti OR 'water intake':ab,ti OR 'wines':ab,ti OR 'beverage, sugar-sweetened':ab,ti OR 'beverages, sugar-sweetened':ab,ti OR 'sugar-sweetened beverage':ab,ti OR 'sugar-added

beverages':ab,ti OR 'sugar added beverages':ab,ti OR 'sugar sweetened beverage':ab,ti OR 'beverage, sugar sweetened':ab,ti OR 'beverages, sugar sweetened':ab,ti OR 'sugar sweetened beverages':ab,ti OR 'sweetened beverage, sugar':ab,ti OR 'sweetened beverages, sugar':ab,ti OR 'sugar-added beverage':ab,ti OR 'beverage, sugar-added':ab,ti OR 'beverages, sugar-added':ab,ti OR 'sugar added beverage':ab,ti OR 'sweetened drinks':ab,ti OR 'sweetened drink':ab,ti OR 'drink, sweetened':ab,ti OR 'drinks, sweetened':ab,ti OR 'sugar-sweetened soft drinks':ab,ti OR 'sugar sweetened soft drinks':ab,ti OR 'sugar-sweetened soft drink':ab,ti OR 'drink, sugar-sweetened soft':ab,ti OR 'drinks, sugar-sweetened soft':ab,ti OR 'soft drink, sugar-sweetened':ab,ti OR 'soft drinks, sugar-sweetened':ab,ti OR 'sugar sweetened soft drink':ab,ti OR 'sweetened beverages':ab,ti OR 'sweetened beverage':ab,ti OR 'beverage, sweetened':ab,ti OR 'beverages, sweetened':ab,ti OR 'sugar-sweetened sodas':ab,ti OR 'sugar sweetened sodas':ab,ti OR 'sugar-sweetened soda':ab,ti OR 'soda, sugar-sweetened':ab,ti OR 'sodas, sugar-sweetened':ab,ti OR 'sugar sweetened soda':ab,ti OR 'fatty acid':ab,ti OR 'fatty acids, esterified':ab,ti OR 'esterified fatty acids':ab,ti OR 'esterified fatty acid':ab,ti OR 'acid, esterified fatty':ab,ti OR 'fatty acid, esterified':ab,ti OR 'fatty acids, saturated':ab,ti OR 'saturated fatty acids':ab,ti OR 'saturated fatty acid':ab,ti OR 'acid, saturated fatty':ab,ti OR 'fatty acid, saturated':ab,ti OR 'aliphatic acids':ab,ti OR 'aliphatic acid':ab,ti OR 'acid, aliphatic':ab,ti OR 'carbohydrate':ab,ti OR 'dietary fibers':ab,ti OR 'fibers, dietary':ab,ti OR 'fiber, dietary':ab,ti OR 'wheat bran':ab,ti OR 'bran, wheat':ab,ti OR 'brans, wheat':ab,ti OR 'roughage':ab,ti OR 'roughages':ab,ti OR 'keoflo':ab,ti OR 'cornstarch':ab,ti OR 'amylomaize starch':ab,ti OR 'starch, amylomaize':ab,ti OR 'amylum':ab,ti OR 'sugar':ab,ti OR 'levulose':ab,ti OR 'fleboplast levulosa':ab,ti OR 'levulosa, fleboplast':ab,ti OR 'levulosa grifols':ab,ti OR 'levulosado vitulia':ab,ti OR 'levulosa braun':ab,ti OR 'levulosado braun':ab,ti OR 'levulosa ife':ab,ti OR 'levulosado bieffe medit':ab,ti OR 'apir levulosa':ab,ti OR 'levulosa, apir':ab,ti OR 'levulosa mein':ab,ti OR 'plast apyr levulosa mein':ab,ti OR 'levulosa ibys':ab,ti OR 'levulosa baxter':ab,ti OR 'levulosa':ab,ti OR 'proteins':ab,ti OR 'gene products, protein':ab,ti OR 'protein gene products':ab,ti OR 'gene proteins':ab,ti OR 'proteins, gene':ab,ti OR 'micronutrient':ab,ti OR 'vitamins':ab,ti OR 'minerals':ab,ti OR 'dietary':ab,ti OR 'nutrition':ab,ti OR 'food group':ab,ti OR 'food cluster':ab,ti OR 'grain':ab,ti OR 'wholegrain':ab,ti OR 'whole wheat':ab,ti OR 'granary':ab,ti OR 'tuber':ab,ti OR 'tubers':ab,ti OR 'beans':ab,ti OR 'pasta':ab,ti OR 'dairy':ab,ti OR 'pork meat':ab,ti OR 'lamb':ab,ti OR 'beef':ab,ti OR 'turkey':ab,ti OR 'carbohydrate*':ab,ti OR 'juice':ab,ti OR 'lemonade':ab,ti OR 'drinks':ab,ti OR 'liquor':ab,ti OR 'fat':ab,ti OR

	'fiber':ab,ti OR 'zea mays':ab,ti	
#54	#52 OR #53	6377703
#55	'renal cell carcinoma'/exp	16593
#56	'carcinoma, renal cell':ab,ti OR 'carcinomas, renal cell':ab,ti OR 'renal cell carcinomas':ab,ti OR 'nephroid carcinoma':ab,ti OR 'carcinoma, nephroid':ab,ti OR 'nephroid carcinomas':ab,ti OR 'adenocarcinoma of kidney':ab,ti OR 'adenocarcinoma of kidneys':ab,ti OR 'kidney, adenocarcinoma of':ab,ti OR 'renal cell carcinoma':ab,ti OR 'renal cell cancer':ab,ti OR 'cancer, renal cell':ab,ti OR 'renal cell cancers':ab,ti OR 'adenocarcinoma, renal':ab,ti OR 'renal adenocarcinoma':ab,ti OR 'renal adenocarcinomas':ab,ti OR 'renal carcinoma':ab,ti OR 'carcinoma, renal':ab,ti OR 'renal carcinomas':ab,ti OR 'adenocarcinoma, renal cell':ab,ti OR 'adenocarcinomas, renal cell':ab,ti OR 'renal cell adenocarcinoma':ab,ti OR 'renal cell adenocarcinomas':ab,ti OR 'chromophobe renal cell carcinoma':ab,ti OR 'sarcomatoid renal cell carcinoma':ab,ti OR 'papillary renal cell carcinoma':ab,ti OR 'renal cell carcinoma, papillary':ab,ti OR 'chromophil renal cell carcinoma':ab,ti OR 'clear cell renal cell carcinoma':ab,ti OR 'grawitz tumor':ab,ti OR 'tumor, grawitz':ab,ti OR 'clear cell renal carcinoma':ab,ti OR 'carcinoma, hypernephroid':ab,ti OR 'hypernephroid carcinoma':ab,ti OR 'hypernephroid carcinomas':ab,ti OR 'hypernephroma':ab,ti OR 'hypernephromas':ab,ti OR 'carcinoma, collecting duct (kidney)':ab,ti OR 'carcinomas, collecting duct (kidney)':ab,ti OR 'collecting duct carcinomas (kidney)':ab,ti OR 'collecting duct carcinoma of the kidney':ab,ti OR 'renal collecting duct carcinoma':ab,ti OR 'collecting duct carcinoma':ab,ti OR 'carcinoma, collecting duct':ab,ti OR 'carcinomas, collecting duct':ab,ti OR 'collecting duct carcinomas':ab,ti	69330
#57	#55 OR #56	73229
#58	systematic:ab,ti AND review*:ab,ti	293813
#59	medline:ab,ti OR 'meta analysis':ab,ti OR 'meta analysis':it	324884
#60	#58 OR #59	469327
#61	#54 AND #57 AND #60	142

Table S2: Data extraction from included systematic reviews and individual studies

A: Data extraction form for included systematic reviews

**Systematic reviews**	
Title	
Name of the first author	
Publication year	
Exposure (including dose of exposure)	
No. of included studies	
study design of the Individual studies	
No. of cohort study	
No. of case control study	
No. of pooled study	
Effects model	
No. of participants / No. of cases	
MA metric	
Estimates	
95%CI	
Type of comparison (high v low meta-analysis or dose-response meta-analysis)	
Range / Amount	
Quality score of Individual studies(mean) if reported	
Publication bias	
Heterogeneity	
Information on funding	
Conflict of interest	

MA: meta-analysis; CI: confidence intervals

B: Data extraction form for included individual studies

**Individual studies**	
Name of the first author	
Year of publication	
Exposure (including dose of exposure)	
Number of total cases	
Number of participants	
Hazard ratios (that adjust for the most confounders if reported) *	
95%CI*	

CI: confidence intervals; *: the data are used to estimate the statistical power of each individual study and to estimate excess significant bias.

The data extraction table is adapted from the data extraction table recommended by Joanna Briggs Institute.

Table S3: Citation matrices for meta-analyses with overlapping associations

A. Poultry

**Meta-analysis**	Faramawi 2007	Lee 2008
**Overlapping associations**	Renal cell carcinoma	Renal cell carcinoma
**Individual study**		
Chow 1994	X	
Lindblad 1997	X	
Maclure 1990	X	
Hu 2003	X	
Nicodemus 2004		X
Lin 2005		X
Lee 2006		X
Bernstein 2002		X
van Dijk 2005		X
Wolk 2006		X
Giles 2002		X
Rohan 2002		X
Mahabir 2005		X
Calle 2002		X
Flood 2002		X
**Total (No of publications per meta-analysis)**	**4**	**11**
**Grand Total (N)**	**15**	
**Rows (r)**	**15**	
**Columns (c)**	**2**	
**Corrected covered area (CCA)**	**0**	

Formula for calculating the corrected covered area, CCA (%) = N-r / rc-r

Where N = number of included publications (sum of checked boxes), r = number of rows (Individual publications), c = number of columns (number of meta-analyses).

B. Cruciferous Vegetables

**Meta-analysis**	Liu 2013	Zhao 2013
**Overlapping associations**	Renal cell carcinoma	Renal cell carcinoma
**Individual study**		
Wolk 1996	X	
Mellemgaard 1996	X	
Lindblad 1997	X	X
Yuan 1998	X	X
Hu 2003	X	X
van Dijk 2005	X	X
Lee 2006	X	X
Hsu 2007	X	X
Bertoia 2010	X	X
Brock 2011	X	
Daniel 2013		X
Brock 2012		X
Maclure 1990		X
Weikert 2006		X
Rashidkhani 2005		X
**Total (No of publications per meta-analysis)**	**10**	**12**
**Grand Total (N)**	**22**	
**Rows (r)**	**15**	
**Columns (c)**	**2**	
**Corrected covered area (CCA)**	**46.70%**	

Formula for calculating the corrected covered area, CCA (%) = N-r / rc-r

Where N = number of included publications (sum of checked boxes), r = number of rows (Individual publications), c = number of columns (number of meta-analyses).

C. Vitamin E

**Meta-analysis**	Shang 2015	Shen 2015
**Overlapping associations**	Renal cell carcinoma	Renal cell carcinoma
**Individual study**		
Bosetti 2006	X	
Wolk 1996	X	X
Lindblad 1997	X	X
Chow 1994	X	X
Mellemgaard 1996	X	X
Hu 2003	X	X
Hu 2009	X	X
Prineas 1997		X
Nicodemus 2004		X
Lee 2006		X
Bosetti 2007		X
van Dijk 2008		X
Bertoia 2010		X
Ho 2015		X
**Total (No of publications per meta-analysis)**	**7**	**13**
**Grand Total (N)**	**20**	
**Rows (r)**	**14**	
**Columns (c)**	**2**	
**Corrected covered area (CCA)**	**42.90%**	

Formula for calculating the corrected covered area, CCA (%) = N-r / rc-r

Where N = number of included publications (sum of checked boxes), r = number of rows (Individual publications), c = number of columns (number of meta-analyses).

D. Red meat

**Meta-analysis**	Alexander 2009	Faramawi 2007	Lee 2008	Zhang 2017
**Overlapping associations**	Renal cell carcinoma	Renal cell carcinoma	Renal cell carcinoma	Renal cell carcinoma
**Individual study**				
Bravi 2007	X			X
Cross 2007	X			
De Stefani 1998	X	X		X
Fraser 1990	X			X
Hsu 2007	X			X
Hu 2003	X	X		X
MaClure 1990	X	X		X
Talamini 1990	X			
Washio 2005	X			X
Wolk 1996	X			X
Lee 2008	X			X
Tavani 2000		X		X
Chow 1994		X		X
Handa 2002		X		X
Nicodemus 2004			X	
Lin 2005			X	
Lee 2006			X	
Bernstein 2002			X	
van Dijk 2005			X	
Wolk 2006			X	
Giles 2002			X	
Rohan 2002			X	

Mahabir 2005			X	
Bandera 2002			X	
Calle 2002			X	
Flood 2002			X	
Rohmann 2015				X
Daniel 2012				X
Melkonian 2016				X
Aune 2009				X
Daniel 2011				X
Brock 2009				X
Grieb 2009				X
**Total (No of publications per meta-analysis)**	**11**	**6**	**12**	**19**
**Grand Total (N)**	**48**			
**Rows (r)**	**33**			
**Columns (c)**	**4**			
**Corrected covered area (CCA)**	**15.20%**			

Formula for calculating the corrected covered area, CCA (%) = N-r / rc-r

Where N = number of included publications (sum of checked boxes), r = number of rows (Individual publications), c = number of columns (number of meta-analyses).

E. Processed meat

Meta-analysis	Alexander 2009	Faramawi 2007	Lee 2008	Zhang 2017
**Overlapping associations**	Renal cell carcinoma	Renal cell carcinoma	Renal cell carcinoma	Renal cell carcinoma
**Individual study**				
Bravi 2007	X			X
Cross 2007	X			
De Stefani 1998	X	X		X
Hsu 2007	X			X
Hu 2003	X	X		X
MaClure 1990	X	X		X
Washio 2005	X			X
Wolk 1996	X			X
Yuan 1998	X			X
Lee 2008	X			X
Chow 1994		X		X
Nicodemus 2004			X	
Lin 2005			X	
Lee 2006			X	
Bernstein 2002			X	
van Dijk 2005			X	
Wolk 2006			X	
Giles 2002			X	
Rohan 2002			X	
Mahabir 2005			X	
Bandera 2002			X	
Calle 2002			X	

Flood 2002			X	
Rohrmann 2015				X
Daniel 2012				X
De Stefani 2012				X
Aune 2009				X
Talamini 1990				X
Hu 2011				X
Daniel 2011				X
Brock 2009				X
Grieb 2009				X
**Total (No of publications per meta-analysis)**	**10**	**4**	**12**	**19**
**Grand Total (N)**	**47**			
**Rows (r)**	**32**			
**Columns (c)**	**4**			
**Corrected covered area (CCA)**	**15.60%**			

Formula for calculating the corrected covered area, CCA (%) = N-r / rc-r

Where N = number of included publications (sum of checked boxes), r = number of rows (Individual publications), c = number of columns (number of meta-analyses)

F. Alcohol

Meta-analysis	Bellocco 2012	Cheng 2011	Song 2012	Xu 2015	WCRF 2018
**Overlapping associations**	Renal cell carcinoma	Renal cell carcinoma	Renal cell carcinoma	Renal cell carcinoma	Renal cell carcinoma
**Individual study**					
Goodman 1986	X	X	X		
Brownson 1988	X	X	X		
Kreiger 1993	X	X	X		
Benhamou 1993	X	X	X		
Mellemgaard 1994	X				
Wolk 1996	X	X	X		
Lindblad 1997	X				
Yuan 1998	X	X	X		
Parker 2002	X	X	X		
Mattioli 2002	X	X	X		
Greving 2007	X	X	X		
Hsu 2007	X	X	X		
Hu 2008	X		X		
Pelucchi 2008	X	X	X		
Benedetti 2009	X	X	X		
Setiawan 2007	X		X	X	X
Lee 2007	X		X	X	
Ozasa 2007	X			X	
Allen 2009	X			X	
Kim 2010	X			X	
Maclure 1990		X	X		
Brock 2009		X			

Fu 2008 2009		X			
Wynder 1974			X		
Mclaughlin 1984			X		
Asal 1988			X		
Hiatt 1994			X		
Muscat 1995			X		
Boeing 1997			X		
Allen 2011			X		X
Lew 2011			X	X	X
Karamini 2014				X	
Macleod 2013				X	
Wilson 2009					X
Schouten 2008					X
Rashidkhani 2005					X
Nicodemus 2004					X
**Total (No of publications per meta-analysis)**	**20**	**15**	**24**	**8**	**7**
**Grand Total (N)**	**74**				
**Rows (r)**	**37**				
**Columns (c)**	**5**				
**Corrected covered area (CCA)**	**25.00%**				

Formula for calculating the corrected covered area, CCA (%) = N-r / rc-r

Where N = number of included publications (sum of checked boxes), r = number of rows (Individual publications), c = number of columns (number of meta-analyses)

G. Dietary fiber

**Meta-analysis**	Huang 2014	Xu 2019
**Overlapping associations**	Renal cell carcinoma	Renal cell carcinoma
**Individual study**		
Daniel 2013	X	X
Wolk 1996	X	X
Lindblad 1997	X	
Allen 2009	X	X
Hu 2008	X	X
Galeone 2007	X	X
Brock 2012		X
Zhu 2017		X
**Total (No of publications per meta-analysis)**	**6**	**7**
**Grand Total (N)**	**13**	
**Rows (r)**	**8**	
**Columns (c)**	**2**	
**Corrected covered area (CCA)**	**62.50%**	

Formula for calculating the corrected covered area, CCA (%) = N-r / rc-r

Where N = number of included publications (sum of checked boxes), r = number of rows (Individual publications), c = number of columns (number of meta-analyses)

H. Coffee

**Meta-analysis**	Huang 2014	Wijarnpreecha 2017
**Overlapping associations**	Renal cell carcinoma	Renal cell carcinoma
**Individual study**		
Jacobsen 1986	X	
Stensvold 1994	X	
Washio 2005	X	X
Lee 2006	X	X
Armstrong 1976		X
Asal 1988		X
Benhamou 1993		X
Bravi 2007		X
Goodman 1986		X
Hu 2009		X
Kreiger 1993		X
Maclure 1990		X
McLaughlin 1984		X
Mellemgaard 1994		X
Montella 2009		X
Mucci 2004		X
Talamini 1990		X
Wolk 1996		X
Yu 1986		X
Yuan 1998		X
Allen 2011		X
Hashibe 2015		X
Lee 2007		X
Nilsson 2010		X
**Total (No of publications per meta-analysis)**	**4**	**22**
**Grand Total (N)**	**26**	
**Rows (r)**	**24**	
**Columns (c)**	**2**	
**Corrected covered area (CCA)**	**8.30%**	

Formula for calculating the corrected covered area, CCA (%) = N-r / rc-r

Where N = number of included publications (sum of checked boxes), r = number of rows (Individual publications), c = number of columns (number of meta-analyses)

Table S4: List of excluded studies and reasons for their exclusion

	1st Author	Year	Title	Reason for Exclusion
1	Adams VR	2007	Sunitinib malate for the treatment of metastatic renal cell carcinoma and gastrointestinal stromal tumors	not related to the topic
2	Arab L	2010	Epidemiologic Evidence on Coffee and Cancer	no meta-analysis
3	Bara T	2017	A systematic review of the possible carcinogenic role of the aristolochic acid	not related to the topic
4	Biggar PH	2011	Vitamin D, chronic kidney disease and survival: a pluripotent hormone or just another bone drug?	not related to the topic
5	Caprio GG	2020	Light Alcohol Drinking and the Risk of Cancer Development: A Controversial Relationship	no meta-analysis
6	Chaudhari PB	2017	Nivolumab - Pearls of Evidence	not related to the topic
7	Fang J	2020	Lower circulating adiponectin is associated with higher risk of renal cell carcinoma: A meta-analysis	not related to the topic
8	Fox CH	2002	Phytic acid (IP6), novel broad spectrum anti-neoplastic agent: a systematic review	not related to the topic
9	Gandini S	2014	Vitamin D receptor polymorphisms and cancer	not related to the topic
10	Gnagnarella P	2020	Vitamin D Receptor Polymorphisms and Cancer	not related to the topic
11	Gouveia MC	2016	Association between irisin and major chronic diseases: a review	exposure of plasma levels or biomarkers
12	Graff RE	2018	Dietary Acrylamide Intake and Risk of Renal Cell Carcinoma in Two Large Prospective Cohorts	individual studies
13	Händel MN	2019	Processed meat intake and chronic disease morbidity and mortality: An overview of systematic reviews and meta-analyses	no meta-analysis
14	Il'yasova D	2005	Cadmium and renal cancer	not related to the topic
15	Jeyaraman MM	2019	Dairy product consumption and development of cancer: An overview of reviews	no meta-analysis
16	Kabaria R	2016	Renal cell carcinoma: links and risks	no meta-analysis
17	Kamat AM	1999	Chemoprevention of urological cancer	no meta-analysis
18	Key T	2004	Diet, nutrition and the prevention of cancer	no meta-analysis

19	Khan MI	2014	Vitamin D receptor gene polymorphisms in breast and renal cancer: Current state and future approaches	not related to the topic
20	Krajewski W	2016	Vitamin D and urological cancers	no meta-analysis
21	Kume H	2001	Risk factors for adult renal cell carcinoma: a systematic review and implications for prevention	comment
22	Latino-Martel P	2016	Alcoholic beverages, obesity, physical activity and other nutritional factors, and cancer risk: A review of the evidence	no meta-analysis
23	Lenko V	2013	The relationship between renal cell carcinoma and nuclear retinoid/rexinoid receptors	not related to the topic
24	Ljungberg B	2011	The Epidemiology of Renal Cell Carcinoma	no meta-analysis
25	Luong KVQ	2010	The beneficial role of vitamin D and its analogs in cancer treatment and prevention	no meta-analysis
26	Miller PE	2012	Phytochemicals and Cancer Risk: A Review of the Epidemiological Evidence	no meta-analysis
27	Niu XH	2021	Prognostic significance of pretreatment controlling nutritional status score in urological cancers: a systematic review and meta-analysis	not related to the topic
28	Ouzzane A	2014	Epidemiology and risk factors of upper urinary tract tumors: Literature review for the yearly scientific report of the French National Association of Urology	not published in English
29	Riboldi BP	2014	Risks of dietary acrylamide exposure: A systematic review	no meta-analysis
30	Rumrich IK	2016	Maternal Smoking and the Risk of Cancer in Early Life - A Meta-Analysis	not related to the topic
31	Stelmach RD	2015	Household Water Quantity and Health: A Systematic Review	no meta-analysis
32	Strohle A	2006	Vegetarian nutrition: Preventive potential and possible risks Part 1: Plant foods	not related to the topic
33	Teissedre PL	2020	Effects of alcohol consumption in general, and wine in particular, on the risk of cancer development: a review	no meta-analysis
34	Virk-Baker MK	2014	Dietary Acrylamide and Human Cancer: A Systematic Review of Literature	no meta-analysis
35	Weikert S	2010	Contemporary epidemiology of renal cell carcinoma: perspectives of primary prevention	no meta-analysis

36	Wu F	2013	Risk Assessment of Upper Tract Urothelial Carcinoma Related to Aristolochic Acid	not related to the topic
37	Al-Bayati O	2019	Systematic review of modifiable risk factors for kidney cancer	no meta-analysis
38	Darling AL	2016	WCRF-AICR continuous update project: Systematic literature review of prospective studies on circulating 25-hydroxyvitamin D and kidney cancer risk	exposure of plasma levels or biomarkers
39	Dhote R	2004	Risk factors for adult renal cell carcinoma	no meta-analysis
40	Dhôte R	2000	Risk factors for adult renal cell carcinoma: a systematic review and implications for prevention	no meta-analysis
41	Javed F	2010	Systemic conditions associated with areca nut usage: A literature review	not related to the topic
42	Jiang F	2020	No association between dietary acrylamide and renal cell carcinoma: an updated meta-analysis	not related to the topic
43	Johnson C	2015	The Science of Salt: A Systematic Review of Clinical Salt Studies 2013 to 2014	no meta-analysis
44	Kim JY	2009	Garlic intake and cancer risk: an analysis using the Food and Drug Administration's evidence-based review system for the scientific evaluation of health claims	no meta-analysis
45	Lin GZ	2015	Examining the association of circulating 25-hydroxyvitamin D with kidney cancer risk: a meta-analysis	exposure of plasma levels or biomarkers
46	Lipworth L	2006	The epidemiology of renal cell carcinoma	no meta-analysis
47	Mao BJ	2015	One-Carbon Metabolic Factors and Risk of Renal Cell Cancer: A Meta-Analysis	exposure of plasma levels or biomarkers
48	Pelucchi C	2015	Dietary acrylamide and cancer risk: An updated meta-analysis	not related to the topic
49	Pelucchi C	2011	Exposure to acrylamide and human cancer-a review and meta-analysis of epidemiologic studies	not related to the topic
50	Rosato V	2018	Processed Meat and Risk of Renal Cell and Bladder Cancers	no meta-analysis
51	Yip CSC	2019	The Associations of Fruit and Vegetable Intakes with Burden of Diseases: A Systematic Review of Meta-Analyses	no meta-analysis
52	Yu XF	2011	Coffee consumption and risk of cancers: a meta-analysis of cohort studies	not related to the topic
53	Navai N	2012	Environmental and modifiable risk factors in renal cell carcinoma	no meta-analysis

54	Washio M	2011	Risk Factors for Kidney Cancer (Renal Cell Carcinoma) in a Japanese Population: A Mini-Review	no meta-analysis
55	Boeing H	1997	Diet, obesity and risk for renal cell carcinoma: results from a case control-study in Germany	individual studies
56	Gallicchio L	2010	Circulating 25-hydroxyvitamin D and risk of kidney cancer: Cohort Consortium Vitamin D Pooling Project of Rarer Cancers	exposure of plasma levels or biomarkers
57	WCRF	-	Kidney Cancer Protocol Version 1. Continuous update of the epidemiological evidence on food, nutrition, physical activity and the risk of kidney cancer	protocol
58	WCRF	2018	World Cancer Research Fund/American Institute for Cancer Research. Continuous Update Project Expert Report 2018. Diet, nutrition, physical activity and kidney cancer	reviews with overlapping associations
59	Alexander DD	2009	Quantitative assessment of red meat or processed meat consumption and kidney cancer	reviews with overlapping associations
60	Bellocco R	2012	Alcohol drinking and risk of renal cell carcinoma: results of a meta-analysis	reviews with overlapping associations
61	Huang TB	2014	Coffee consumption and urologic cancer risk: a meta-analysis of cohort studies	reviews with overlapping associations
62	Liu B	2013	Cruciferous Vegetables Consumption and Risk of Renal Cell Carcinoma: A Meta-Analysis	reviews with overlapping associations
63	Shang YG	2015	Vitamin E Intake and Risk of Renal Cell Carcinoma: A Meta-Analysis of 7 Case-Control Studies	reviews with overlapping associations
64	Song DY	2012	Alcohol intake and renal cell cancer risk: a meta-analysis	reviews with overlapping associations

Table S5: AMSTAR 2 quality appraisal scores

Item No	1	2※	3	4※	5	6	7※	8	9※	10	11※	12	13※	14	15※	16	Overall Rating
Alexander 2009	Yes	No	No	No	No	No	No	Yes	No	No	Yes	Yes	Yes	Yes	Yes	Yes	critically low
Alizadeh 2017	Yes	No	No	Partial Yes	No	Yes	No	Yes	Yes	No	Yes	Yes	Yes	Yes	Yes	Yes	moderate
Bai 2013	Yes	No	No	Partial Yes	Yes	Yes	No	Yes	Yes	No	Yes	Yes	Yes	Yes	Yes	Yes	low
Bellocco 2012	Yes	No	No	Partial Yes	Yes	No	No	Yes	Yes	No	Yes	Yes	Yes	Yes	No	Yes	moderate
Boyle 2014	Yes	No	Yes	Partial Yes	No	Yes	No	No	No	No	No	No	No	No	No	Yes	critically low
Cheng 2011	Yes	No	No	No	No	No	No	Yes	No	No	Yes	Yes	Yes	Yes	Yes	No	critically low
Clasen 2020	Yes	No	No	Partial Yes	Yes	Yes	No	Yes	Yes	No	No	Yes	Yes	No	Yes	Yes	low
Faramawi 2007	Yes	No	No	Partial Yes	Yes	Yes	No	Yes	No	No	Yes	Yes	Yes	Yes	No	No	low
Hu 2013	Yes	No	No	Partial Yes	Yes	Yes	No	Yes	No	No	Yes	Yes	Yes	No	Yes	No	low
Huang 2014	Yes	No	No	Partial Yes	Yes	Yes	No	Yes	Yes	No	Yes	Yes	Yes	Yes	Yes	Yes	moderate
Huang 2014	Yes	No	No	Partial	Yes	Yes	No	Yes	No	No	Yes	No	Yes	Yes	No	Yes	low

				Yes													
Jayedi 2018	Yes	No	No	Partial Yes	Yes	Yes	No	Yes	Yes	No	Yes	Yes	Yes	Yes	No	Yes	moderate
Jia 2015	Yes	No	No	Partial Yes	Yes	Yes	No	Yes	No	No	Yes	Yes	Yes	Yes	Yes	Yes	low
Lee 2008	Yes	No	No	No	No	No	No	Yes	No	No	No	No	No	Yes	No	Yes	critically low
Liu 2013	Yes	No	No	Partial Yes	Yes	No	No	Yes	No	No	Yes	No	Yes	No	Yes	No	low
Llaha 2021	Yes	No	No	Partial Yes	Yes	Yes	No	Yes	Yes	No	Yes	Yes	Yes	Yes	No	Yes	moderate
Shang 2015	Yes	No	No	No	Yes	No	No	Yes	Yes	No	Yes	Yes	Yes	Yes	No	Yes	low
Shen 2015	Yes	No	No	Partial Yes	Yes	Yes	No	Yes	No	No	No	Yes	Yes	Yes	Yes	Yes	critically low
Song 2012	Yes	No	No	Partial Yes	Yes	Yes	No	Yes	Yes	No	Yes	No	No	Yes	No	No	low
Wijarnpreecha 2017	Yes	No	No	Partial Yes	Yes	Yes	No	Yes	Yes	No	Yes	Yes	Yes	Yes	No	Yes	moderate
Wu 2020	Yes	No	No	Partial Yes	Yes	Yes	No	Yes	Yes	No	Yes	Yes	Yes	Yes	Yes	Yes	moderate
Xie 2016	Yes	No	No	No	Yes	Yes	No	Yes	No	No	No	Yes	Yes	Yes	No	Yes	critically low
Xu 2019	Yes	No	No	Partial Yes	Yes	Yes	No	Yes	Yes	No	No	No	No	Yes	No	Yes	critically low

Xu 2015	Yes	No	No	Partial Yes	Yes	Yes	No	Yes	Yes	No	Yes	Yes	Yes	Yes	No	Yes	moderate
Zhang 2017	Yes	No	No	Partial Yes	Yes	Yes	No	Yes	Yes	No	No	Yes	Yes	Yes	Yes	Yes	critically low
Zhang 2017	Yes	No	No	Partial Yes	Yes	Yes	No	Yes	Yes	No	Yes	Yes	Yes	Yes	Yes	Yes	moderate
Zhao 2013	Yes	No	No	Partial Yes	Yes	Yes	No	Yes	Yes	No	Yes	Yes	Yes	Yes	Yes	Yes	moderate
WCRF 2013	Yes	Yes	Yes	Partial Yes	Yes	Yes	Yes	Yes	No	No	Yes	Yes	Yes	Yes	Yes	No	low
WCRF 2018	Yes	Yes	Yes	Partial Yes	Yes	Yes	Yes	Yes	No	No	Yes	Yes	Yes	Yes	Yes	No	low

※: critical domains. Item 1: inclusion of PICO elements? Item 2: review methods established before conduct of review? Item 3: explanation for selection of study designs to be included in review? Item 4: use of a comprehensive search strategy? Item 5: selection of studies in duplicate? Item 6: data extraction in duplicate? Item 7: provision of list of excluded studies with justification for exclusion? Item 8: description of included studies in adequate detail? Item 9: satisfactory technique for risk of bias? Item 10: sources of funding for included studies reported? Item 11: proper methods for meta-analysis? Item 12: potential risk of bias in included studies discussed? Item 13: risk of bias accounted for in interpreting results? Item 14: heterogeneity discussed? Item 15: if meta-analysis conducted was publication bias discussed? Item 16: disclosure of funding or conflict of interest?

Table S6: General characteristics of meta-analyses with overlapping associations

Exposure	1st author	Year	No of primary studies	AMSTAR 2 rating	Corrected covered area (CCA)	Decision to retain ✓ = Yes ✘ = No
Red meat	Alexander	2009	11	critically low	15.20%	✘
	Faramawi	2007	6	low		✘
	Lee	2008	13	critically low		✘
	Zhang	2017	19	moderate		✓
Processed meat	Alexander	2009	10	critically low	15.60%	✘
	Faramawi	2007	4	low		✘
	Lee	2008	13	critically low		✘
	Zhang	2017	19	moderate		✓
Alcohol	Bellocco	2012	20	moderate	25.00%	✘
	Cheng	2011	15	critically low		✘
	Song	2012	24	low		✘
	Xu	2015	8	moderate		✓
	WCRF	2018	7	low		✘
Poultry*	Faramawi	2007	4	low	0	✓
	Lee	2008	12	critically low		✓
Dietary fiber	Huang	2014	6	moderate	62.50%	✘
	Xu	2019	7	critically low		✓
Cruciferous Vegetables	Liu	2013	10	low	46.70%	✘
	Zhao	2013	12	moderate		✓
Vitamin E	Shang	2015	7	low	42.90%	✘
	Shen	2015	13	critically low		✓
Coffee	Huang	2014	4	low	8.30%	✘
	Wijarnpreecha	2017	22	moderate		✓

*: Both articles were retained because the overlapping was 0.

Table S7:

A. List of studies included in analysis

	Name of the first author	Publication year	Title
**1**	Shahab Alizadeh	2017	A posteriori dietary patterns and risk of pancreatic and renal cancers: a systematic review and meta-analysis
**2**	Hong-wei Bai	2013	The Association between Fish Consumption and Risk of Renal Cancer: A Meta-Analysis of Observational Studies
**3**	Peter Boyle	2014	Sweetened carbonated beverage consumption and cancer risk: meta-analysis and review
**4**	Guang Cheng	2011	Alcohol intake and risk of renal cell carcinoma: a meta-analysis of published case-control studies
**5**	Joanna L. Clasen	2020	Components of one-carbon metabolism and renal cell carcinoma: a systematic review and meta-analysis
**6**	Mohammed F. Faramawi	2007	Consumption of different types of meat and the risk of renal cancer: meta-analysis of case–control studies
**7**	Zheng-Hui Hu	2013	No Association Between Tea Consumption and Risk of Renal Cell Carcinoma: A Meta-analysis of Epidemiological Studies
**8**	Tian-bao Huang	2014	Dietary fiber intake and risk of renal cell carcinoma: evidence from a meta-analysis
**9**	Ahmad Jayedi	2018	Dietary Inflammatory Index and Site-Specific Cancer Risk: A Systematic Review and Dose-Response Meta-Analysis
**10**	Li Jia	2015	Vitamin C intake and risk of renal cell carcinoma: a meta-analysis
**11**	Jung Eun Lee	2008	Fat, Protein, and Meat Consumption and Renal Cell Cancer Risk: A Pooled Analysis of 13 Prospective Studies
**12**	Fjorida Llaha	2021	Consumption of Sweet Beverages and Cancer Risk. A Systematic Review and Meta-Analysis of Observational Studies
**13**	Chongxing Shen	2015	Association of Vitamin E Intake with Reduced Risk of Kidney Cancer: A Meta-Analysis of Observational Studies
**14**	Karn Wijarnpree cha	2017	Association between coffee consumption and risk of renal cell carcinoma: a meta-analysis
**15**	Jing Wu	2020	Dietary and circulating vitamin D and risk of renal cell carcinoma: a meta-analysis of observational studies
**16**	Li Xie	2016	Association between dietary nitrate and nitrite intake and site- specific cance r risk: evidence from observational studies
**17**	Xin Xu	2019	Dietary fiber, glycemic index, glycemic load, and renal cell carcinoma risk
**18**	Xin Xu	2015	Does beer, wine or liquor consumption correlate with the risk of renal cell ca rcinoma? A dose-response meta analysis of prospective cohort studies
**19**	Shaojin Zhang	2017	Consumption of fruits and vegetables and risk of renal cell carcinoma: a meta-analysis of observational studies
**20**	Shaojing Zhang	2017	Intake of red and processed meat and risk of renal cell carcinoma: a meta-analysis of observational studies

**21**	Jun Zhao	2013	Cruciferous Vegetables Intake Is Associated with Lower Risk of Renal Cell Carcinoma: Evidence from a Meta-Analysis of Observational Studies
**22**	WCRF	2013	The Associations between Diet, Nutrition and Physical Activity and the Risk of Kidney Cancer

B. List of meta-analyses with overlapping associations excluded from analysis

**1**	Dominik D. Alexander	2009	Quantitative assessment of red meat or processed meat consumption and kidney cancer
**2**	R. Bellocco	2012	Alcohol drinking and risk of renal cell carcinoma: results of a meta-analysis
**3**	Tian-bao Huang	2014	Coffee consumption and urologic cancer risk: a meta-analysis of cohort studies
**4**	Ben Liu	2013	Cruciferous Vegetables Consumption and Risk of Renal Cell Carcinoma: A Meta-Analysis
**5**	Yonggang Shang	2015	Vitamin E Intake and Risk of Renal Cell Carcinoma: A Meta-Analysis of 7 Case–Control Studies
**6**	DY Song	2012	Alcohol intake and renal cell cancer risk: a meta-analysis
**7**	WCRF	2018	Continuous Update Project Expert Report 2018. Diet, nutrition, physical activity and kidney cancer

Table S8: Characteristics of the conducted meta-analyses and results of the recalculation, the methodological assessment (AMSTAR 2) and the level of evidence by exposure

Exposure	Reference	No of primary studies	Comparison	Rang/amount	No. of participants / No. of cases	Adjusted SHR (95%-CI)	p-value	I^2	τ^2	95%-PI	Egger's p-value	AMSTAR2	Level of evidence
**Dietary patterns or diet quality indices**													
Healthy dietary pattern	Alizadeh 2017	4	High v low intake	higher adherence v lower adherence	51709/1327	0.66 (0.51 to 0.85)	0.00145	19.40%	0.0269	0.27 to 1.63	0.1278748	moderate	IV
Unhealthy/western pattern	Alizadeh 2017	4	High v low intake	higher adherence v lower adherence	51709/1327	1.54 (1.19 to 2.01)	0.001232	24.70%	0.027	0.62 to 3.84	0.4200446	moderate	IV
Drinking pattern	Alizadeh 2017	2	High v low intake	higher adherence v lower adherence	47705/554	0.69 (0.47 to 1.01)	0.057101	0.00%	0.0002	NA	NA	moderate	NS
Dietary Inflammatory Index	Jayedi 2018	2	dose-response	1-unit increment	36118/1030	1.08 (1.03 to 1.13)	0.00276	0.00%	< 0.0001	NA	NA	moderate	IV
Glycemic index	Xu 2019	5	High v low intake	higher adherence v lower adherence	502119/4281	1.18 (1.02 to 1.37)	0.026826	26.00%	0.0099	0.79 to 1.75	0.3402257	critically low	IV
Glycemic load	Xu 2019	5	High v low intake	higher adherence v lower adherence	502119/4281	1.16 (0.73 to 1.84)	0.522525	82.40%	0.2346	0.21 to 6.45	0.8561544	critically low	NS
**Foods**													
Fish	Bai 2013	15	High v low intake	higher adherence v lower adherence	608753/9324	0.97 (0.84 to 1.10)	0.612245	24.10%	0.0335	0.63 to 1.47	0.2963335	low	NS
All meat	Faramawi 2007	11	High v low intake	higher adherence v lower adherence	15237/4835	1.27 (1.08 to 1.49)	0.003529	0.00%	0.0288	0.83 to 1.94	0.5296527	low	IV
Seafood*	Lee 2008	12	High v low intake	higher adherence v lower adherence	NA/1375	1.05 (0.82 to 1.35)	NA	NA	NA	NA	NA	critically low	-
Vegetables	Zhang 2017	16	High v low intake	higher adherence v lower adherence	1365634/9353	0.74 (0.63 to 0.86)	0.000073	51.30%	0.0507	0.44 to 1.23	0.04925753	critically low	III

Fruits	Zhang 2017	17	High v low intake	higher adherence v lower adherence	1398778/9150	0.84 (0.69 to 1.02)	0.075352	50.90%	0.1158	0.39 to 1.78	0.1237748	critically low	NS
Red meat†	Zhang 2017	19	High v low intake	higher adherence v lower adherence	1826171/12631	1.40 (1.15 to 1.70)	0.000807	71.40%	0.1353	0.63 to 3.13	0.003128808	moderate	III
Processed meat	Zhang 2017	19	High v low intake	higher adherence v lower adherence	1793675/14240	1.13 (1.01 to 1.26)	0.030876	46.80%	0.0282	0.78 to 1.64	0.8249137	moderate	IV
Poultry[#]	Faramawi 2007; Lee 2008	16	High v low intake	higher adherence v lower adherence	NA/4165	1.23 (1.02 to 1.47)	0.030824	0.00%	0.005	0.88 to 1.70	0.2519625	low; critically low	IV
Cruciferous vegetables	Zhao 2013	12	High v low intake	higher adherence v lower adherence	1228518/5773	0.81 (0.71 to 0.92)	0.001107	55.70%	0.0271	0.54 to 1.20	0.3483077	moderate	IV
Fruit and non-starchy vegetables	WCRF 2013	7	Dose-response	per 100 g/d	756544/1215	0.99 (0.93 to 1.05)	0.748236	0.00%	0.0021	0.85 to 1.15	0.07948871	low	NS
Non-starchy vegetables	WCRF 2013	8	Dose-response	per 100 g/d	1248385/3031	0.99 (0.91 to 1.09)	0.942029	0.00%	0.0051	0.80 to 1.24	0.7777938	low	NS
Tomatoes	WCRF 2013	3	Dose-response	per 50 g/d	NA/427	1.11 (0.92 to 1.35)	0.28285	0.00%	0.0024	0.27 to 4.51	0.8397414	low	NS
Citrus fruit	WCRF 2013	7	Dose-response	per 50 g/d	NA/2735	0.98 (0.90 to 1.05)	0.53534	0.00%	0.0034	0.80 to 1.19	0.8748599	low	NS
**Beverages**													
Sweetened carbonated beverage*	Boyle 2014	5	High v low intake	higher adherence v lower adherence	NA/NA	1.35 (0.86 to 2.11)	0.187219	60.10%	0.1927	0.28 to 6.51	0.5148688	critically low	NS
Sweet beverages (include ASB and SSB)	Llaha 2021	3	High v low intake	higher adherence v lower adherence	143194/1589	1.33 (0.74 to 2.40)	0.336717	85.60%	0.2482	0.0008 to 2156.90	0.5013056	moderate	NS
Tea	Hu 2013	12	High v low intake	higher adherence v lower adherence	1571852/6636	1.05 (0.83 to 1.33)	0.680841	71.50%	0.1348	0.44 to 2.49	0.7843664	low	NS
Coffee	Wijarnpreecha 2017	22	High v low intake	higher adherence v	1991863/NA	0.99 (0.84 to 1.18)	0.949021	32.60%	0.1006	0.50 to 1.98	0.9259041	moderate	NS

				lower adherence									
**Alcoholic beverages**													
Beer	Cheng 2011	8	High v low intake	higher adherence v lower adherence	NA/5538	0.84 (0.70 to 1.00)	0.052184	14.80%	0.0288	0.52 to 1.34	0.2440552	critically low	NS
Wine	Cheng 2011	8	High v low intake	higher adherence v lower adherence	NA/5538	0.70 (0.52 to 0.94)	0.019297	56.00%	0.1077	0.30 to 1.70	0.8069916	critically low	IV
Spirits	Cheng 2011	9	High v low intake	higher adherence v lower adherence	NA/6393	0.80 (0.64 to 0.99)	0.048382	74.30%	0.0768	0.39 to 1.62	0.503464	critically low	IV
Alcohol (light)	Xu 2015	6	dose-response	<12.5 g/d	3720850/5214	0.91 (0.81 to 1.02)	0.102662	39.60%	0.0116	0.64 to 1.28	0.1086253	moderate	NS
Alcohol (moderate)	Xu 2015	8	dose-response	12.5 - 37.5 g/d	4139301/5364	0.77 (0.58 to 1.02)	0.069063	29.20%	0.1288	0.30 to 1.98	0.7937301	moderate	NS
Alcohol (heavy)	Xu 2015	3	dose-response	>37.5 g/d	2065995/363	0.93 (0.27 to 3.22)	0.9134	81.20%	1.0389	0.0000 to 3904180.81	0.5801953	moderate	NS
Alcohol (any)	Xu 2015	8	High v low intake	higher adherence v lower adherence	4867646/5503	0.88 (0.63 to 1.22)	0.439292	62.90%	0.1971	0.27 to 2.81	0.8064805	moderate	NS
**Macronutrients**													
Fruit fiber	Huang 2014	3	High v low intake	higher adherence v lower adherence	496292/2906	0.91 (0.74 to 1.11)	0.346662	0.00%	0.0119	0.14 to 6.06	0.6841645	moderate	NS
Vegetable fiber	Huang 2014	3	High v low intake	higher adherence v lower adherence	496292/2906	0.68 (0.44 to 1.06)	0.089923	76.90%	0.1241	0.003 to 139.60	0.09604394	moderate	NS
Cereal fiber	Huang 2014	3	High v low intake	higher adherence v lower adherence	496292/2906	1.05 (0.88 to 1.26)	0.569512	0.00%	0.0067	0.22 to 4.94	0.6435649	moderate	NS
Legume fiber*	Lee 2008	1	High v low intake	higher adherence v lower adherence	491841/1816	0.80 (0.69 to 0.93)	NA	NA	NA	NA	NA	moderate	-
Total fat	WCRF 2013	14	Dose-response	5% energy	1210245/1985	1.02 (0.98 to 1.07)	0.360753	0.00%	< 0.0001	NA	NA	low	NS
Saturated fat	WCRF 2013	14	Dose-response	5% energy	1210245/1985	1.05 (0.88 to 1.24)	0.60759	41.30%	0.0072	NA	NA	low	NS
Monounsaturated fat	WCRF 2013	14	Dose-response	5% energy	1210245/1985	0.98 (0.83 to 1.15)	0.762472	0.00%	0.0022	NA	NA	low	NS

Polyunsaturated fat	WCRF 2013	14	Dose-response	5% energy	1210245/1985	0.90 (0.75 to 1.07)	0.219824	18.90%	0.0056	NA	NA	low	NS
Animal fat*	Lee 2008	8	High v low intake	higher adherence v lower adherence	417093/900	1.01 (0.96 to 1.07)	NA	NA	NA	NA	NA	critically low	-
Plant fat*	Lee 2008	8	High v low intake	higher adherence v lower adherence	417093/900	0.99 (0.93 to 1.05)	NA	NA	NA	NA	NA	critically low	-
Cholesterol*	Lee 2008	13	High v low intake	higher adherence v lower adherence	774952/1478	1.03 (0.94 to 1.14)	NA	NA	NA	NA	NA	critically low	-
Total protein	WCRF 2013	14	Dose-response	5% energy	1210245/1985	1.08 (0.99 to 1.19)	0.09981	0.00%	0.0003	NA	NA	low	NS
Animal protein	WCRF 2013	14	Dose-response	5% energy	1210245/1985	1.10 (1.00 to 1.20)	0.04936	0.00%	< 0.0001	NA	NA	low	IV
Plant protein	WCRF 2013	14	Dose-response	5% energy	1210245/1985	0.98 (0.80 to 1.22)	0.88739	0.00%	< 0.0001	NA	NA	low	NS
Dietary fiber	Xu 2019	7	High v low intake	higher adherence v lower adherence	942582/6590	0.82 (0.71 to 0.95)	0.007181	27.60%	0.0188	0.55 to 1.22	0.7645872	critically low	IV
**Micronutrients**													
Riboflavin	Clasen 2020	3	High v low intake	higher adherence v lower adherence	39789/1971	0.89 (0.70 to 1.13)	0.343247	0.00%	0.0068	0.14 to 5.84	0.6171367	low	NS
Vitamin B6	Clasen 2020	5	High v low intake	higher adherence v lower adherence	164883/2407	0.85 (0.70 to 1.04)	0.113403	0.00%	0.0055	0.58 to 1.27	0.5322223	low	NS
Folate	Clasen 2020	6	High v low intake	higher adherence v lower adherence	136319/3166	0.84 (0.69 to 1.03)	0.088191	6.00%	0.02	0.52 to 1.36	0.4896115	low	NS
Vitamin B12	Clasen 2020	3	High v low intake	higher adherence v lower adherence	127390/1578	1.13 (0.88 to 1.46)	0.331795	0.00%	0.0029	0.19 to 6.65	0.3081752	low	NS
Methionine	Clasen 2020	2	High v low intake	higher adherence v lower adherence	125094/436	1.29 (0.90 to 1.85)	0.170519	0.00%	0.0137	NA	NA	low	NS
Choline	Clasen 2020	2	High v low intake	higher adherence v lower adherence	125094/436	0.87 (0.63 to 1.22)	0.433326	0.00%	0.0035	NA	NA	low	NS
Betaine	Clasen 2020	2	High v low intake	higher adherence v	125094/436	1.01 (0.62 to 1.65)	0.962844	58.80%	0.0715	NA	NA	low	NS

				lower adherence									
Vitamin C	Jia 2015	10	High v low intake	higher adherence v lower adherence	274864/5182	0.77 (0.66 to 0.90)	0.000636	0.00%	0.0176	0.54 to 1.10	0.5218154	low	III
Vitamin D	Wu 2020	6	High v low intake	higher adherence v lower adherence	187560/NA	0.89 (0.71 to 1.11)	0.286865	36.50%	0.0365	0.48 to 1.64	0.5000861	moderate	NS
Dietary nitrate	Xie 2016	3	High v low intake	higher adherence v lower adherence	NA/NA	0.78 (0.40 to 1.55)	0.482996	89.20%	0.3018	0.0002 to 3013.70	0.7608724	critically low	NS
Dietary nitrite	Xie 2016	2	High v low intake	higher adherence v lower adherence	NA/NA	0.99 (0.81 to 1.21)	0.892113	0.00%	0.0061	NA	NA	critically low	NS
Vitamin E	Shen 2015	13	High v low intake	higher adherence v lower adherence	471006/6944	0.81 (0.69 to 0.94)	0.006361	49.20%	0.0347	0.52 to 1.26	0.856239	critically low	IV
Alpha-carotene	WCRF 2013	4	Dose-response	per 600 μg/d	284501/787	0.96 (0.86 to 1.07)	0.448428	33.10%	0.0043	0.32 to 2.86	0.9743947	low	NS
Beta-cryptoxanthin	WCRF 2013	4	Dose-response	per 100 μg/d	284501/787	0.97 (0.83 to 1.13)	0.700635	59.10%	0.0099	0.19 to 4.91	0.3298803	low	NS
Lutein and zeaxanthin	WCRF 2013	4	Dose-response	per 1000 μg/d	284501/787	0.99 (0.95 to 1.04)	0.827912	0.00%	< 0.0001	0.71 to 1.40	0.09332363	low	NS
Lycopene	WCRF 2013	4	Dose-response	per 4000 μg/d	284501/787	1.03 (0.73 to 1.47)	0.849859	5.90%	0.0386	0.04 to 30.00	0.4098994	low	NS
Total calcium (food and supplements)	WCRF 2013	3	Dose-response	per 200 mg/d	NA/1711	0.96 (0.94 to 0.99)	0.017337	0.00%	< 0.0001	0.78 to 1.19	0.4005484	low	IV
Dietary calcium	WCRF 2013	3	Dose-response	per 200 mg/d	NA/1574	0.98 (0.90 to 1.06)	0.614121	0.00%	0.002	0.45 to 2.12	0.147922	low	NS
Calcium (supplements)	WCRF 2013	3	High v low intake	higher adherence v lower adherence	NA/1482	0.80 (0.64 to 0.99)	0.039569	0.00%	0.0014	NA	NA	low	IV

*: summary hazard ratio extracted from published meta-analysis, no re-analysis possible; †: The association was not statistically significant after using the trim-and-fill method; #: SHR was generated by combining the hazard ratios from the 2 meta-analyses; SHR = summary hazard ratio; NA = not available; NS = not-significant; PI = prediction intervals; ASB = artificially sweetened beverages; SSB = sugar-sweetened beverages.

Table S9: Summary of results of the associations on various dietary factors and risk of RCC by study design

Exposure	No. of primary studies	No. of participants / No. of cases	Adjusted SHR (95%-CI)	p-value	I^2	95%-PI	SSE	LSS	ESB	CE
**Dietary patterns or diet quality indices**										
Healthy dietary pattern										
Overall	4	51709/1327	0.66 (0.51 to 0.85)	0.00145	19.40%	0.27 to 1.63	No	No	No	IV
Cc	3	5137/1234	0.62 (0.51 to 0.76)	1.8E-06	34.10%	0.03 to 13.85	No	No	No	III
Co	1	46572/93	0.81 (0.45 to 1.47)	-	-	-	-	-	-	-
Unhealthy/western pattern										
Overall	4	51709/1327	1.54 (1.19 to 2.01)	0.001232	24.70%	0.62 to 3.84	No	No	No	IV
Cc	3	5137/1234	1.61 (1.33 to 1.95)	9E-07	40.30%	0.09 to 26.81	No	No	No	III
Co	1	46572/93	1.14 (0.50 to 2.61)	-	-	-	-	-	-	-
Drinking pattern										
Overall	2	47705/554	0.69 (0.47 to 1.01)	0.057101	0.00%	NA	NA	No	No	NS
Cc	1	1133/461	0.65 (0.37 to 1.14)	-	-	-	-	-	-	-
Co	1	46572/93	0.72 (0.42 to 1.23)	-	-	-	-	-	-	-
Dietary Inflammatory Index										
Overall	2	36118/1030	1.08 (1.03 to 1.13)	0.00276	0.00%	NA	NA	No	No	IV
Co	1	33817/263	1.07 (0.99 to 1.15)	-	-	-	-	-	-	-
Cc	1	2301/767	1.08 (1.01 to 1.15)	-	-	-	-	-	-	-
Glycemic index										
Overall	5	502119/4281	1.18 (1.02 to 1.37)	0.026826	26.00%	0.79 to 1.75	No	No	No	IV
Cc	3	10794/2966	1.26 (1.10 to 1.45)	0.00114	0.00%	0.39 to 4.18	No	No	No	IV
Co	2	491325/1315	1.01 (0.85 to 1.21)	0.90898	0.00%	NA	NA	No	No	NS

Glycemic load										
Overall	5	502119/4281	1.16 (0.73 to 1.84)	0.522525	82.40%	0.21 to 6.45	No	No	No	NS
Cc	3	10794/2966	1.45 (0.86 to 2.46)	0.16306	87.60%	0.002 to 997.33	No	No	No	NS
Co	2	491325/1315	0.89 (0.66 to 1.21)	0.45501	47.70%	NA	NA	No	No	NS
**Foods**										
Fish										
Overall	15	608753/9324	0.97 (0.84 to 1.10)	0.612245	24.10%	0.63 to 1.47	No	No	No	NS
Cc	12	33532/6881	0.95 (0.86 to 1.05)	0.2913	0.00%	0.62 to 1.42	No	No	No	NS
Co	3	580730/2443	1.06 (0.78 to 1.46)	0.70129	61.60%	0.03 to 38.91	No	No	No	NS
All meat										
Overall (Cc)	11	15237/4835	1.27 (1.08 to 1.49)	0.003529	0.00%	0.83 to 1.94	No	No	No	IV
seafood*										
Overall (Co)	12	NA/1375	1.05 (0.82 to 1.35)	-	-	-	-	-	-	-
Vegetables										
Overall	16	1365634/9353	0.74 (0.63 to 0.86)	0.000073	51.30%	0.44 to 1.23	Yes	Yes	No	III
Co	3	1344053/3543	0.89 (0.80 to 0.99)	0.025432	42.30%	0.19 to 3.99	No	Yes	No	IV
Cc	13	21968/6197	0.70 (0.63 to 0.79)	9.18E-10	36.10%	0.40 to 1.22	No	Yes	No	II
Fruits										
Overall	17	1398778/9150	0.84 (0.69 to 1.02)	0.075352	50.90%	0.39 to 1.78	No	Yes	No	NS
Co	4	1378251/NA	0.92 (0.81 to 1.04)	0.166174	46.80%	0.07 to 9.64	No	Yes	No	NS
Cc	13	20527/5593	0.83 (0.67 to 1.03)	0.092835	54.60%	0.40 to 1.73	No	Yes	No	NS
Red meat										
Overall	19	1826171/12631	1.40 (1.15 to 1.70)	0.000807	71.40%	0.63 to 3.13	Yes	No	No	III
Co	5	1791359/4047	1.07 (0.96 to 1.19)	0.229841	1.50%	0.69 to 1.80	No	No	No	NS
Cc	14	34812/8584	1.50 (1.16 to 1.93)	0.001841	75.00%	0.57 to 3.92	Yes	No	No	IV

Processed meat										
Overall	19	1793675/14240	1.13 (1.01 to 1.26)	0.030876	46.80%	0.78 to 1.64	No	No	No	IV
Co	4	1757161/4033	1.11 (0.99 to 1.25)	0.076356	0.00%	0.71 to 1.76	No	No	No	NS
Cc	15	36514/10207	1.12 (0.98 to 1.28)	0.08463	57.00%	0.73 to 1.73	No	Yes	No	NS
Poultry										
Overall	16	NA/4165	1.23 (1.02 to 1.47)	0.030824	0.00%	0.88 to 1.70	No	No	No	IV
Co	11	682016/1348	1.25 (0.83 to 1.88)	-	-	-	-	-	-	-
Cc	5	10805/3168	1.22 (1.002 to 1.48)	0.047485	0.00%	0.79 to 1.88	No	No	No	IV
Cruciferous vegetables										
Overall	12	1228518/5773	0.81 (0.71 to 0.92)	0.001107	55.70%	0.54 to 1.20	No	Yes	No	IV
Co	6	1213193/1347	0.92 (0.84 to 1.003)	0.05848	37.40%	0.56 to 1.47	No	Yes	No	NS
Cc	6	15325/4426	0.72 (0.64 to 0.81)	1.8E-08	18.80%	0.48 to 1.09	No	No	No	III
Fruit and non-starchy vegetables										
Overall (Co)	7	756544/1215	0.99 (0.93 to 1.05)	0.748236	0.00%	0.85 to 1.15	Yes	No	No	NS
Non-starchy vegetables										
Overall (Co)	8	1248385/3031	0.99 (0.91 to 1.09)	0.942029	0.00%	0.80 to 1.24	No	No	No	NS
Tomatoes										
Overall (Co)	3	NA/427	1.11 (0.92 to 1.35)	0.28285	0.00%	0.27 to 4.51	No	No	No	NS
Citrus fruit										
Overall (Co)	7	NA/2735	0.98 (0.90 to 1.05)	0.53534	0.00%	0.80 to 1.19	No	Yes	No	NS
**Beverages**										
Sweetened carbonated beverage										
Overall	5	NA/NA	1.35 (0.86 to 2.11)	0.187219	60.10%	0.28 to 6.51	No	NA	No	NS
Cc	4	NA/NA	1.44 (0.81 to 2.56)	0.21376	64.00%	0.12 to 17.94	No	No	No	NS
Co	1	NA/NA	1.11 (0.89 to 1.38)	-	-	-	-	-	-	-

Sweet beverages<br>(include ASB and SSB)										
Overall	3	143194/1589	1.33 (0.74 to 2.40)	0.336717	85.60%	0.0008 to 2156.82	No	No	No	NS
Cc	2	6607/1341	1.61 (0.68 to 3.80)	0.276548	91.70%	NA	NA	No	No	NS
Co	1	136587/248	0.97 (0.84 to 1.12)	-	-	-	-	-	-	-
Tea										
Overall	12	1571852/6636	1.05 (0.83 to 1.33)	0.680841	71.50%	0.44 to 2.49	No	No	No	NS
Cc	10	17531/4570	1.09 (0.82 to 1.45)	0.565889	73.80%	0.40 to 2.95	No	No	No	NS
Co	2	1554321/2066	0.95 (0.81 to 1.11)	0.523378	70.20%	NA	NA	No	No	NS
Coffee										
Overall	22	1991863/NA	0.99 (0.84 to 1.18)	0.949021	32.60%	0.50 to 1.98	No	No	No	NS
Cc	16	24501/8342	1.08 (0.97 to 1.19)	0.159532	3.70%	0.63 to 1.73	No	Yes	No	NS
Co	6	1967362/NA	0.88 (0.54 to 1.43)	0.603877	50.90%	0.17 to 4.57	No	No	No	NS
**Alcoholic beverages**										
Beer										
Overall (Cc)	8	NA/5538	0.84 (0.70 to 1.002)	0.052184	14.80%	0.52 to 1.34	No	No	No	NS
Wine										
Overall (Cc)	8	NA/5538	0.70 (0.52 to 0.94)	0.019297	56.00%	0.29 to 1.70	No	Yes	No	IV
Spirits										
Overall (Cc)	9	NA/6393	0.80 (0.64 to 0.99)	0.048382	74.30%	0.39 to 1.62	No	Yes	No	IV
Alcohol (light)										
Overall (Co)	6	3720850/5214	0.91 (0.81 to 1.02)	0.102662	39.60%	0.64 to 1.28	No	No	No	NS
Alcohol (moderate)										

Overall (Co)	8	4139301/5364	0.77 (0.58 to 1.02)	0.069063	29.20%	0.30 to 1.98	No	Yes	No	NS
Alcohol (heavy)										
Overall (Co)	3	2065995/363	0.93 (0.27 to 3.23)	0.9134	81.20%	0.0000 to 3904180.81	No	Yes	Yes	NS
Alcohol (any)										
Overall (Co)	8	4867646/5503	0.88 (0.63 to 1.22)	0.439292	62.90%	0.27 to 2.81	No	Yes	Yes	NS
**Macronutrients**										
Fruit fiber										
Overall	3	496292/2906	0.91 (0.74 to 1.11)	0.346662	0.00%	0.14 to 6.06	No	No	No	NS
Co	1	491841/1816	0.91 (0.77 to 1.07)	-	-	-	-	-	-	-
Cc	2	4451/1090	0.93 (0.72 to 1.18)	0.53798	34.90%	NA	NA	No	No	NS
Vegetable fiber										
Overall	3	496292/2906	0.68 (0.44 to 1.06)	0.089923	76.90%	0.003 to 139.60	Yes	No	No	NS
Co	1	491841/1816	0.90 (0.78 to 1.04)	-	-	-	-	-	-	-
Cc	2	4451/1090	0.57 (0.33 to 0.99)	0.04713	72.10%	NA	NA	Yes	No	IV
Cereal fiber										
Overall	3	496292/2906	1.05 (0.88 to 1.26)	0.569512	0.00%	0.22 to 4.94	No	No	No	NS
Co	1	491841/1816	0.99 (0.85 to 1.16)	-	-	-	-	-	-	-
Cc	2	4451/1090	1.17 (0.90 to 1.52)	0.23751	0.00%	NA	NA	No	No	NS
Legume fiber*										
Overall (Co)	1	491841/1816	0.80 (0.69 to 0.93)	-	-	-	-	-	-	-
Total fat										
Overall (Co)	14	1210245/1985	1.02 (0.98 to 1.07)	0.36075	0.00%	NA	NA	No	No	NS
Saturated fat										

Overall (Co)	14	1210245/1985	1.05 (0.88 to 1.24)	0.60759	41.30%	NA	NA	No	No	NS
Monounsaturated fat										
Overall (Co)	14	1210245/1985	0.98 (0.83 to 1.15)	0.76247	0.00%	NA	NA	No	No	NS
Polyunsaturated fat										
Overall (Co)	14	1210245/1985	0.90 (0.75 to 1.07)	0.21982	18.90%	NA	NA	No	No	NS
Animal fat*										
Overall (Co)	8	417093/900	1.01 (0.96 to 1.07)	-	-	-	-	-	-	-
Plant fat*										
Overall (Co)	8	417093/900	0.99 (0.93 to 1.05)	-	-	-	-	-	-	-
Cholesterol*										
Overall (Co)	13	774952/1478	1.03 (0.94 to 1.14)	-	-	-	-	-	-	-
Total protein										
Overall (Co)	14	1210245/1985	1.08 (0.99 to 1.19)	0.09981	0.00%	NA	NA	No	No	NS
Animal protein										
Overall (Co)	14	1210245/1985	1.10 (1.0003 to 1.20)	0.04936	0.00%	NA	NA	No	No	IV
Plant protein										
Overall (Co)	14	1210245/1985	0.98 (0.80 to 1.22)	0.88739	0.00%	NA	NA	No	No	NS
Dietary fiber										
Overall	7	942582/6590	0.82 (0.71 to 0.95)	0.007181	27.60%	0.55 to 1.22	No	Yes	No	IV
Co	2	927134/2323	0.85 (0.73 to 0.98)	0.024525	41.60%	NA	NA	Yes	No	IV
Cc	5	15448/4267	0.79 (0.69 to 0.90)	0.000402	34.50%	0.47 to 1.33	No	Yes	No	III
**Micronutrients**										
Riboflavin										
Overall	3	39789/1971	0.89 (0.70 to 1.13)	0.343247	0.00%	0.14 to 5.84	No	No	No	NS
Co	1	35192/62	0.71 (0.39 to 1.30)	-	-	-	-	-	-	-

Cc	2	4597/1909	0.92 (0.73 to 1.17)	0.50215	0.00%	NA	NA	No	No	NS
Vitamin B6	5									
Overall	5	164883/2407	0.85 (0.70 to 1.04)	0.113403	0.00%	0.58 to 1.27	No	No	No	NS
Cc	2	4597/1909	0.81 (0.64 to 1.03)	0.08175	0.00%	NA	NA	No	No	NS
Co	3	160286/498	0.92 (0.70 to 1.21)	0.53487	0.00%	0.13 to 6.35	No	No	No	NS
Folate										
Overall	6	136319/3166	0.84 (0.69 to 1.03)	0.088191	6.00%	0.52 to 1.36	No	No	No	NS
Cc	3	6747/2232	0.77 (0.63 to 0.95)	0.01457	0.00%	0.08 to 7.29	No	No	No	IV
Co	3	129074/934	0.97 (0.76 to 1.23)	0.78937	0.00%	0.11 to 8.11	Yes	No	No	NS
Vitamin B12										
Overall	3	127390/1578	1.13 (0.88 to 1.46)	0.331795	0.00%	0.19 to 6.65	No	No	No	NS
Cc	1	2296/1142	1.00 (0.68 to 1.46)	-	-	-	-	-	-	-
Co	2	125094/436	1.24 (0.90 to 1.70)	0.19407	0.00%	NA	NA	No	No	NS
Methionine										
Overall (Co)	2	125094/436	1.29 (0.90 to 1.85)	0.170519	0.00%	NA	NA	No	No	NS
Choline										
Overall (Co)	2	125094/436	0.87 (0.63 to 1.22)	0.433326	0.00%	NA	NA	No	No	NS
Betaine										
Overall (Co)	2	125094/436	1.01 (0.62 to 1.65)	0.962844	58.80%	NA	NA	No	No	NS
Vitamin C										
Overall	10	274864/5182	0.77 (0.66 to 0.90)	0.000636	0.00%	0.54 to 1.10	No	No	No	III
Cc	7	15019/4439	0.75 (0.66 to 0.86)	1.3E-05	0.00%	0.59 to 0.95	Yes	No	No	III
Co	3	259845/743	0.91 (0.69 to 1.20)	0.48914	34.00%	0.01 to 52.50	No	No	No	NS
Vitamin D										
Overall	6	187560/NA	0.89 (0.71 to 1.11)	0.286865	36.50%	0.49 to 1.64	No	No	No	NS

Co	3	182756/NA	0.99 (0.66 to 1.50)	0.98827	66.60%	0.01 to 90.98	No	No	No	NS
Cc	3	4804/1895	0.79 (0.65 to 0.97)	0.02623	0.00%	0.21 to 3.07	No	No	No	IV
Dietary nitrate										
Overall	3	NA/NA	0.78 (0.40 to 1.55)	0.482996	89.20%	0.0002 to 3013.70	No	NA	No	NS
Co	2	NA/NA	0.99 (0.85 to 1.15)	0.91048	0.00%	NA	NA	No	No	NS
Cc	1	NA/NA	0.41 (0.28 to 0.60)	-	-	-	-	-	-	-
Dietary nitrite										
Overall	2	NA/NA	0.99 (0.81 to 1.21)	0.892113	0.00%	NA	NA	No	No	NS
Cc	1	NA/NA	0.82 (0.50 to 1.34)	-	-	-	-	-	-	-
Co	1	NA/NA	1.02 (0.87 to 1.19)	-	-	-	-	-	-	-
Vitamin E										
Overall	13	471006/6944	0.81 (0.69 to 0.94)	0.006361	49.20%	0.52 to 1.26	No	No	No	IV
Cc	7	20543/5731	0.78 (0.63 to 0.96)	0.0186	67.50%	0.42 to 1.44	No	Yes	No	IV
Co	6	450526/1213	0.88 (0.72 to 1.08)	0.226063	0.00%	0.50 to 1.50	Yes	No	No	NS
Alpha-carotene										
Overall (Co)	4	284501/787	0.96 (0.86 to 1.07)	0.448428	33.10%	0.32 to 2.86	No	No	No	NS
Beta-cryptoxanthin										
Overall (Co)	4	284501/787	0.97 (0.83 to 1.13)	0.700635	59.10%	0.19 to 4.91	No	Yes	No	NS
Lutein and zeaxanthin										
Overall (Co)	4	284501/787	0.99 (0.95 to 1.04)	0.827912	0.00%	0.71 to 1.40	Yes	No	No	NS
Lycopene										
Overall (Co)	4	284501/787	1.03 (0.73 to 1.47)	0.849859	5.90%	0.04 to 30.00	No	No	No	NS
Total calcium (food and supplements)										
Overall (Co)	3	NA/1711	0.96 (0.94 to 0.99)	0.017337	0.00%	0.78 to 1.19	No	Yes	No	IV

Dietary calcium										
Overall (Co)	3	NA/1574	0.98 (0.90 to 1.06)	0.614121	0.00%	0.45 to 2.12	No	No	No	NS
Calcium from supplements										
Overall (Co)	3	NA/1482	0.80 (0.64 to 0.99)	0.039569	0.00%	NA	NA	No	No	IV

CE = class of evidence; CI = confidence interval; PI = prediction interval; SSE = small study effect; ESB = excess significance bias; LSS = Largest study significant; SHR = summary hazard ratio; CC = case-control studies; Co = cohort studies; NA = not available; NS = not-significant; ASB = artificially sweetened beverages; SSB = sugar-sweetened beverages; *: summary hazard ratio extracted from published meta-analysis, no re-analysis possible; -: Results could not be calculated because of insufficient number of studies.

Table S10: Summary of results of the associations on various dietary factors and risk of RCC by region

Exposure	No. of primary studies	No. of participants / No. of cases	Adjusted SHR (95% CI)	p-value	I^2	95%-PI	SSE	LSS	ESB	CE
**Dietary patterns or diet quality indices**										
Healthy dietary pattern										
Overall	4	51709/1327	0.66 (0.51 to 0.85)	0.001449	19.40%	0.27 to 1.63	No	No	No	IV
America	3	5137/1234	0.62 (0.51 to 0.76)	0.000002	34.10%	0.03 to 13.85	No	No	No	III
Europe	1	46572/93	0.81 (0.45 to 1.47)	-	-	-	-	-	-	-
Unhealthy/western pattern										
Overall	4	51709/1327	1.54 (1.19 to 2.01)	0.001232	24.70%	0.62 to 3.84	No	No	No	IV
America	3	5137/1234	1.61 (1.33 to 1.95)	0.000000902	40.30%	0.09 to 26.81	No	No	No	III
Europe	1	46572/93	1.14 (0.50 to 2.61)	-	-	-	-	-	-	-
Drinking pattern										
Overall	2	47705/554	0.69 (0.47 to 1.01)	0.057101	0.00%	NA	NA	No	No	NS
North America	1	1133/461	0.65 (0.37 to 1.14)	-	-	-	-	-	-	-
Europe	1	46572/93	0.72 (0.42 to 1.23)	-	-	-	-	-	-	-
Dietary Inflammatory Index										
Overall	2	36118/1030	1.08 (1.03 to 1.13)	0.00276	0.00%	NA	NA	No	No	IV
North America	1	33817/263	1.07 (0.99 to 1.15)	-	-	-	-	-	-	-
Europe	1	2301/767	1.08 (1.01 to 1.15)	-	-	-	-	-	-	-
Glycemic index										
Overall	5	502119/4281	1.18 (1.02 to 1.37)	0.026826	26.00%	0.80 to 1.75	No	No	No	IV
North America	3	454670/3378	1.12 (0.99 to 1.26)	0.066159	39.70%	0.20 to 6.45	No	No	No	NS

Europe	2	47449/903	1.36 (1.04 to 1.77)	0.022337	0.00%	NA	NA	No	No	IV
Glycemic load										
Overall	5	502119/4281	1.16 (0.73 to 1.84)	0.522525	82.40%	0.21 to 6.45	No	No	No	NS
North America	3	454670/3378	1.08 (0.93 to 1.27)	0.317567	0.00%	0.35 to 3.35	No	No	No	NS
Europe	2	47449/903	1.22 (0.28 to 5.37)	0.788034	92.20%	NA	NA	No	No	NS
**Foods**										
Fish										
Overall	15	608753/9324	0.97 (0.84 to 1.10)	0.612245	24.10%	0.63 to 1.47	No	No	No	NS
North America	4	502412/4338	1.06 (0.94 to 1.19)	0.353153	0.00%	0.46 to 2.41	No	No	No	NS
Europe	9	107489/3647	0.98 (0.86 to 1.10)	0.703011	9.20%	0.69 to 1.37	No	No	No	NS
Australia	1	3896/1185	0.89 (0.70 to 1.13)	-	-	-	-	-	-	-
Asia	1	465/154	0.48 (0.25 to 0.93)	-	-	-	-	-	-	-
All meat										
Overall	11	15237/4835	1.27 (1.08 to 1.49)	0.003529	0.00%	0.83 to 1.94	No	No	No	IV
Europe	5	3579/1385	1.12 (0.90 to 1.40)	0.2971	0.00%	0.64 to 2.01	No	No	No	NS
North America	5	11347/3296	1.27 (1.13 to 1.43)	0.000073	0.00%	0.88 to 1.87	No	No	No	III
Asia	1	311/154	2.34 (1.10 to 5.00)	-	-	-	-	-	-	-
Seafood*										
Overall	12	NA/1375	1.05 (0.82 to 1.35)	-	-	-	-	-	-	-
Vegetables										
Overall	16	1365634/9353	0.74 (0.63 to 0.86)	0.000073	51.30%	0.44 to 1.23	Yes	Yes	No	III
America	6	573684/3534	0.71 (0.53 to 0.95)	0.022256	69.20%	0.29 to 1.77	Yes	No	No	IV
International	2	777663/2663	0.80 (0.69 to 0.93)	0.003834	0.00%	NA	NA	Yes	No	IV
Europe	7	14363/3389	0.70 (0.60 to 0.81)	0.0000013	35.10%	0.36 to 1.46	No	Yes	No	III
Asia	1	311/154	0.65 (0.32 to 1.31)	-	-	-	-	-	-	-

Fruits										
Overall	17	1398778/9150	0.84 (0.69 to 1.02)	0.075352	50.90%	0.39 to 1.78	No	Yes	No	NS
America	8	609015/NA	0.99 (0.89 to 1.12)	0.995691	42.30%	0.32 to 3.02	No	No	No	NS
International	2	777663/2663	0.82 (0.69 to 0.97)	0.018398	0.00%	NA	NA	Yes	No	IV
Europe	6	11789/2324	0.72 (0.54 to 0.95)	0.019378	56.90%	0.31 to 1.66	Yes	Yes	No	IV
Asia	1	311/154	0.51 (0.19 to 1.37)	-	-	-	-	-	-	-
Red meat										
Overall	19	1826171/12631	1.40 (1.15 to 1.70)	0.000807	71.40%	0.63 to 3.13	Yes	No	No	III
Europe	4	388906/2713	1.20 (0.84 to 1.72)	0.313392	62.10%	0.27 to 5.34	No	No	No	NS
America	12	545085/7207	1.61 (1.24 to 2.10)	0.000337	72.20%	0.65 to 4.00	Yes	No	No	III
International	2	777663/2663	0.98 (0.86 to 1.11)	0.714192	0.00%	NA	NA	No	No	NS
Asia	1	114517/48	1.30 (0.69 to 2.45)	-	-	-	-	-	-	-
Processed meat										
Overall	19	1793675/14240	1.13 (1.01 to 1.26)	0.030876	46.80%	0.78 to 1.64	No	No	No	IV
Europe	4	381631/2763	0.99 (0.73 to 1.36)	0.977711	65.80%	0.27 to 3.69	Yes	No	Yes	NS
America	12	519864/8766	1.19 (1.11 to 1.28)	0.0000008 16	37.90%	0.87 to 1.65	No	No	No	III
International	2	777663/2663	1.02 (0.87 to 1.18)	0.830474	0.00%	NA	NA	No	No	NS
Asia	1	114517/48	1.60 (0.58 to 4.44)	-	-	-	-	-	-	-
Poultry										
Overall	16	NA/4165	1.23 (1.02 to 1.47)	0.030824	0.00%	0.88 to 1.70	No	No	No	IV
International	12	682016/1348	1.25 (0.83 to 1.88)	-	-	-	-	-	-	-
North America	4	10076/2789	1.17 (0.94 to 1.44)	0.153997	0.00%	0.67 to 2.02	No	No	No	NS
Europe	1	729/379	1.52 (0.94 to 2.47)	-	-	-	-	-	-	-
Cruciferous vegetables										

Overall	12	1228518/5773	0.81 (0.71 to 0.92)	0.001107	55.70%	0.54 to 1.20	No	Yes	No	IV
North America	6	640450/3557	0.78 (0.70 to 0.86)	0.0000006 8	27.30%	0.51 to 1.13	No	Yes	No	II
Europe	6	588068/2216	0.87 (0.71 to 1.07)	0.181607	63.20%	0.48 to 1.58	No	No	No	NS
Fruit and non-starchy vegetables										
Overall	7	756544/1215	0.99 (0.93 to 1.05)	0.748236	0.00%	0.85 to 1.15	Yes	No	No	NS
Europe	4	584765/958	0.99 (0.94 to 1.05)	0.760772	0.00%	0.86 to 1.14	No	No	No	NS
USA	2	171779/310	1.02 (0.85 to 1.23)	0.853945	70.70%	NA	NA	No	No	NS
Non-starchy vegetables										
Overall	8	1248385/3031	0.99 (0.91 to 1.09)	0.942029	0.00%	0.80 to 1.24	No	No	No	NS
USA	3	663620/2126	0.99 (0.96 to 1.02)	0.511724	44.30%	0.19 to 5.19	No	No	No	NS
Europe	4	584765/958	1.01 (0.90 to 1.14)	0.83671	0.00%	0.70 to 1.46	No	No	No	NS
Tomatoes										
Overall	3	NA/427	1.11 (0.92 to 1.35)	0.28285	0.00%	0.27 to 4.51	No	No	No	NS
Asia	1	NA/56	1.19 (0.43 to 3.32)	-	-	-	-	-	-	-
Europe	2	181852/397	1.11 (0.92 to 1.33)	0.278029	0.00%	NA	NA	No	No	NS
Citrus fruit										
Overall	7	NA/2735	0.98 (0.90 to 1.05)	0.53534	0.00%	0.80 to 1.19	No	Yes	No	NS
USA	2	628428/2064	0.96 (0.93 to 0.99)	0.025052	0.00%	NA	NA	Yes	No	IV
Europe	3	208914/652	1.01 (0.93 to 1.10)	0.785659	0.00%	0.42 to 2.40	No	No	No	NS
Asia	1	NA/62	0.65 (0.18 to 2.38)	-	-	-	-	-	-	-
**Beverages**										
Sweetened carbonated beverage										
Overall	5	NA/NA	1.35 (0.86 to 2.11)	0.187219	60.10%	0.28 to 6.51	No	NA	No	NS
North America	3	NA/NA	1.64 (0.92 to 2.92)	0.091794	70.20%	0.002 to 1575.77	No	No	No	NS

Europe	1	NA/NA	0.72 (0.27 to 1.94)	-	-	-	-	-	-	-
International	1	NA/NA	1.11 (0.89 to 1.38)	-	-	-	-	-	-	-
Sweet beverages (include ASB and SSB)										
Overall (North America)	3	143194/1589	1.33 (0.74 to 2.40)	0.336717	85.60%	0.0008 to 2156.82	No	No	No	NS
Tea										
Overall	12	1571852/6636	1.05 (0.83 to 1.33)	0.680841	71.50%	0.44 to 2.49	No	No	No	NS
America	6	12994/2945	1.29 (1.09 to 1.53)	0.003777	0.00%	0.90 to 1.87	No	No	No	IV
Europe	4	783357/1963	1.01 (0.98 to 1.05)	0.57474	43.90%	0.45 to 2.33	No	No	No	NS
Asia	1	549/250	0.34 (0.21 to 0.55)	-	-	-	-	-	-	-
International	1	774952/1478	0.85 (0.71 to 1.02)	-	-	-	-	-	-	-
Coffee										
Overall	22	1991863/NA	0.99 (0.84 to 1.18)	0.949021	32.60%	0.50 to 1.98	No	No	No	NS
Europe	9	851796/NA	0.97 (0.84 to 1.12)	0.651703	15.40%	0.43 to 1.96	Yes	No	No	NS
North America	11	1022839/NA	1.01 (0.91 to 1.12)	0.879245	45.10%	0.53 to 1.92	No	No	No	NS
International	1	2711/1186	1.22 (0.42 to 3.52)	-	-	-	-	-	-	-
Asia	1	114517/NA	2.69 (0.89 to 8.12)	-	-	-	-	-	-	-
**Alcoholic beverages**										
Beer										
Overall	8	NA/5538	0.84 (0.7 to 1.002)	0.052184	14.80%	0.52 to 1.34	No	No	No	NS
North America	5	NA/2173	0.79 (0.68 to 0.93)	0.004985	48.80%	0.35 to 2.10	Yes	Yes	No	IV
International	1	NA/1185	0.88 (0.63 to 1.23)		-	-	-	-	-	-
Europe	2	NA/2180	0.83 (0.68 to 1.01)	0.067648	0.00%	NA	NA	No	No	NS
Wine										

Overall	8	NA/5538	0.70 (0.52 to 0.94)	0.019297	56.00%	0.29 to 1.70	No	Yes	No	IV
North America	5	NA/2173	0.78 (0.64 to 0.94)	0.009045	0.00%	0.47 to 1.33	No	Yes	No	IV
International	1	NA/1185	0.33 (0.20 to 0.53)		-	-	-	-	-	-
Europe	2	NA/2180	0.71 (0.35 to 1.44)	0.343396	58.90%	NA	NA	Yes	No	NS
Spirits										
Overall	9	NA/6393	0.80 (0.64 to 0.99)	0.048382	74.30%	0.39 to 1.62	No	Yes	No	IV
North America	5	NA/2173	0.90 (0.77 to 1.06)	0.202098	14.00%	0.50 to 1.69	No	No	No	NS
International	1	NA/1185	0.46 (0.37 to 0.58)	-	-	-	-	-	-	-
Europe	3	NA/3035	0.84 (0.71 to 0.99)	0.040466	21.30%	0.03 to 24.37	No	No	No	IV
Alcohol(light)										
Overall	6	3720850/5214	0.91 (0.81 to 1.02)	0.102662	39.60%	0.64 to 1.28	No	No	No	NS
Europe/North America	5	2801651/5140	0.94 (0.88 to 1.002)	0.059057	38.40%	0.70 to 1.22	No	No	No	NS
Asia	1	919199/74	0.63 (0.35 to 1.13)	-	-	-	-	-	-	-
Alcohol(moderate)										
Overall	8	4139301/5364	0.77 (0.58 to 1.02)	0.069063	29.20%	0.30 to 1.98	No	Yes	No	NS
Europe/North America	6	2792947/5262	0.77 (0.70 to 0.84)	2.20E-09	0.00%	0.61 to 0.97	No	Yes	No	I
Asia	2	1346354/102	1.06 (0.19 to 5.90)	0.946316	84.30%	NA	NA	Yes	Yes	NS
Alcohol(heavy)										
Overall	3	2065995/363	0.93 (0.27 to 3.23)	0.9134	81.20%	0.0000 to 3904180.81	No	Yes	Yes	NS
North America	1	77260/249	0.69 (0.43 to 1.11)	-	-	-	-	-	-	-
Asia	2	1988735/114	1.16 (0.14 to 9.43)	0.892975	90.60%	NA	NA	Yes	Yes	NS
Alcohol(any)										
Overall	8	4867646/5503	0.88 (0.63 to 1.22)	0.439292	62.90%	0.27 to 2.81	No	Yes	Yes	NS
Europe/North America	6	2878911/5389	0.89 (0.83 to 0.94)	0.000181	12.10%	0.72 to 1.07	No	Yes	Yes	III

Asia	2	1988735/114	1.29 (0.19 to 8.66)	0.796419	91.60%	NA	NA	Yes	Yes	NS
**Macronutrients**										
Fruit fiber										
Overall	3	496292/2906	0.91 (0.74 to 1.11)	0.346662	0.00%	0.14 to 6.06	No	No	No	NS
North America	2	493991/2139	0.89 (0.76 to 1.04)	0.134649	0.00%	NA	NA	No	No	NS
Europe	1	2301/767	1.01 (0.76 to 1.34)	-	-	-	-	-	-	-
Vegetable fiber										
Overall	3	496292/2906	0.68 (0.44 to 1.06)	0.089923	76.90%	0.003 to 139.60	Yes	No	No	NS
North America	2	493991/2139	0.63 (0.30 to 1.34)	0.232265	87.20%	NA	NA	No	No	NS
Europe	1	2301/767	0.73 (0.54 to 0.98)	-	-	-	-	-	-	-
Cereal fiber										
Overall	3	496292/2906	1.05 (0.88 to 1.26)	0.569512	0.00%	0.22 to 4.94	No	No	No	NS
North America	2	493991/2139	0.99 (0.85 to 1.15)	0.905557	0.00%	NA	NA	No	No	NS
Europe	1	2301/767	1.26 (0.92 to 1.73)	-	-	-	-	-	-	-
Legume fiber*										
Overall	1	491841/1816	0.80 (0.69 to 0.93)	-	-	-	-	-	-	-
Total fat										
Overall	14	1210245/1985	1.02 (0.98 to 1.07)	0.360753	0.00%	NA	NA	No	No	NS
International	13	774952/1478	1.02 (0.98 to 1.07)	-	-	-	-	-	-	-
Europe	1	435293/507	1.05 (0.78 to 1.42)	-	-	-	-	-	-	-
Saturated fat										
Overall	14	1210245/1985	1.05 (0.88 to 1.24)	0.60759	41.30%	NA	NA	No	No	NS
International	13	774952/1478	0.98 (0.85 to 1.12)	-	-	-	-	-	-	-
Europe	1	435293/507	1.17 (0.93 to 1.47)	-	-	-	-	-	-	-
Monounsaturated fat										

Overall	14	1210245/1985	0.98 (0.83 to 1.15)	0.762472	0.00%	NA	NA	No	No	NS
International	13	774952/1478	0.95 (0.82 to 1.11)	-	-	-	-	-	-	-
Europe	1	435293/507	1.10 (0.76 to 1.59)	-	-	-	-	-	-	-
Polyunsaturated fat										
Overall	14	1210245/1985	0.90 (0.75 to 1.07)	0.219824	18.90%	NA	NA	No	No	NS
International	13	774952/1478	0.95 (0.82 to 1.11)	-	-	-	-	-	-	-
Europe	1	435293/507	0.80 (0.62 to 1.04)	-	-	-	-	-	-	-
Animal fat*										
Overall (Europe/North America)	8	417093/900	1.01 (0.96 to 1.07)	-	-	-	-	-	-	-
Plant fat*										
Overall (Europe/North America)	8	417093/900	0.99 (0.93 to 1.05)	-	-	-	-	-	-	-
Cholesterol*										
Overall (International)	13	774952/1478	1.03 (0.94 to 1.14)	-	-	-	-	-	-	-
Total protein										
Overall	14	1210245/1985	1.08 (0.99 to 1.19)	0.099809	0.00%	NA	NA	No	No	NS
International	13	774952/1478	1.07 (0.97 to 1.18)	-	-	-	-	-	-	-
Europe	1	435293/507	1.15 (0.90 to 1.47)	-	-	-	-	-	-	-
Animal protein										
Overall	14	1210245/1985	1.10 (1.0003 to 1.2)	0.049362	0.00%	NA	NA	No	No	IV
International	13	774952/1478	1.09 (0.98 to 1.21)	-	-	-	-	-	-	-
Europe	1	435293/507	1.12 (0.93 to 1.35)	-	-	-	-	-	-	-
Plant protein										
Overall	14	1210245/1985	0.98 (0.80 to 1.22)	0.887389	0.00%	NA	NA	No	No	NS
International	13	774952/1478	0.99 (0.73 to 1.34)	-	-	-	-	-	-	-
Europe	1	435293/507	0.98 (0.73 to 1.32)	-	-	-	-	-	-	-

Dietary fiber										
Overall	7	942582/6590	0.82 (0.71 to 0.95)	0.007181	27.60%	0.55 to 1.22	No	Yes	No	IV
Europe	2	437594/1274	1.05 (0.83 to 1.32)	0.690911	0.00%	NA	NA	No	No	NS
North America	4	502277/4131	0.75 (0.67 to 0.85)	0.000002	0.00%	0.54 to 1.05	Yes	Yes	Yes	III
International	1	2711/1185	0.87 (0.65 to 1.16)	-	-	-	-	-	-	-
**Micronutrients**										
Riboflavin										
Overall	3	39789/1971	0.89 (0.70 to 1.13)	0.343247	0.00%	0.14 to 5.84	No	No	No	NS
North America	2	37488/1204	0.91 (0.66 to 1.25)	0.552379	0.00%	NA	NA	No	No	NS
Europe	1	2301/767	0.88 (0.66 to 1.18)	-	-	-	-	-	-	-
Vitamin B6	5									
Overall	5	164883/2407	0.85 (0.70 to 1.04)	0.113403	0.00%	0.58 to 1.27	No	No	No	NS
North America	4	162582/1640	0.86 (0.68 to 1.08)	0.192192	0.00%	0.43 to 1.70	No	No	No	NS
Europe	1	2301/767	0.85 (0.64 to 1.13)	-	-	-	-	-	-	-
Folate										
Overall	6	136319/3166	0.84 (0.69 to 1.03)	0.088191	6.00%	0.52 to 1.36	No	No	No	NS
North America	4	129540/1901	0.75 (0.60 to 0.95)	0.014541	0.00%	0.39 to 1.47	No	No	No	IV
Europe	2	6281/1265	0.95 (0.76 to 1.18)	0.644874	35.60%	NA	NA	No	No	NS
Vitamin B12										
Overall (North America)	3	127390/1578	1.13 (0.88 to 1.46)	0.331795	0.00%	0.19 to 6.65	No	No	No	NS
Methionine										
Overall (North America)	2	125094/436	1.29 (0.90 to 1.85)	0.170519	0.00%	NA	NA	No	No	NS
Choline										
Overall (North America)	2	125094/436	0.87 (0.63 to 1.22)	0.433326	0.00%	NA	NA	No	No	NS
Betaine										

Overall (North America)	2	125094/436	1.01 (0.62 to 1.65)	0.962844	58.80%	NA	NA	No	No	NS
Vitamin C										
Overall	10	274864/5182	0.77 (0.66 to 0.90)	0.000636	0.00%	0.54 to 1.10	No	No	No	III
North America	6	270580/3408	0.82 (0.71 to 0.94)	0.005387	0.00%	0.49 to 1.36	No	No	No	IV
Europe	4	4284/1774	0.70 (0.57 to 0.86)	0.000567	0.00%	0.41 to 1.18	No	Yes	No	III
Vitamin D										
Overall	6	187560/NA	0.89 (0.71 to 1.11)	0.286865	36.50%	0.48 to 1.64	No	No	No	NS
North America	2	153623/NA	0.81 (0.61 to 1.06)	0.121077	0.00%	NA	NA	No	No	NS
Europe	4	33937/NA	0.93 (0.68 to 1.27)	0.654477	59.80%	0.26 to 3.35	No	No	No	NS
Dietary nitrate										
Overall (North America)	3	NA/NA	0.78 (0.40 to 1.55)	0.482996	89.20%	0.0002 to 3013.70	No	NA	No	NS
Dietary nitrite										
Overall (North America)	2	NA/NA	0.99 (0.81 to 1.21)	0.892113	0.00%	NA	NA	No	No	NS
Vitamin E										
Overall	13	471006/6944	0.81 (0.69 to 0.94)	0.006361	49.20%	0.52 to 1.26	No	No	No	IV
North America	7	316723/3723	0.82 (0.67 to 1.02)	0.068931	53.00%	0.48 to 1.41	No	No	No	NS
Europe	5	151635/2036	0.77 (0.58 to 1.01)	0.062495	58.00%	0.32 to 1.82	No	No	No	NS
International	1	2711/1185	0.90 (0.69 to 1.18)	-	-	-	-	-	-	-
Alpha-carotene										
Overall	4	284501/787	0.96 (0.86 to 1.07)	0.448428	33.10%	0.32 to 2.86	No	No	No	NS
Europe	2	147914/539	0.99 (0.90 to 1.08)	0.79312	34.30%	NA	NA	No	No	NS
USA	1	136587/248	0.88 (0.76 to 1.03)	-	-	-	-	-	-	-
Beta-cryptoxanthin										
Overall	4	284501/787	0.97 (0.83 to 1.13)	0.700635	59.10%	0.19 to 4.91	No	Yes	No	NS

Europe	2	147914/539	1.06 (0.99 to 1.12)	0.05436	0.00%	NA	NA	Yes	No	NS
USA	1	136587/248	0.83 (0.66 to 1.04)	-	-	-	-	-	-	-
Lutein and zeaxanthin										
Overall	4	284501/787	0.99 (0.95 to 1.04)	0.827912	0.00%	0.71 to 1.40	Yes	No	No	NS
Europe	2	147914/539	1.02 (0.93 to 1.11)	0.727552	0.00%	NA	NA	No	No	NS
USA	1	136587/248	0.99 (0.93 to 1.04)	-	-	-	-	-	-	-
Lycopene										
Overall	4	284501/787	1.03 (0.73 to 1.47)	0.849859	5.90%	0.04 to 30.00	No	No	No	NS
Europe	2	147914/539	1.46 (0.75 to 2.84)	0.260221	0.00%	NA	NA	No	No	NS
USA	1	136587/248	0.93 (0.84 to 1.03)	-	-	-	-	-	-	-
Total calcium (food and supplements)										
Overall	3	NA/1711	0.96 (0.94 to 0.99)	0.017337	0.00%	0.78 to 1.19	No	Yes	No	IV
USA	2	NA/1482	0.97 (0.94 to 0.99)	0.016165	0.00%	NA	NA	NA	No	IV
Europe	1	27111/229	0.95 (0.83 to 1.09)	-	-	-	-	-	-	-
Dietary calcium										
Overall	3	NA/1574	0.98 (0.90 to 1.06)	0.614121	0.00%	0.45 to 2.11	No	No	No	NS
Europe	1	30000/154	1.00 (0.92 to 1.09)	-	-	-	-	-	-	-
USA	2	NA/1420	0.97 (0.89 to 1.06)	0.50852	31.70%	NA	NA	NA	No	NS
Calcium from supplements										
Overall (USA)	3	NA/1482	0.80 (0.64 to 0.99)	0.039569	0.00%	NA	NA	No	No	IV

CE = class of evidence; CI = confidence interval; PI = prediction interval; SSE = small study effect; ESB = excess significance bias; LSS - Largest study significant; SHR = summary hazard ratio; International = Europe/North America/Australia; NA = not available; NS = not-significant; ASB = artificially sweetened beverages; SSB = sugar-sweetened beverages; *: summary hazard ratio extracted from published meta-analysis, no re-analysis possible; -: Results could not be calculated because of insufficient number of studies.

Table S11: Sensitivity analysis and the level of evidence of the association of vegetables and vitamin C with RCC.

Exposure		No of primary studies	No. of participants / No. of cases	Adjusted SHR (95%-CI)	p-value	I^2	95%-PI	SSE	ESB	LSS	CES
Vegetables	full analysis	16	1365634/9353	0.74 (0.63 to 0.86)	0.000073	51.30%	0.44 to 1.23	Yes	No	Yes	III
	minus small-sized studies	13	1364783/9188	0.75 (0.64 to 0.89)	0.000807	56.60%	0.43 to 1.31	No	No	Yes	III
	minus low-quality studies	14	1364553/9223	0.72 (0.61 to 0.84)	0.000024	53.80%	0.44 to 1.17	Yes	No	Yes	III
Vitamin C	full analysis	10	274864/5182	0.77 (0.66 to 0.90)	0.000636	0.00%	0.54 to 1.10	No	No	No	III
	minus small-sized studies	7	272881/4175	0.80 (0.67 to 0.95)	0.009711	0.00%	0.52 to 1.22	No	No	No	IV
	minus low-quality studies	NC	NC	NC	NC	NC	NC	NC	NC	NC	NC

CI – confidence interval, PI – prediction interval, SSE – small study effect, ESB – excess significance bias, LSS – Largest study significant, CES – class of evidence after sensitivity analysis, NC – not calculable because the quality of individual studies included in the meta-analysis was not available.
