# Supplementary figures and images for: The role of diet in renal cell carcinoma incidence: an umbrella review of meta-analyses of observational studies
Source: BMC Med. 2022 Feb 3;20:39. doi: 10.1186/s12916-021-02229-5 (PMC8812002; doi:10.1186/s12916-021-02229-5)

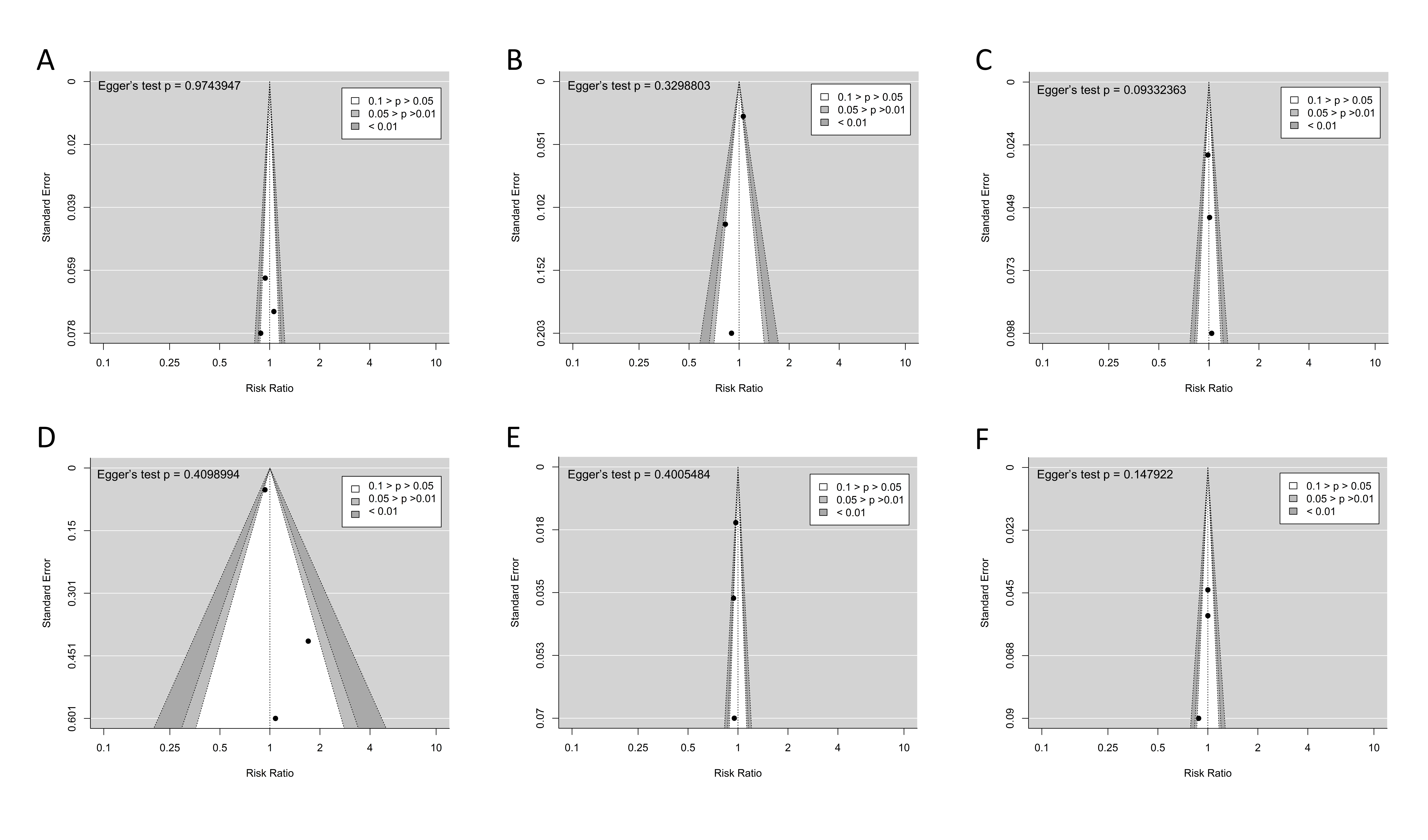

Supplement: Supplementary file 2 — Additional file 2: Figure S1. Contour-enhanced funnel plots associating dietary patterns or diet quality indices with RCC incidence: A) healthy dietary pattern, B) unhealthy/Western pattern, C) drinking pattern, D) dietary inflammatory index, E) glycemic index, and F) glycemic load. Figure S2. Contour-enhanced funnel plots associating foods with RCC incidence: A) fish, B) vegetables, C) fruits, D) red meat, E) processed meat, and F) cruciferous vegetables. Figure S3. Contour-enhanced funnel plots associating foods with RCC incidence: A) poultry, B) all meat, C) fruit and non-starchy vegetables, D) non-starchy vegetables, E) tomatoes, and F) citrus fruit. Figure S4. Contour-enhanced funnel plots associating beverages with RCC incidence: A) sweet beverages (including artificially sweetened beverages and sugar-sweetened beverages), B) tea, C) coffee, and D) sweetened carbonated beverage. Figure S5. Contour-enhanced funnel plots associating alcoholic beverages with RCC incidence: A) beer, B) wine, C) spirits, D) alcohol (light), E) alcohol (moderate), F) alcohol (heavy), and G) alcohol (any). Figure S6. Contour-enhanced funnel plots associating macronutrients with RCC incidence: A) fruit fiber, B) vegetable fiber, C) cereal fiber, D) dietary fiber, E) Total fat, and F) saturated fat. Figure S7. Contour-enhanced funnel plots associating macronutrients with RCC incidence: A) monounsaturated fat, B) polyunsaturated fat, C) total protein, D) animal protein, and E) plant protein. Figure S8. Contour-enhanced funnel plots associating micronutrients with RCC incidence: A) riboflavin, B) vitamin B6, C) folate, D) vitamin B12, E) methionine, and F) choline. Figure S9. Contour-enhanced funnel plots associating micronutrients with RCC incidence: A) betaine, B) vitamin C, C) vitamin D, D) dietary nitrate, E) dietary nitrite, and F) vitamin E. Figure S10. Contour-enhanced funnel plots associating micronutrients with RCC incidence: A) alpha-carotene, B) beta-cryptoxanthin, C) lutein a [file 12916_2021_2229_MOESM2_ESM.zip › Figs. S10R2.TIF]

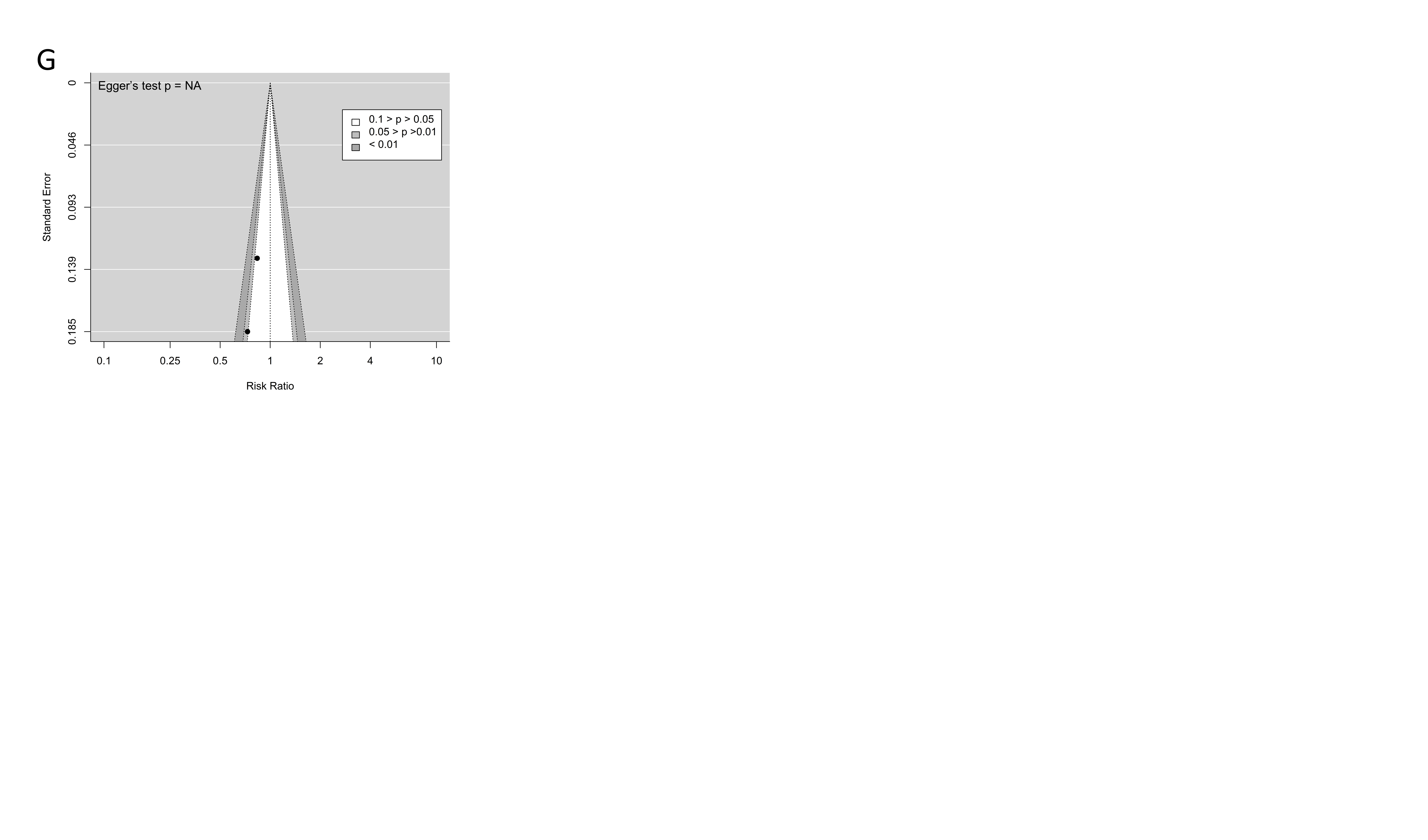

Supplement: Supplementary file 2 — Additional file 2: Figure S1. Contour-enhanced funnel plots associating dietary patterns or diet quality indices with RCC incidence: A) healthy dietary pattern, B) unhealthy/Western pattern, C) drinking pattern, D) dietary inflammatory index, E) glycemic index, and F) glycemic load. Figure S2. Contour-enhanced funnel plots associating foods with RCC incidence: A) fish, B) vegetables, C) fruits, D) red meat, E) processed meat, and F) cruciferous vegetables. Figure S3. Contour-enhanced funnel plots associating foods with RCC incidence: A) poultry, B) all meat, C) fruit and non-starchy vegetables, D) non-starchy vegetables, E) tomatoes, and F) citrus fruit. Figure S4. Contour-enhanced funnel plots associating beverages with RCC incidence: A) sweet beverages (including artificially sweetened beverages and sugar-sweetened beverages), B) tea, C) coffee, and D) sweetened carbonated beverage. Figure S5. Contour-enhanced funnel plots associating alcoholic beverages with RCC incidence: A) beer, B) wine, C) spirits, D) alcohol (light), E) alcohol (moderate), F) alcohol (heavy), and G) alcohol (any). Figure S6. Contour-enhanced funnel plots associating macronutrients with RCC incidence: A) fruit fiber, B) vegetable fiber, C) cereal fiber, D) dietary fiber, E) Total fat, and F) saturated fat. Figure S7. Contour-enhanced funnel plots associating macronutrients with RCC incidence: A) monounsaturated fat, B) polyunsaturated fat, C) total protein, D) animal protein, and E) plant protein. Figure S8. Contour-enhanced funnel plots associating micronutrients with RCC incidence: A) riboflavin, B) vitamin B6, C) folate, D) vitamin B12, E) methionine, and F) choline. Figure S9. Contour-enhanced funnel plots associating micronutrients with RCC incidence: A) betaine, B) vitamin C, C) vitamin D, D) dietary nitrate, E) dietary nitrite, and F) vitamin E. Figure S10. Contour-enhanced funnel plots associating micronutrients with RCC incidence: A) alpha-carotene, B) beta-cryptoxanthin, C) lutein a [file 12916_2021_2229_MOESM2_ESM.zip › Figs. S10_GR2.TIF]

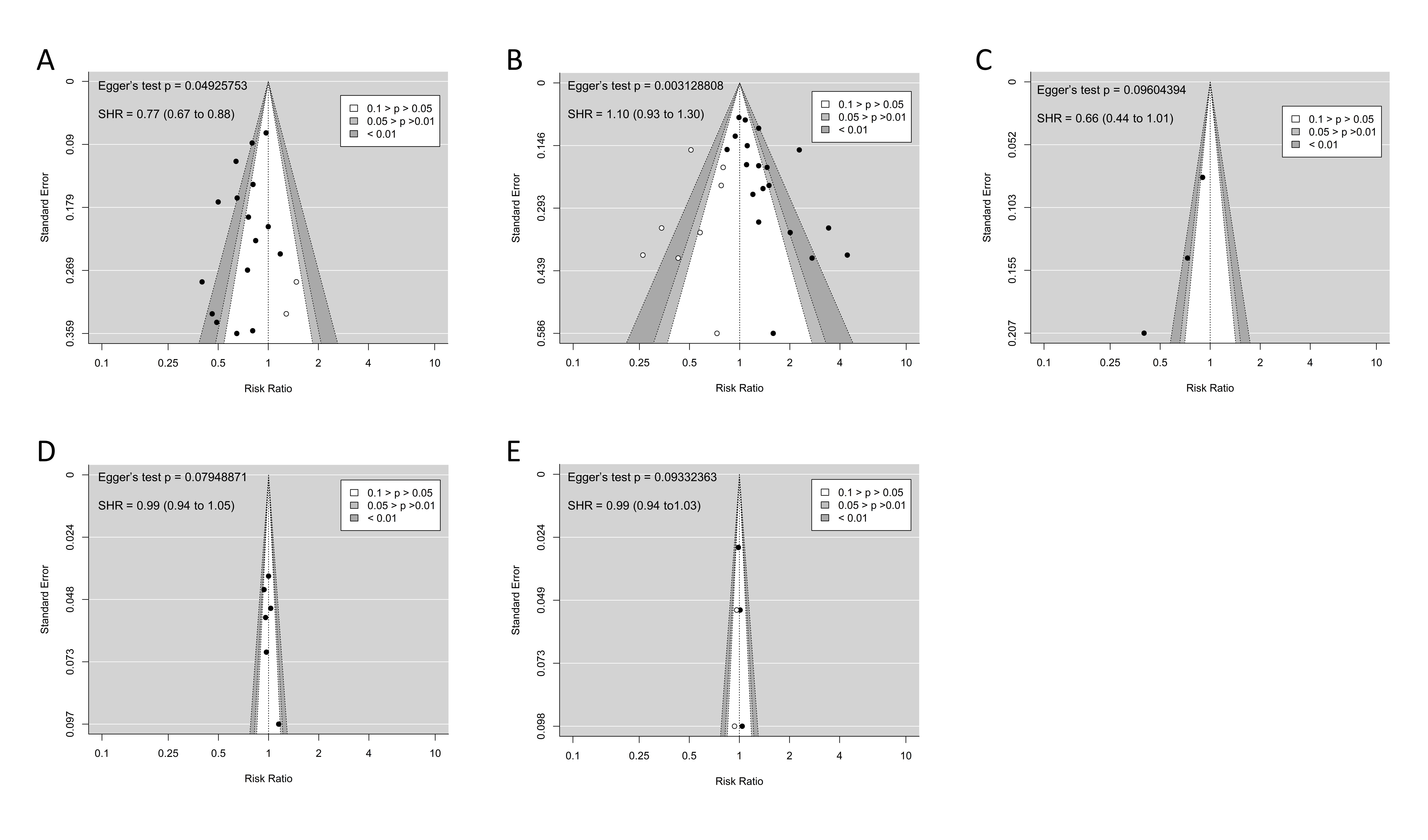

Supplement: Supplementary file 2 — Additional file 2: Figure S1. Contour-enhanced funnel plots associating dietary patterns or diet quality indices with RCC incidence: A) healthy dietary pattern, B) unhealthy/Western pattern, C) drinking pattern, D) dietary inflammatory index, E) glycemic index, and F) glycemic load. Figure S2. Contour-enhanced funnel plots associating foods with RCC incidence: A) fish, B) vegetables, C) fruits, D) red meat, E) processed meat, and F) cruciferous vegetables. Figure S3. Contour-enhanced funnel plots associating foods with RCC incidence: A) poultry, B) all meat, C) fruit and non-starchy vegetables, D) non-starchy vegetables, E) tomatoes, and F) citrus fruit. Figure S4. Contour-enhanced funnel plots associating beverages with RCC incidence: A) sweet beverages (including artificially sweetened beverages and sugar-sweetened beverages), B) tea, C) coffee, and D) sweetened carbonated beverage. Figure S5. Contour-enhanced funnel plots associating alcoholic beverages with RCC incidence: A) beer, B) wine, C) spirits, D) alcohol (light), E) alcohol (moderate), F) alcohol (heavy), and G) alcohol (any). Figure S6. Contour-enhanced funnel plots associating macronutrients with RCC incidence: A) fruit fiber, B) vegetable fiber, C) cereal fiber, D) dietary fiber, E) Total fat, and F) saturated fat. Figure S7. Contour-enhanced funnel plots associating macronutrients with RCC incidence: A) monounsaturated fat, B) polyunsaturated fat, C) total protein, D) animal protein, and E) plant protein. Figure S8. Contour-enhanced funnel plots associating micronutrients with RCC incidence: A) riboflavin, B) vitamin B6, C) folate, D) vitamin B12, E) methionine, and F) choline. Figure S9. Contour-enhanced funnel plots associating micronutrients with RCC incidence: A) betaine, B) vitamin C, C) vitamin D, D) dietary nitrate, E) dietary nitrite, and F) vitamin E. Figure S10. Contour-enhanced funnel plots associating micronutrients with RCC incidence: A) alpha-carotene, B) beta-cryptoxanthin, C) lutein a [file 12916_2021_2229_MOESM2_ESM.zip › Figs. S11R2.TIF]

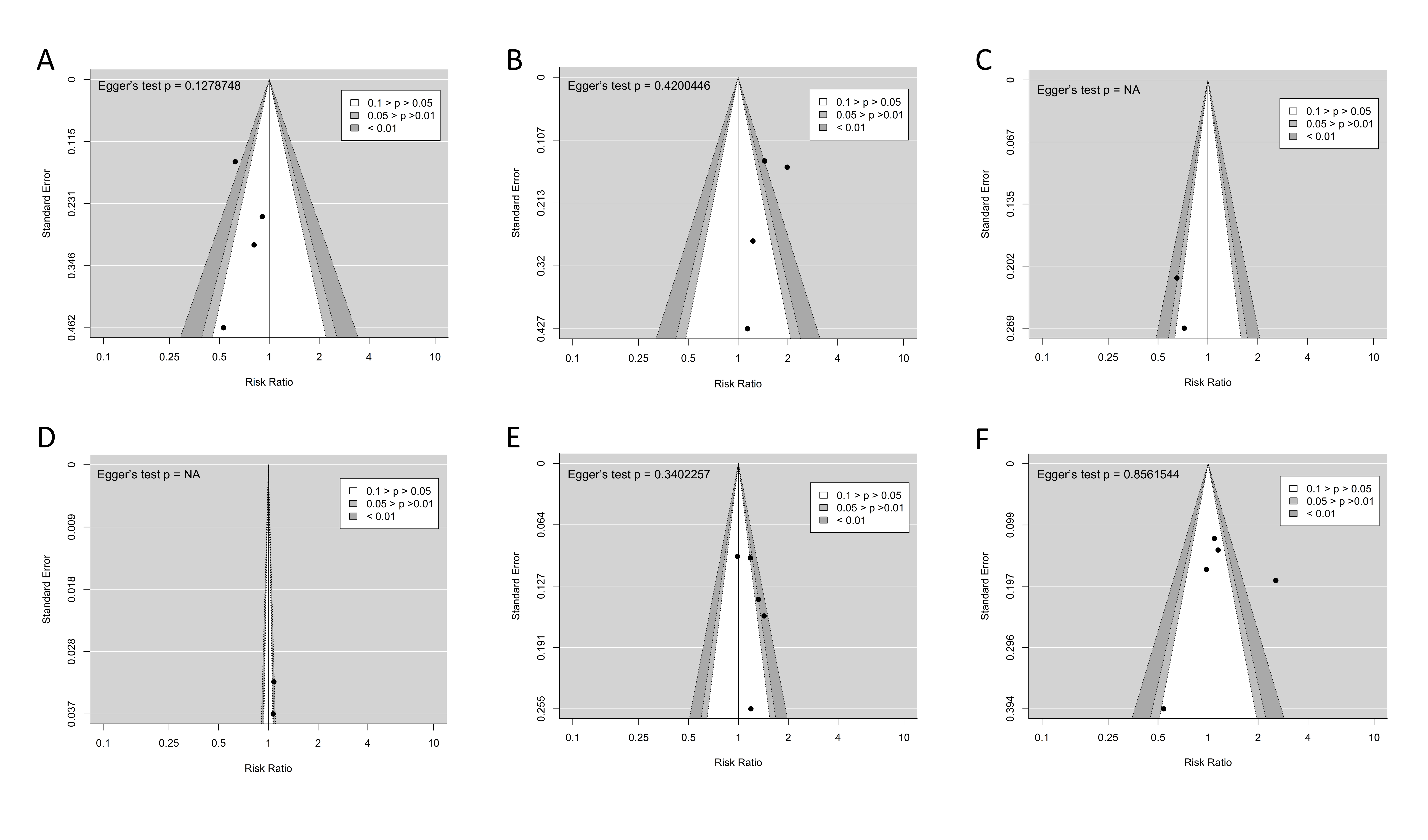

Supplement: Supplementary file 2 — Additional file 2: Figure S1. Contour-enhanced funnel plots associating dietary patterns or diet quality indices with RCC incidence: A) healthy dietary pattern, B) unhealthy/Western pattern, C) drinking pattern, D) dietary inflammatory index, E) glycemic index, and F) glycemic load. Figure S2. Contour-enhanced funnel plots associating foods with RCC incidence: A) fish, B) vegetables, C) fruits, D) red meat, E) processed meat, and F) cruciferous vegetables. Figure S3. Contour-enhanced funnel plots associating foods with RCC incidence: A) poultry, B) all meat, C) fruit and non-starchy vegetables, D) non-starchy vegetables, E) tomatoes, and F) citrus fruit. Figure S4. Contour-enhanced funnel plots associating beverages with RCC incidence: A) sweet beverages (including artificially sweetened beverages and sugar-sweetened beverages), B) tea, C) coffee, and D) sweetened carbonated beverage. Figure S5. Contour-enhanced funnel plots associating alcoholic beverages with RCC incidence: A) beer, B) wine, C) spirits, D) alcohol (light), E) alcohol (moderate), F) alcohol (heavy), and G) alcohol (any). Figure S6. Contour-enhanced funnel plots associating macronutrients with RCC incidence: A) fruit fiber, B) vegetable fiber, C) cereal fiber, D) dietary fiber, E) Total fat, and F) saturated fat. Figure S7. Contour-enhanced funnel plots associating macronutrients with RCC incidence: A) monounsaturated fat, B) polyunsaturated fat, C) total protein, D) animal protein, and E) plant protein. Figure S8. Contour-enhanced funnel plots associating micronutrients with RCC incidence: A) riboflavin, B) vitamin B6, C) folate, D) vitamin B12, E) methionine, and F) choline. Figure S9. Contour-enhanced funnel plots associating micronutrients with RCC incidence: A) betaine, B) vitamin C, C) vitamin D, D) dietary nitrate, E) dietary nitrite, and F) vitamin E. Figure S10. Contour-enhanced funnel plots associating micronutrients with RCC incidence: A) alpha-carotene, B) beta-cryptoxanthin, C) lutein a [file 12916_2021_2229_MOESM2_ESM.zip › Figs. S1R2.tif]

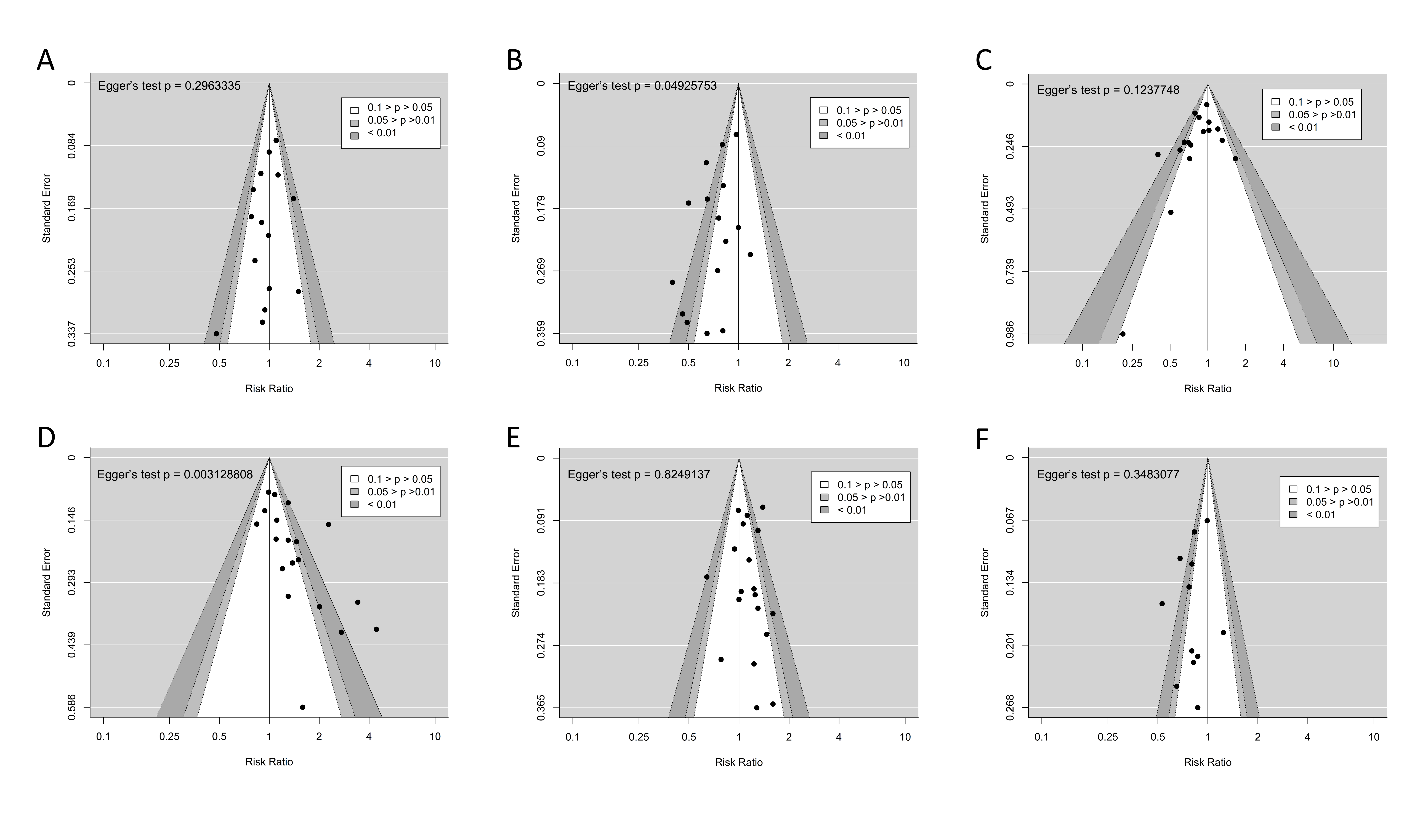

Supplement: Supplementary file 2 — Additional file 2: Figure S1. Contour-enhanced funnel plots associating dietary patterns or diet quality indices with RCC incidence: A) healthy dietary pattern, B) unhealthy/Western pattern, C) drinking pattern, D) dietary inflammatory index, E) glycemic index, and F) glycemic load. Figure S2. Contour-enhanced funnel plots associating foods with RCC incidence: A) fish, B) vegetables, C) fruits, D) red meat, E) processed meat, and F) cruciferous vegetables. Figure S3. Contour-enhanced funnel plots associating foods with RCC incidence: A) poultry, B) all meat, C) fruit and non-starchy vegetables, D) non-starchy vegetables, E) tomatoes, and F) citrus fruit. Figure S4. Contour-enhanced funnel plots associating beverages with RCC incidence: A) sweet beverages (including artificially sweetened beverages and sugar-sweetened beverages), B) tea, C) coffee, and D) sweetened carbonated beverage. Figure S5. Contour-enhanced funnel plots associating alcoholic beverages with RCC incidence: A) beer, B) wine, C) spirits, D) alcohol (light), E) alcohol (moderate), F) alcohol (heavy), and G) alcohol (any). Figure S6. Contour-enhanced funnel plots associating macronutrients with RCC incidence: A) fruit fiber, B) vegetable fiber, C) cereal fiber, D) dietary fiber, E) Total fat, and F) saturated fat. Figure S7. Contour-enhanced funnel plots associating macronutrients with RCC incidence: A) monounsaturated fat, B) polyunsaturated fat, C) total protein, D) animal protein, and E) plant protein. Figure S8. Contour-enhanced funnel plots associating micronutrients with RCC incidence: A) riboflavin, B) vitamin B6, C) folate, D) vitamin B12, E) methionine, and F) choline. Figure S9. Contour-enhanced funnel plots associating micronutrients with RCC incidence: A) betaine, B) vitamin C, C) vitamin D, D) dietary nitrate, E) dietary nitrite, and F) vitamin E. Figure S10. Contour-enhanced funnel plots associating micronutrients with RCC incidence: A) alpha-carotene, B) beta-cryptoxanthin, C) lutein a [file 12916_2021_2229_MOESM2_ESM.zip › Figs. S2R2.TIF]

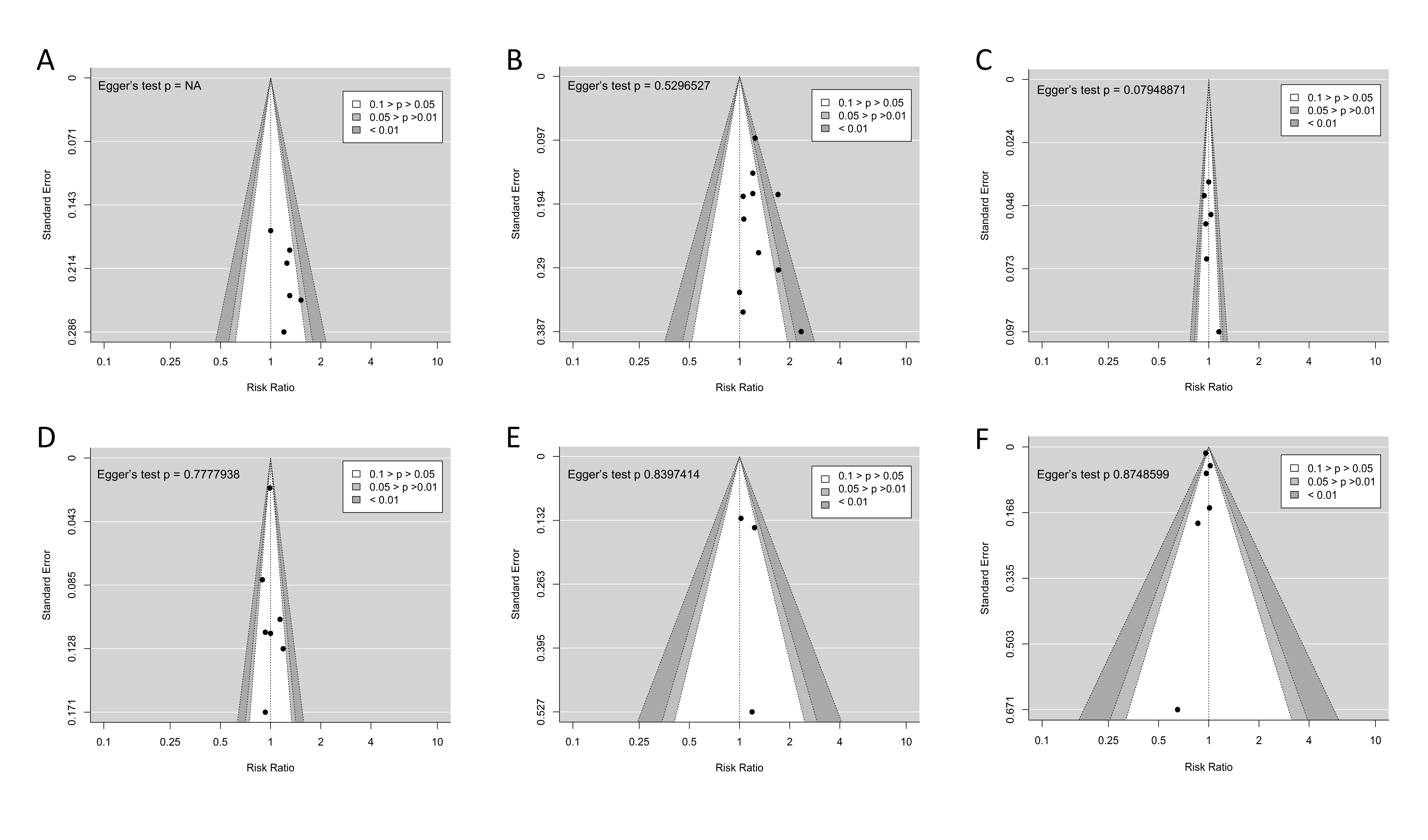

Supplement: Supplementary file 2 — Additional file 2: Figure S1. Contour-enhanced funnel plots associating dietary patterns or diet quality indices with RCC incidence: A) healthy dietary pattern, B) unhealthy/Western pattern, C) drinking pattern, D) dietary inflammatory index, E) glycemic index, and F) glycemic load. Figure S2. Contour-enhanced funnel plots associating foods with RCC incidence: A) fish, B) vegetables, C) fruits, D) red meat, E) processed meat, and F) cruciferous vegetables. Figure S3. Contour-enhanced funnel plots associating foods with RCC incidence: A) poultry, B) all meat, C) fruit and non-starchy vegetables, D) non-starchy vegetables, E) tomatoes, and F) citrus fruit. Figure S4. Contour-enhanced funnel plots associating beverages with RCC incidence: A) sweet beverages (including artificially sweetened beverages and sugar-sweetened beverages), B) tea, C) coffee, and D) sweetened carbonated beverage. Figure S5. Contour-enhanced funnel plots associating alcoholic beverages with RCC incidence: A) beer, B) wine, C) spirits, D) alcohol (light), E) alcohol (moderate), F) alcohol (heavy), and G) alcohol (any). Figure S6. Contour-enhanced funnel plots associating macronutrients with RCC incidence: A) fruit fiber, B) vegetable fiber, C) cereal fiber, D) dietary fiber, E) Total fat, and F) saturated fat. Figure S7. Contour-enhanced funnel plots associating macronutrients with RCC incidence: A) monounsaturated fat, B) polyunsaturated fat, C) total protein, D) animal protein, and E) plant protein. Figure S8. Contour-enhanced funnel plots associating micronutrients with RCC incidence: A) riboflavin, B) vitamin B6, C) folate, D) vitamin B12, E) methionine, and F) choline. Figure S9. Contour-enhanced funnel plots associating micronutrients with RCC incidence: A) betaine, B) vitamin C, C) vitamin D, D) dietary nitrate, E) dietary nitrite, and F) vitamin E. Figure S10. Contour-enhanced funnel plots associating micronutrients with RCC incidence: A) alpha-carotene, B) beta-cryptoxanthin, C) lutein a [file 12916_2021_2229_MOESM2_ESM.zip › Figs. S3R2.TIF]

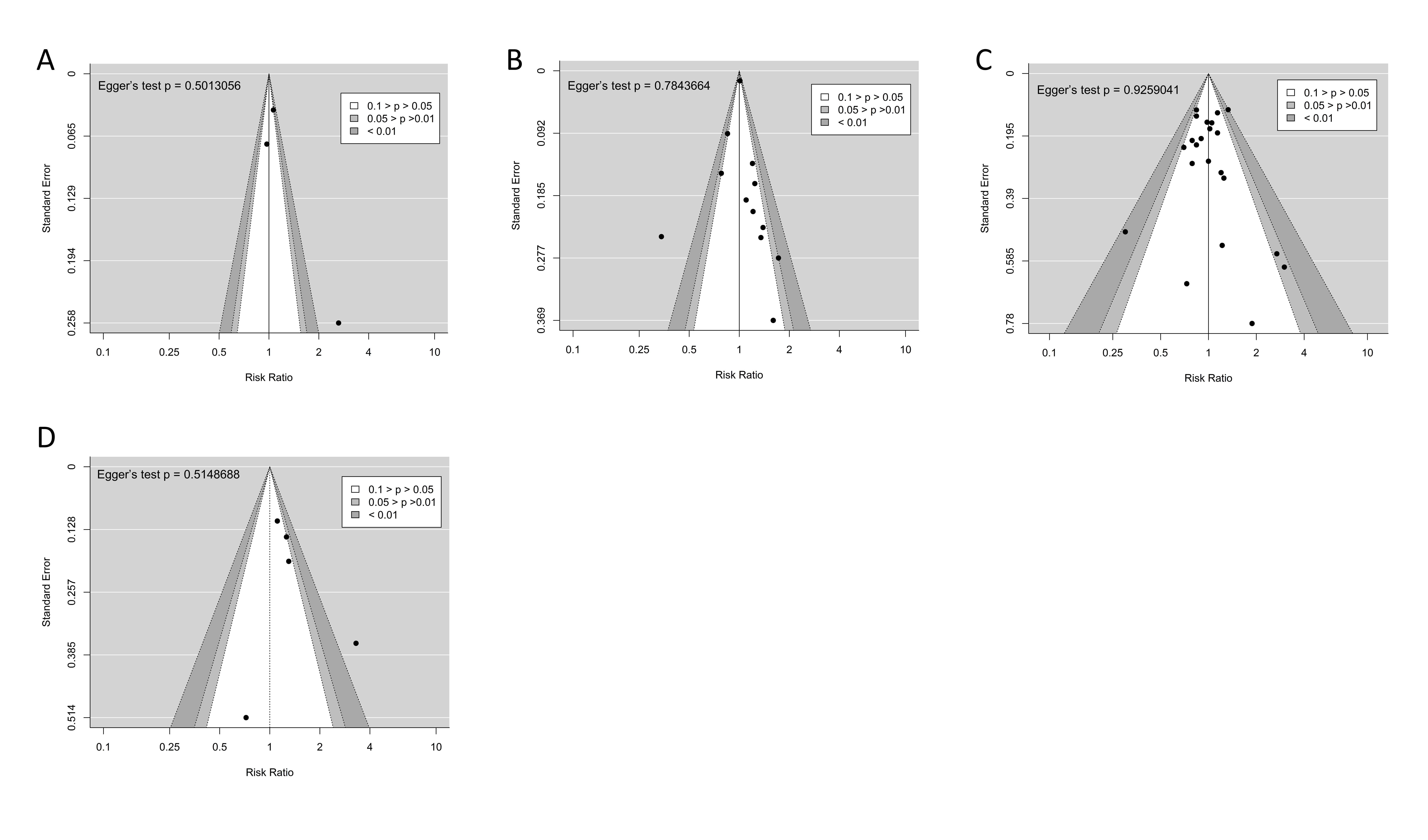

Supplement: Supplementary file 2 — Additional file 2: Figure S1. Contour-enhanced funnel plots associating dietary patterns or diet quality indices with RCC incidence: A) healthy dietary pattern, B) unhealthy/Western pattern, C) drinking pattern, D) dietary inflammatory index, E) glycemic index, and F) glycemic load. Figure S2. Contour-enhanced funnel plots associating foods with RCC incidence: A) fish, B) vegetables, C) fruits, D) red meat, E) processed meat, and F) cruciferous vegetables. Figure S3. Contour-enhanced funnel plots associating foods with RCC incidence: A) poultry, B) all meat, C) fruit and non-starchy vegetables, D) non-starchy vegetables, E) tomatoes, and F) citrus fruit. Figure S4. Contour-enhanced funnel plots associating beverages with RCC incidence: A) sweet beverages (including artificially sweetened beverages and sugar-sweetened beverages), B) tea, C) coffee, and D) sweetened carbonated beverage. Figure S5. Contour-enhanced funnel plots associating alcoholic beverages with RCC incidence: A) beer, B) wine, C) spirits, D) alcohol (light), E) alcohol (moderate), F) alcohol (heavy), and G) alcohol (any). Figure S6. Contour-enhanced funnel plots associating macronutrients with RCC incidence: A) fruit fiber, B) vegetable fiber, C) cereal fiber, D) dietary fiber, E) Total fat, and F) saturated fat. Figure S7. Contour-enhanced funnel plots associating macronutrients with RCC incidence: A) monounsaturated fat, B) polyunsaturated fat, C) total protein, D) animal protein, and E) plant protein. Figure S8. Contour-enhanced funnel plots associating micronutrients with RCC incidence: A) riboflavin, B) vitamin B6, C) folate, D) vitamin B12, E) methionine, and F) choline. Figure S9. Contour-enhanced funnel plots associating micronutrients with RCC incidence: A) betaine, B) vitamin C, C) vitamin D, D) dietary nitrate, E) dietary nitrite, and F) vitamin E. Figure S10. Contour-enhanced funnel plots associating micronutrients with RCC incidence: A) alpha-carotene, B) beta-cryptoxanthin, C) lutein a [file 12916_2021_2229_MOESM2_ESM.zip › Figs. S4R2.tif]

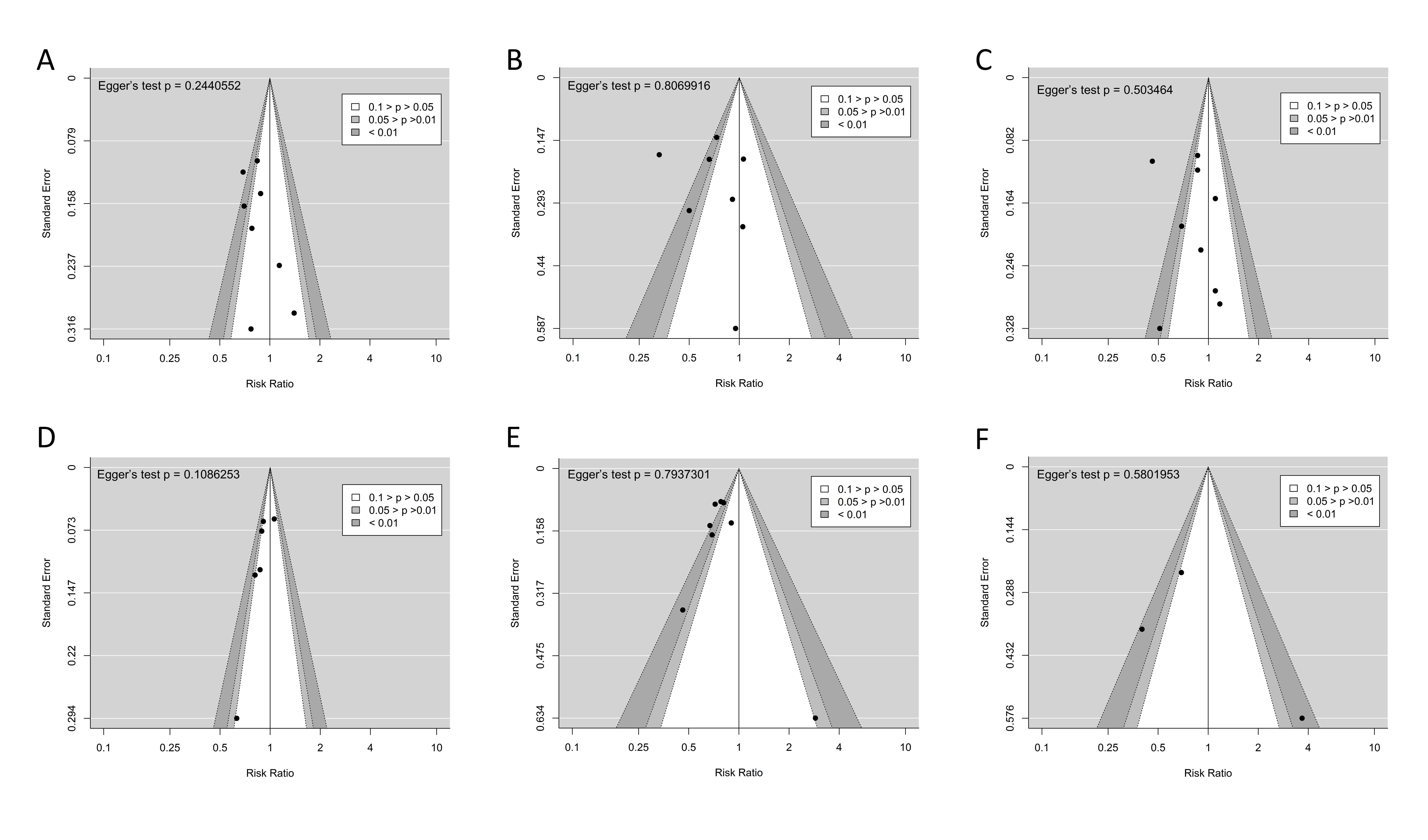

Supplement: Supplementary file 2 — Additional file 2: Figure S1. Contour-enhanced funnel plots associating dietary patterns or diet quality indices with RCC incidence: A) healthy dietary pattern, B) unhealthy/Western pattern, C) drinking pattern, D) dietary inflammatory index, E) glycemic index, and F) glycemic load. Figure S2. Contour-enhanced funnel plots associating foods with RCC incidence: A) fish, B) vegetables, C) fruits, D) red meat, E) processed meat, and F) cruciferous vegetables. Figure S3. Contour-enhanced funnel plots associating foods with RCC incidence: A) poultry, B) all meat, C) fruit and non-starchy vegetables, D) non-starchy vegetables, E) tomatoes, and F) citrus fruit. Figure S4. Contour-enhanced funnel plots associating beverages with RCC incidence: A) sweet beverages (including artificially sweetened beverages and sugar-sweetened beverages), B) tea, C) coffee, and D) sweetened carbonated beverage. Figure S5. Contour-enhanced funnel plots associating alcoholic beverages with RCC incidence: A) beer, B) wine, C) spirits, D) alcohol (light), E) alcohol (moderate), F) alcohol (heavy), and G) alcohol (any). Figure S6. Contour-enhanced funnel plots associating macronutrients with RCC incidence: A) fruit fiber, B) vegetable fiber, C) cereal fiber, D) dietary fiber, E) Total fat, and F) saturated fat. Figure S7. Contour-enhanced funnel plots associating macronutrients with RCC incidence: A) monounsaturated fat, B) polyunsaturated fat, C) total protein, D) animal protein, and E) plant protein. Figure S8. Contour-enhanced funnel plots associating micronutrients with RCC incidence: A) riboflavin, B) vitamin B6, C) folate, D) vitamin B12, E) methionine, and F) choline. Figure S9. Contour-enhanced funnel plots associating micronutrients with RCC incidence: A) betaine, B) vitamin C, C) vitamin D, D) dietary nitrate, E) dietary nitrite, and F) vitamin E. Figure S10. Contour-enhanced funnel plots associating micronutrients with RCC incidence: A) alpha-carotene, B) beta-cryptoxanthin, C) lutein a [file 12916_2021_2229_MOESM2_ESM.zip › Figs. S5R2.TIF]

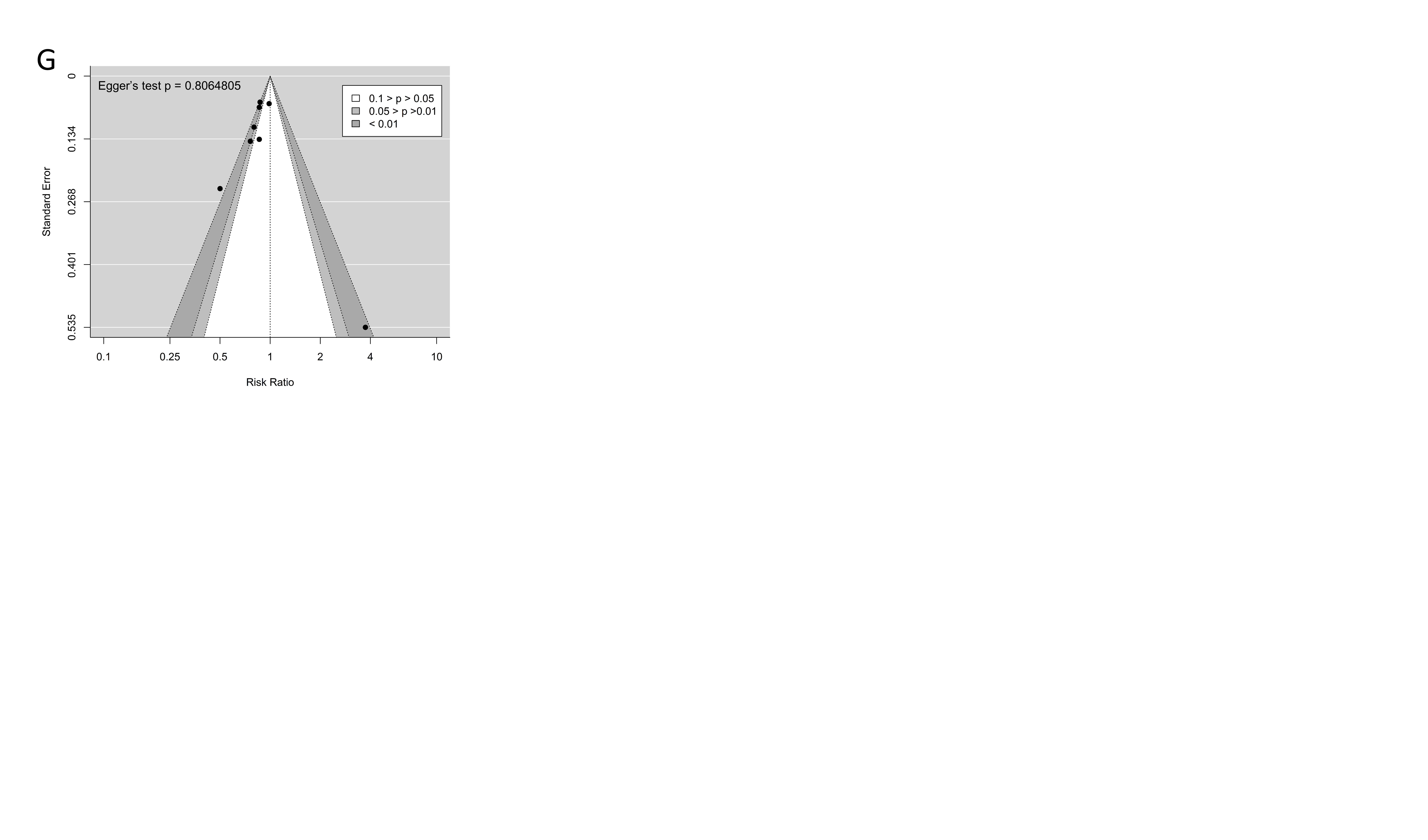

Supplement: Supplementary file 2 — Additional file 2: Figure S1. Contour-enhanced funnel plots associating dietary patterns or diet quality indices with RCC incidence: A) healthy dietary pattern, B) unhealthy/Western pattern, C) drinking pattern, D) dietary inflammatory index, E) glycemic index, and F) glycemic load. Figure S2. Contour-enhanced funnel plots associating foods with RCC incidence: A) fish, B) vegetables, C) fruits, D) red meat, E) processed meat, and F) cruciferous vegetables. Figure S3. Contour-enhanced funnel plots associating foods with RCC incidence: A) poultry, B) all meat, C) fruit and non-starchy vegetables, D) non-starchy vegetables, E) tomatoes, and F) citrus fruit. Figure S4. Contour-enhanced funnel plots associating beverages with RCC incidence: A) sweet beverages (including artificially sweetened beverages and sugar-sweetened beverages), B) tea, C) coffee, and D) sweetened carbonated beverage. Figure S5. Contour-enhanced funnel plots associating alcoholic beverages with RCC incidence: A) beer, B) wine, C) spirits, D) alcohol (light), E) alcohol (moderate), F) alcohol (heavy), and G) alcohol (any). Figure S6. Contour-enhanced funnel plots associating macronutrients with RCC incidence: A) fruit fiber, B) vegetable fiber, C) cereal fiber, D) dietary fiber, E) Total fat, and F) saturated fat. Figure S7. Contour-enhanced funnel plots associating macronutrients with RCC incidence: A) monounsaturated fat, B) polyunsaturated fat, C) total protein, D) animal protein, and E) plant protein. Figure S8. Contour-enhanced funnel plots associating micronutrients with RCC incidence: A) riboflavin, B) vitamin B6, C) folate, D) vitamin B12, E) methionine, and F) choline. Figure S9. Contour-enhanced funnel plots associating micronutrients with RCC incidence: A) betaine, B) vitamin C, C) vitamin D, D) dietary nitrate, E) dietary nitrite, and F) vitamin E. Figure S10. Contour-enhanced funnel plots associating micronutrients with RCC incidence: A) alpha-carotene, B) beta-cryptoxanthin, C) lutein a [file 12916_2021_2229_MOESM2_ESM.zip › Figs. S5_GR2.TIF]

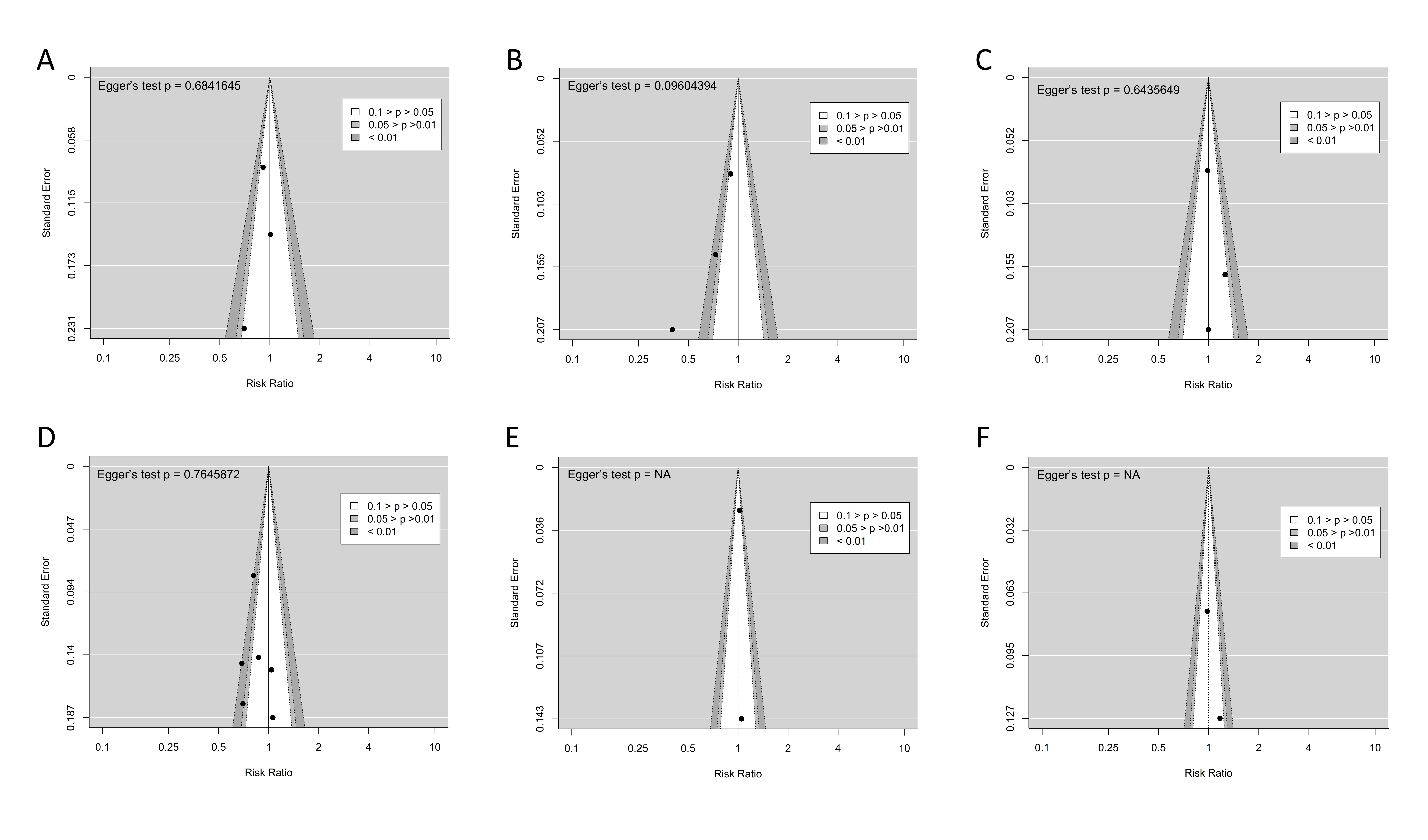

Supplement: Supplementary file 2 — Additional file 2: Figure S1. Contour-enhanced funnel plots associating dietary patterns or diet quality indices with RCC incidence: A) healthy dietary pattern, B) unhealthy/Western pattern, C) drinking pattern, D) dietary inflammatory index, E) glycemic index, and F) glycemic load. Figure S2. Contour-enhanced funnel plots associating foods with RCC incidence: A) fish, B) vegetables, C) fruits, D) red meat, E) processed meat, and F) cruciferous vegetables. Figure S3. Contour-enhanced funnel plots associating foods with RCC incidence: A) poultry, B) all meat, C) fruit and non-starchy vegetables, D) non-starchy vegetables, E) tomatoes, and F) citrus fruit. Figure S4. Contour-enhanced funnel plots associating beverages with RCC incidence: A) sweet beverages (including artificially sweetened beverages and sugar-sweetened beverages), B) tea, C) coffee, and D) sweetened carbonated beverage. Figure S5. Contour-enhanced funnel plots associating alcoholic beverages with RCC incidence: A) beer, B) wine, C) spirits, D) alcohol (light), E) alcohol (moderate), F) alcohol (heavy), and G) alcohol (any). Figure S6. Contour-enhanced funnel plots associating macronutrients with RCC incidence: A) fruit fiber, B) vegetable fiber, C) cereal fiber, D) dietary fiber, E) Total fat, and F) saturated fat. Figure S7. Contour-enhanced funnel plots associating macronutrients with RCC incidence: A) monounsaturated fat, B) polyunsaturated fat, C) total protein, D) animal protein, and E) plant protein. Figure S8. Contour-enhanced funnel plots associating micronutrients with RCC incidence: A) riboflavin, B) vitamin B6, C) folate, D) vitamin B12, E) methionine, and F) choline. Figure S9. Contour-enhanced funnel plots associating micronutrients with RCC incidence: A) betaine, B) vitamin C, C) vitamin D, D) dietary nitrate, E) dietary nitrite, and F) vitamin E. Figure S10. Contour-enhanced funnel plots associating micronutrients with RCC incidence: A) alpha-carotene, B) beta-cryptoxanthin, C) lutein a [file 12916_2021_2229_MOESM2_ESM.zip › Figs. S6R2.TIF]

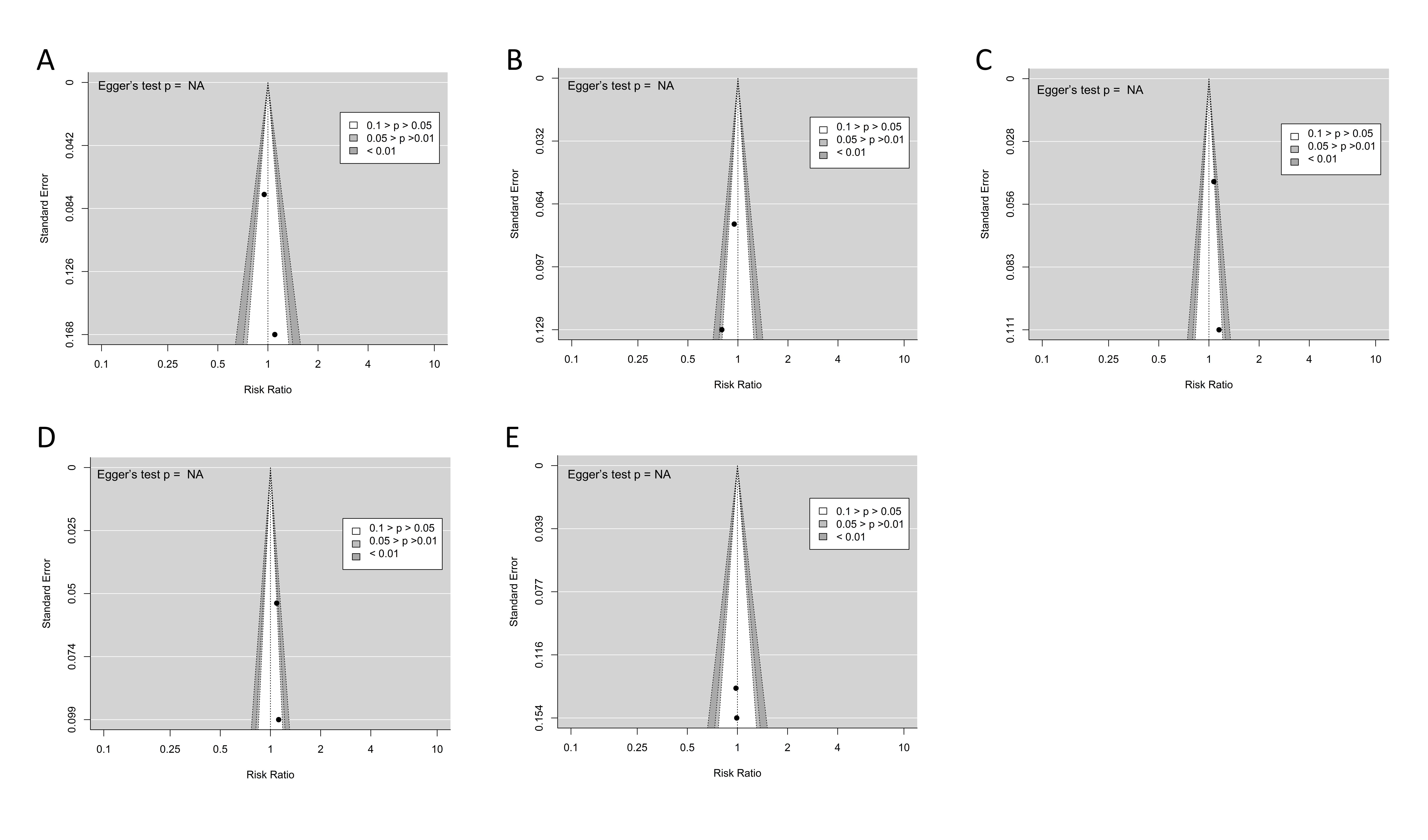

Supplement: Supplementary file 2 — Additional file 2: Figure S1. Contour-enhanced funnel plots associating dietary patterns or diet quality indices with RCC incidence: A) healthy dietary pattern, B) unhealthy/Western pattern, C) drinking pattern, D) dietary inflammatory index, E) glycemic index, and F) glycemic load. Figure S2. Contour-enhanced funnel plots associating foods with RCC incidence: A) fish, B) vegetables, C) fruits, D) red meat, E) processed meat, and F) cruciferous vegetables. Figure S3. Contour-enhanced funnel plots associating foods with RCC incidence: A) poultry, B) all meat, C) fruit and non-starchy vegetables, D) non-starchy vegetables, E) tomatoes, and F) citrus fruit. Figure S4. Contour-enhanced funnel plots associating beverages with RCC incidence: A) sweet beverages (including artificially sweetened beverages and sugar-sweetened beverages), B) tea, C) coffee, and D) sweetened carbonated beverage. Figure S5. Contour-enhanced funnel plots associating alcoholic beverages with RCC incidence: A) beer, B) wine, C) spirits, D) alcohol (light), E) alcohol (moderate), F) alcohol (heavy), and G) alcohol (any). Figure S6. Contour-enhanced funnel plots associating macronutrients with RCC incidence: A) fruit fiber, B) vegetable fiber, C) cereal fiber, D) dietary fiber, E) Total fat, and F) saturated fat. Figure S7. Contour-enhanced funnel plots associating macronutrients with RCC incidence: A) monounsaturated fat, B) polyunsaturated fat, C) total protein, D) animal protein, and E) plant protein. Figure S8. Contour-enhanced funnel plots associating micronutrients with RCC incidence: A) riboflavin, B) vitamin B6, C) folate, D) vitamin B12, E) methionine, and F) choline. Figure S9. Contour-enhanced funnel plots associating micronutrients with RCC incidence: A) betaine, B) vitamin C, C) vitamin D, D) dietary nitrate, E) dietary nitrite, and F) vitamin E. Figure S10. Contour-enhanced funnel plots associating micronutrients with RCC incidence: A) alpha-carotene, B) beta-cryptoxanthin, C) lutein a [file 12916_2021_2229_MOESM2_ESM.zip › Figs. S7R2.TIF]

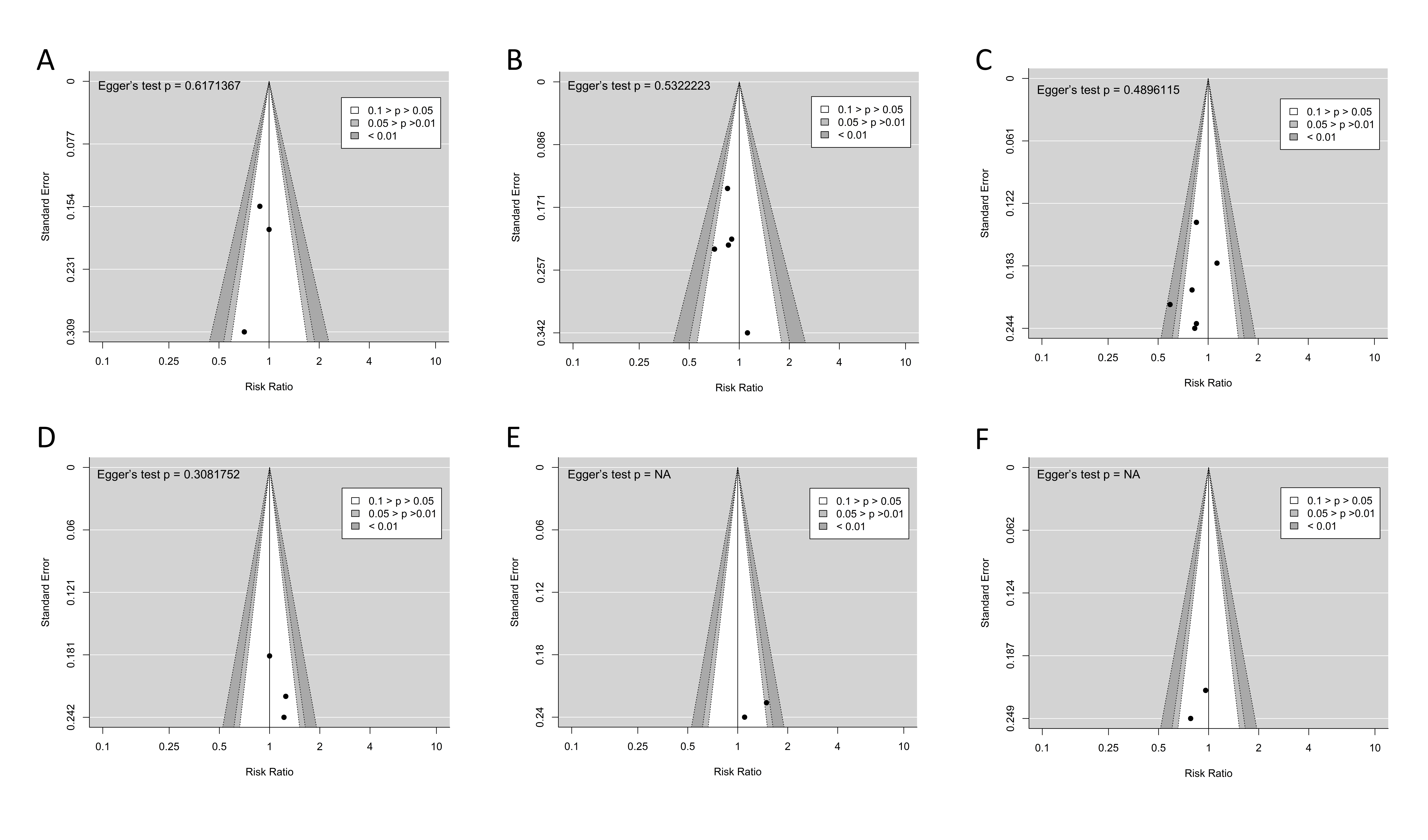

Supplement: Supplementary file 2 — Additional file 2: Figure S1. Contour-enhanced funnel plots associating dietary patterns or diet quality indices with RCC incidence: A) healthy dietary pattern, B) unhealthy/Western pattern, C) drinking pattern, D) dietary inflammatory index, E) glycemic index, and F) glycemic load. Figure S2. Contour-enhanced funnel plots associating foods with RCC incidence: A) fish, B) vegetables, C) fruits, D) red meat, E) processed meat, and F) cruciferous vegetables. Figure S3. Contour-enhanced funnel plots associating foods with RCC incidence: A) poultry, B) all meat, C) fruit and non-starchy vegetables, D) non-starchy vegetables, E) tomatoes, and F) citrus fruit. Figure S4. Contour-enhanced funnel plots associating beverages with RCC incidence: A) sweet beverages (including artificially sweetened beverages and sugar-sweetened beverages), B) tea, C) coffee, and D) sweetened carbonated beverage. Figure S5. Contour-enhanced funnel plots associating alcoholic beverages with RCC incidence: A) beer, B) wine, C) spirits, D) alcohol (light), E) alcohol (moderate), F) alcohol (heavy), and G) alcohol (any). Figure S6. Contour-enhanced funnel plots associating macronutrients with RCC incidence: A) fruit fiber, B) vegetable fiber, C) cereal fiber, D) dietary fiber, E) Total fat, and F) saturated fat. Figure S7. Contour-enhanced funnel plots associating macronutrients with RCC incidence: A) monounsaturated fat, B) polyunsaturated fat, C) total protein, D) animal protein, and E) plant protein. Figure S8. Contour-enhanced funnel plots associating micronutrients with RCC incidence: A) riboflavin, B) vitamin B6, C) folate, D) vitamin B12, E) methionine, and F) choline. Figure S9. Contour-enhanced funnel plots associating micronutrients with RCC incidence: A) betaine, B) vitamin C, C) vitamin D, D) dietary nitrate, E) dietary nitrite, and F) vitamin E. Figure S10. Contour-enhanced funnel plots associating micronutrients with RCC incidence: A) alpha-carotene, B) beta-cryptoxanthin, C) lutein a [file 12916_2021_2229_MOESM2_ESM.zip › Figs. S8R2.TIF]

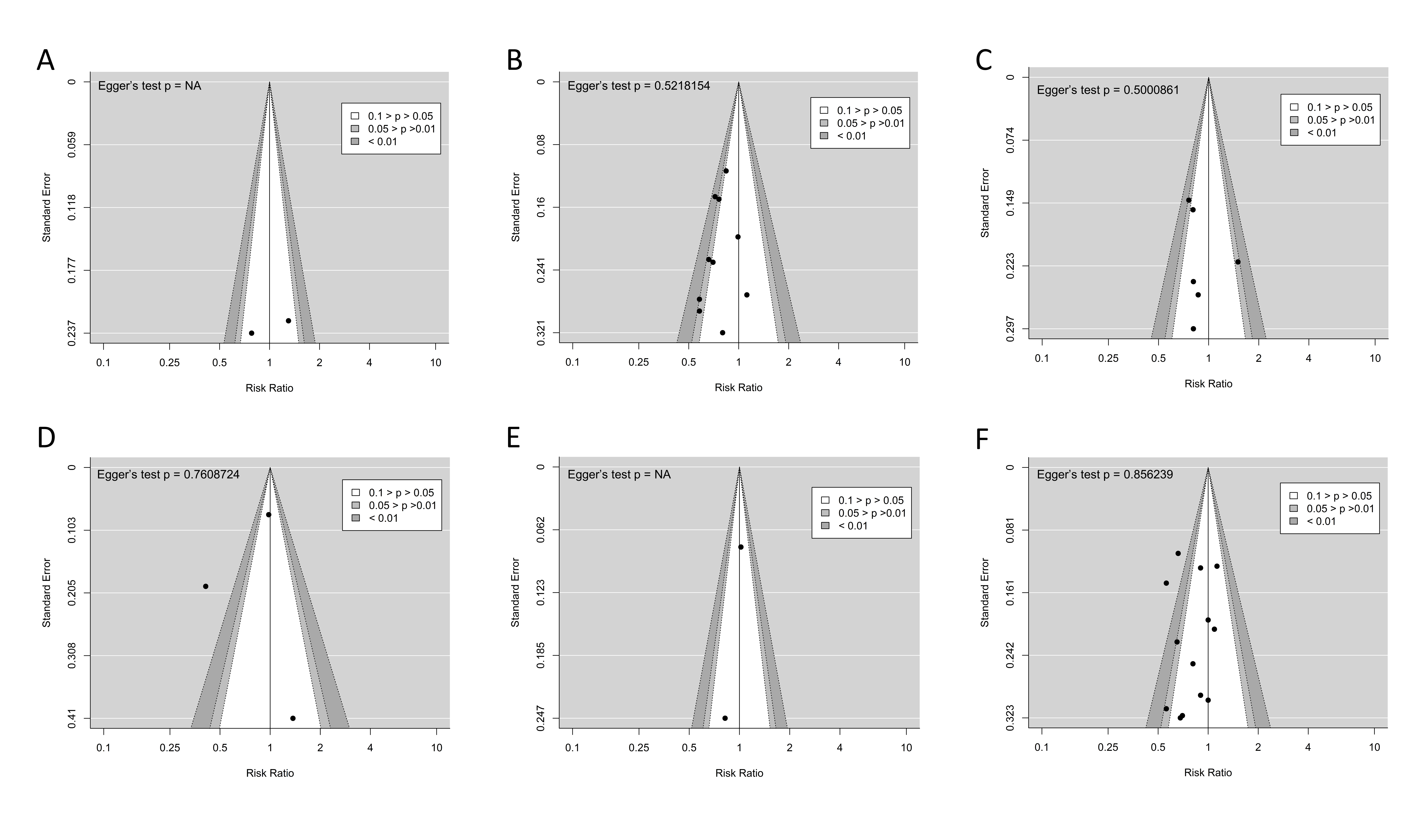

Supplement: Supplementary file 2 — Additional file 2: Figure S1. Contour-enhanced funnel plots associating dietary patterns or diet quality indices with RCC incidence: A) healthy dietary pattern, B) unhealthy/Western pattern, C) drinking pattern, D) dietary inflammatory index, E) glycemic index, and F) glycemic load. Figure S2. Contour-enhanced funnel plots associating foods with RCC incidence: A) fish, B) vegetables, C) fruits, D) red meat, E) processed meat, and F) cruciferous vegetables. Figure S3. Contour-enhanced funnel plots associating foods with RCC incidence: A) poultry, B) all meat, C) fruit and non-starchy vegetables, D) non-starchy vegetables, E) tomatoes, and F) citrus fruit. Figure S4. Contour-enhanced funnel plots associating beverages with RCC incidence: A) sweet beverages (including artificially sweetened beverages and sugar-sweetened beverages), B) tea, C) coffee, and D) sweetened carbonated beverage. Figure S5. Contour-enhanced funnel plots associating alcoholic beverages with RCC incidence: A) beer, B) wine, C) spirits, D) alcohol (light), E) alcohol (moderate), F) alcohol (heavy), and G) alcohol (any). Figure S6. Contour-enhanced funnel plots associating macronutrients with RCC incidence: A) fruit fiber, B) vegetable fiber, C) cereal fiber, D) dietary fiber, E) Total fat, and F) saturated fat. Figure S7. Contour-enhanced funnel plots associating macronutrients with RCC incidence: A) monounsaturated fat, B) polyunsaturated fat, C) total protein, D) animal protein, and E) plant protein. Figure S8. Contour-enhanced funnel plots associating micronutrients with RCC incidence: A) riboflavin, B) vitamin B6, C) folate, D) vitamin B12, E) methionine, and F) choline. Figure S9. Contour-enhanced funnel plots associating micronutrients with RCC incidence: A) betaine, B) vitamin C, C) vitamin D, D) dietary nitrate, E) dietary nitrite, and F) vitamin E. Figure S10. Contour-enhanced funnel plots associating micronutrients with RCC incidence: A) alpha-carotene, B) beta-cryptoxanthin, C) lutein a [file 12916_2021_2229_MOESM2_ESM.zip › Figs. S9R2.TIF]
